# Fatuamide A, a Hybrid PKS/NRPS Metallophore from a *Leptolyngbya* sp. Marine Cyanobacterium Collected in American Samoa

**DOI:** 10.1021/acs.jnatprod.4c01051

**Published:** 2025-01-29

**Authors:** Kelsey L. Alexander, C. Benjamin Naman, Arihiro Iwasaki, Alfonso Mangoni, Tiago Leao, Raphael Reher, Daniel Petras, Hyunwoo Kim, Eva Ternon, Eduardo J. E. Caro-Diaz, Evgenia Glukhov, Jana A. Mitrevska, Nicole E. Avalon, Brendan M. Duggan, Lena Gerwick, William H. Gerwick

**Affiliations:** 1 Center for Marine Biotechnology and Biomedicine, Scripps Institution of Oceanography, University of California, San Diego, La Jolla, California 92093, United States; 2 Department of Chemistry and Biochemistry, University of California, San Diego, La Jolla, California 92093, United States; 3 Department of Science and Conservation, San Diego Botanic Garden, Encinitas, California 92024, United States; 4 Department of Chemistry, Faculty of Science and Technology, Keio University, 3-14-1 Hiyoshi, Kohoku-ku, Yokohama, Kanagawa 223-8522, Japan; 5 Dipartimento di Farmacia, Università degli Studi di Napoli Federico II, via Domenico Montesano 49, Napoli 80131, Italy; 6 Institute of Chemistry, São Paulo State University (UNESP), Araraquara 14800-060, Brazil; 7 Skaggs School of Pharmacy and Pharmaceutical Sciences, University of California, San Diego, La Jolla, California 92093, United States; 8 Institute for Pharmaceutical Biology and Biotechnology, Department of Pharmacy, Philipps-University Marburg, Robert-Koch-Straße 4, 35037 Marburg, Germany; 9 Interfaculty Institute of Microbiology and Infection Medicine, University of Tuebingen, Tuebingen 72076, Germany; 10 Department of Biochemistry, University of California, Riverside, Riverside, California 92521-9800, United States; 11 College of Pharmacy, Dongguk University, Goyang 10326, South Korea; 12 Sorbonne Université, CNRS, Laboratoire d’Océanographie de Villefranche (UMR 7093), 06230 Villefranche-sur-Mer, France; 13 Department of Pharmaceutical Sciences, School of Pharmacy, University of Puerto Rico - Medical Sciences Campus, San Juan 00935, Puerto Rico

## Abstract

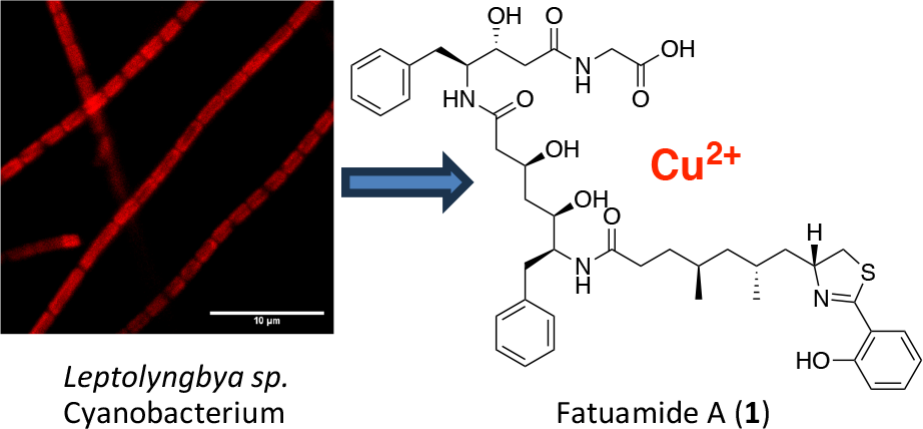

A structurally novel metabolite, fatuamide A (**1**), was discovered from a laboratory cultured strain of the marine cyanobacterium *Leptolyngbya* sp., collected from Faga’itua Bay, American Samoa. A bioassay-guided approach using NCI-H460 human lung cancer cells directed the isolation of fatuamide A, which was obtained from the most cytotoxic fraction. The planar structure of fatuamide A was elucidated by integrated NMR and MS/MS analysis, and a combination of bioinformatic and computational approaches was used to deduce the absolute configuration at its eight stereocenters. A putative hybrid PKS/NRPS biosynthetic gene cluster responsible for fatuamide A production was identified from the sequenced genomic DNA of the cultured cyanobacterium. The biosynthetic gene cluster possessed elements that suggested fatuamide A binds metals, and this metallophore property was demonstrated by native metabolomics and indicated a preference for binding copper. The producing strain was found to be highly resistant to toxicity from elevated copper concentrations in culture media.

Marine cyanobacteria continue to be an extraordinarily rich source of diverse natural products, many of which have potent biological activities with promising therapeutic potential.^1^ Genomic analyses have revealed that these photosynthetic bacteria possess the capacity to produce many more natural products than have currently been isolated and characterized.^2^ For example, while members of the genus *Leptolyngbya* have been a rich source of structurally diverse novel secondary metabolites, including coibamide A, honaucin A, the crossbyanols, and molassamide,^3^ DNA sequencing analyses have revealed many unknown compounds encoded in their genomes which await future discovery.^4^ The secondary metabolites that have been isolated from *Leptolyngbya* spp. have been shown to possess a variety of biological activities including antibacterial, cytotoxicity to cancer cells, and anti-inflammatory effects.^3^

American Samoa is a geographically remote site which has been rarely explored for its natural product-rich biota. In 2014, we made a collection of a shallow subtidal marine *Leptolyngbya* sp. from Faga’itua Bay and subsequently adapted it to laboratory culture.^5^ This allowed us to obtain larger quantities of biomass than was available from field collection, and thus embark on an examination of its bioactive metabolites, initially focusing on those with potential anticancer effects, as natural product-derived or inspired compounds make up almost 65% of the current clinical anticancer drugs.^6^ For instance, several antibody-drug onjugate (ADC) anticancer drugs use a warhead that is modified from the antitubulin agent dolastatin 10, a metabolite isolated from the cyanobacterium *Symploca* sp.^7^ Thus, a bioassay-guided approach using NCI-H460 human lung cancer cells directed the isolation of fatuamide A, which was obtained from the most cytotoxic fraction (Figure S1, Supporting Information).

Among the diverse classes of natural products produced by cyanobacteria are “metallophores”, compounds that are commonly used by an organism to acquire metals from its surrounding environment. Such metal–organic compound complexes can have unique applications in medicine. For example, metallophores have been used to chelate and thus remove toxic metals from the body, such as aluminum and vanadium. Siderophores (metallophores that bind iron) have also been used for the treatment of different diseases that result in an excess of iron,^8^ including inhibitors of ferroptosis.^9^ Some metallophores can bind metals such as copper and zinc with higher affinity than iron. For example, methanobactin is highly selective for binding Cu^2+^ due to the number and orientation of its heterocyclic rings.^10^ There is a growing recognition that Alzheimer’s disease (AD) involves the precipitation of amyloid-beta by zinc and its radicalization by copper, and both metals are markedly enriched in AD-associated plaques.^11^ Metallophores can also be utilized as drug delivery systems, such as in a siderophore-antibiotic conjugate.^12^ From another perspective, production of organic molecules that bind to potentially toxic metals, such as copper, cadmium or lead, can be positively adaptive to the producer organism for their detoxification capacity.^13^

Most of the reported cyanobacterial siderophores have been obtained from freshwater sources, and include 19 with hydroxamate functionalities, six with catecholate functionalities, and another five that are uncategorized.^14^ The three best characterized cyanobacterial siderophores are schizokinen, the synechobactins, and anachelin.^14^ Schizokinen, a hydroxamate siderophore, was first reported from the freshwater cyanobacterium *Anabaena* sp.^15^ Synechobactins A-C are amphiphilic hydroxamate siderophores that were obtained from a marine *Synechococcus sp*.^16^ Anachelin is a catechol siderophore that was obtained from the freshwater species *Anabaena cylindrica*.^17^ In general, metallophore characterization from cyanobacteria, especially marine species, has been understudied to date. However, we recently reported leptochelins A-C, complex phenolate-type metallophores from various collections of the marine cyanobacterial genus *Leptothoe*.^18^ Herein we report the unique structure, putative biosynthesis and several biological properties of another new phenolate-type metallophore, fatuamide A (**1**), from a *Leptolyngbya* sp. of marine cyanobacteria.
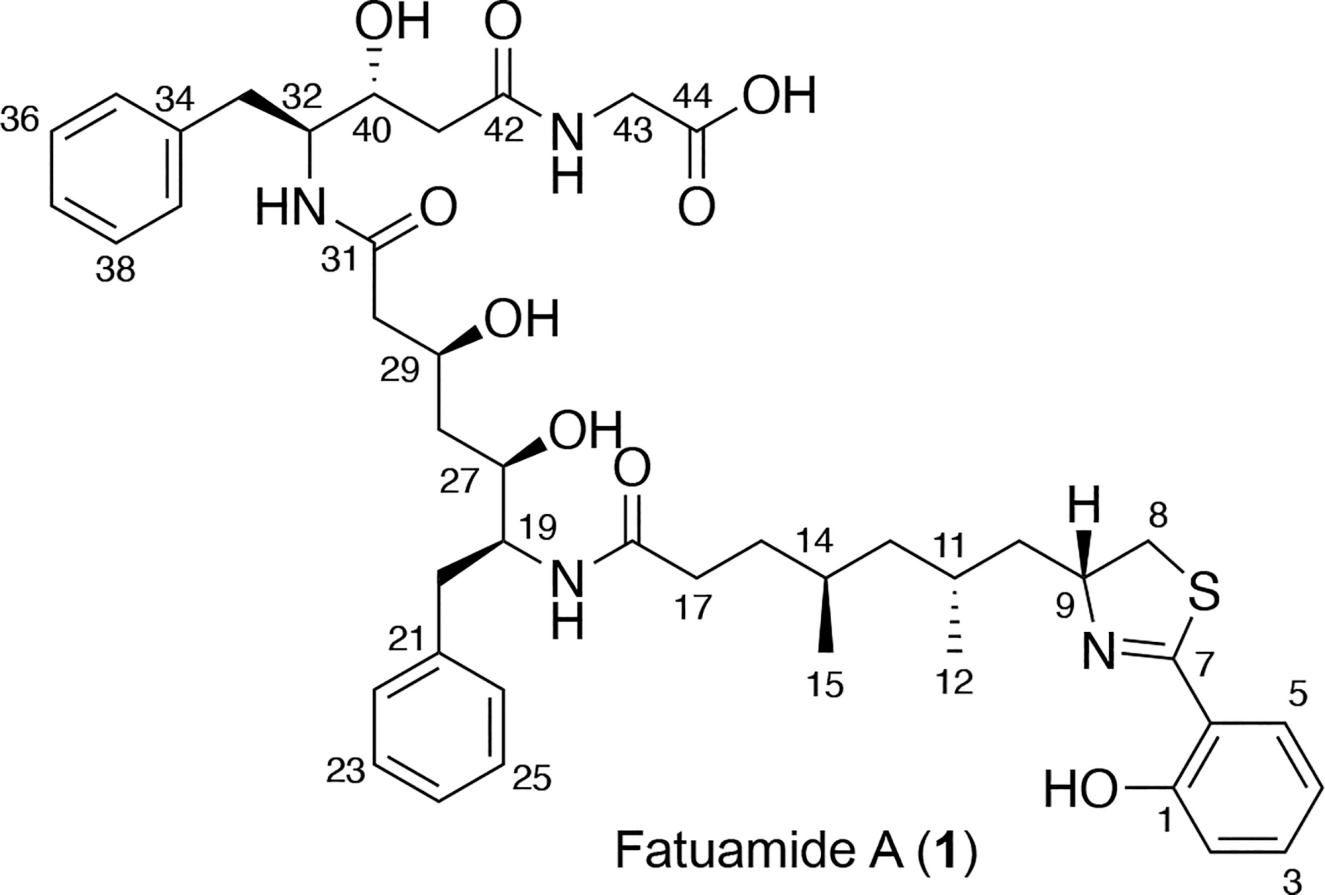


## Results and Discussion

### Collection and Culture

**Figure 1:**
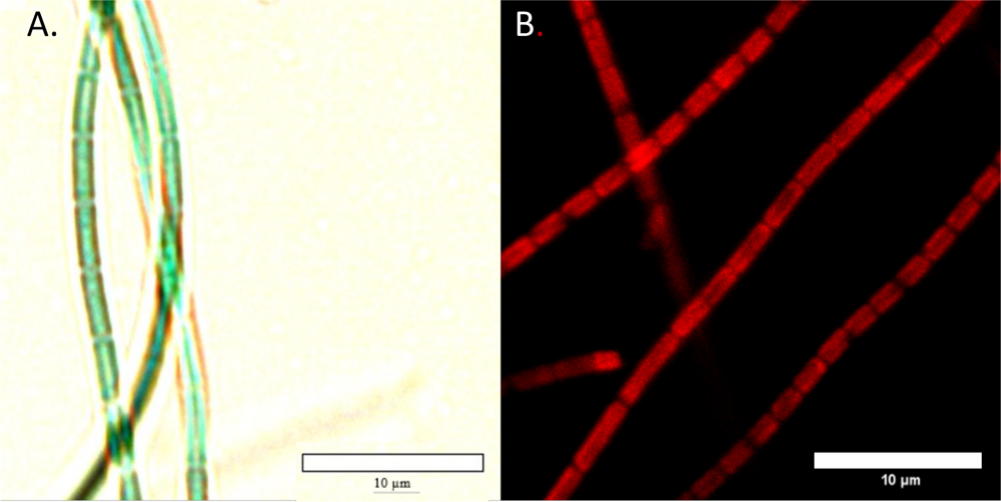
Photomicrographs of cultured *Leptolyngbya* sp. ASX22JUL14-2 were obtained using (A) a Life Technologies EVOS XL Core microscope with an Olympus 100X oil immersion objective and (B) a Leica CTR6500 microscope with a 40X water immersion objective using a 552 nm laser and filters to capture the signal between 550 and 750 nm with a central detection wavelength around 620 nm.

The cyanobacterial sample ASX22JUL14-2 was collected from Faga’itua Bay, American Samoa, isolated as a pure unicyanobacterial culture strain, and then maintained in SWBG11 media. The sample was identified as a *Leptolyngbya* sp. on the basis of morphology and a previously reported phylogenetic analysis (Figure 1).^4^ Species of *Leptolyngbya* are reported to grow slowly,^3^ and this was observed for this strain as scale-up cultures in aerated glass carboys containing 9–13 L of SWBG11 media took 3–5 months to provide sufficient biomass for the detailed chemical investigations described below.

### Extraction and Bioassay Guided Isolation

The fresh cyanobacterial biomass obtained from several culture carboys was exhaustively extracted with CH_2_Cl_2_:MeOH (2:1), and the extract was fractionated into nine subfractions using normal phase silica gel vacuum liquid chromatography (VLC). The material eluting in the most polar fraction (fraction I, eluting with 100% MeOH) from the VLC column inhibited the *in vitro* growth of NCI-H460 cells by 75% at 10 μg/mL (Figure S1) and showed no anti-inflammatory effect in mouse RAW cells at 30 μg/mL (Figure S2). An LC-MS/MS based molecular network (Figure S3) was constructed from this extract and its fractions to annotate the compounds present. One cluster in the network was almost entirely composed of compounds present in the cytotoxic fraction I. A prominent member of this cluster possessed an *m*/*z* of 819 and was subsequently targeted for further purification. This compound, given the name fatuamide A (**1**), was purified from fraction I using reversed-phase flash chromatography on a CombiFlash system to yield *ca*. 1 mg of pure compound.

### Structure Elucidation

Fatuamide A (**1**) was isolated as a white amorphous solid with a molecular formula of C_44_H_58_N_4_O_9_S as deduced from HR-ESI-TOFMS {[M + H]^+^ ion at *m*/*z* 819.4000; 0.0003 Da error; 18 double bond equivalents} (Figure S4). Corroboration of this molecular formula, including the presence of one sulfur atom, was obtained by SIRIUS 4.0 employing its isotope pattern analysis tool (Figure S5).^19^

The NMR-based artificial intelligence (AI) tools “SMART” (http://smart.ucsd.edu/classic) and “DeepSAT” (https://deepsat.ucsd.edu) were used to guide dereplication and structure elucidation efforts of fatuamide A (**1**).^20,21^ The class prediction as an “oligopeptide” and “top 10” results (the compounds most similar to fatuamide A) on the basis of their HSQC spectra are shown in Figure S7. Motifs highlighted in the top hit compounds include phenol moieties and phenylalanine residues; these are consistent with the deduced structure of fatuamide A as described below.

From ^13^C NMR analysis, there were five putative ester/amide-type carbonyls between δ_C_ 171–176, and two monosubstituted and one disubstituted phenyl rings with shifts between δ_C_ 117–160, therefore accounting for 17 degrees of unsaturation and indicating the presence of one additional ring. The ^1^H NMR spectrum had resonances for a methylene group at δ_H_ 3.02 and 3.52 with an associated carbon at δ_C_ 37.58 (C-8) by HSQC. This was adjacent to a deshielded proton at δ_H_ 4.76 with an associated carbon at δ_C_ 75.5 (C-9); in combination with HMBC correlations from H-9 to the deshielded resonance at δ_C_ 171.9 (C-7), as well as consideration of the atom composition of fatuamide A, these data helped to define a thiazoline ring as the final degree of unsaturation.

**Figure 2:**
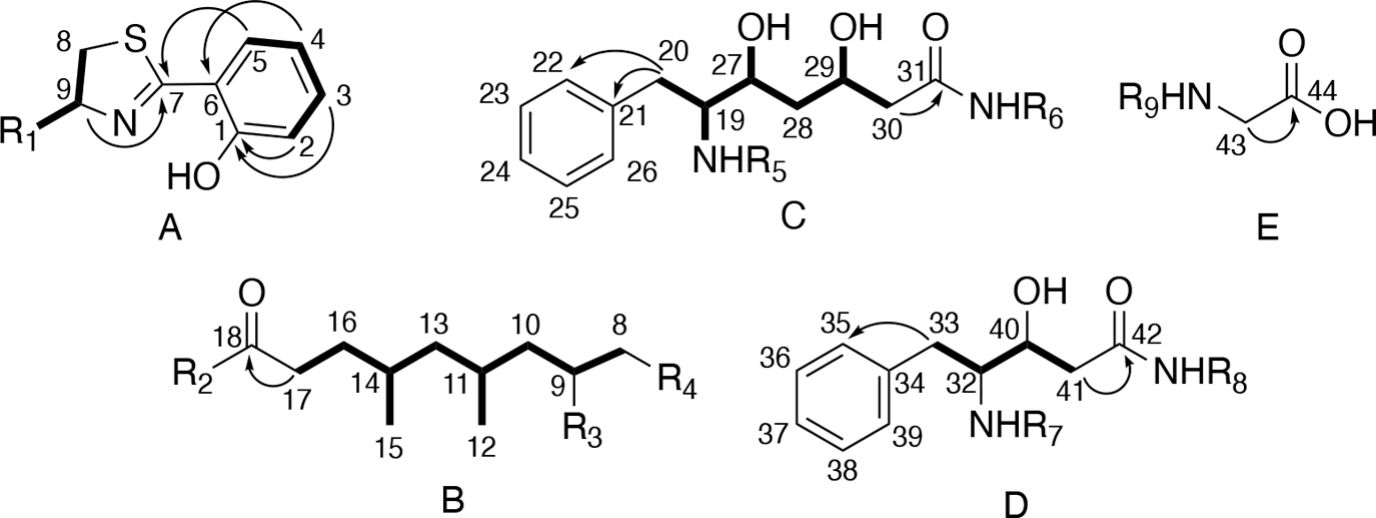
Partial structures A–E determined by NMR for fatuamide A (**1**) (arrows depict HMBC correlations, while bold bonds indicate COSY connections).

**Figure 3:**
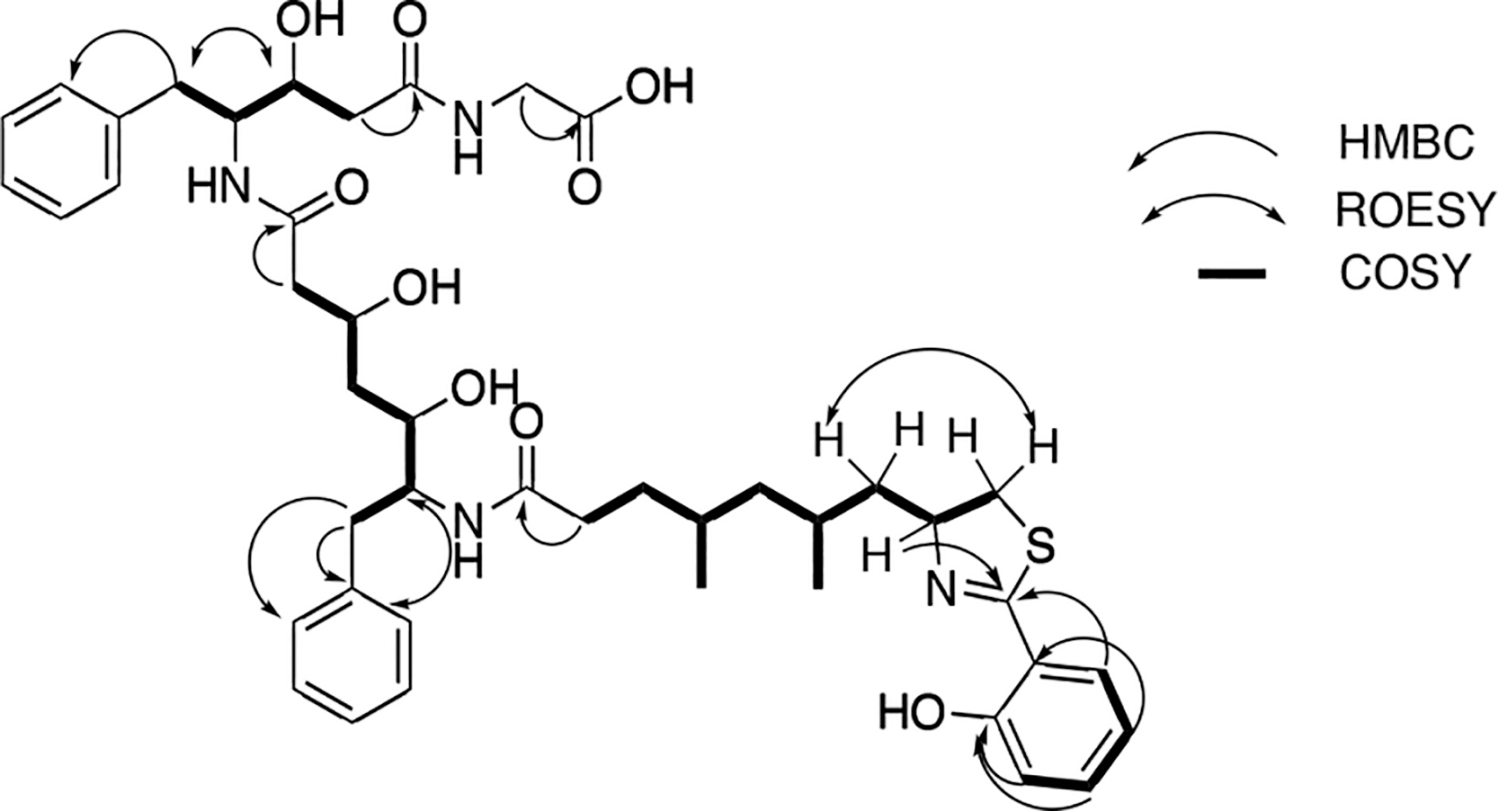
COSY (bold bonds) and selected ROESY and HMBC correlations of fatuamide A (**1**).

The planar structure of fatuamide A (**1**) was determined from an integrated use of NMR data (COSY, HSQC, H2BC, HSQC, TOCSY, and HMBC data; Table 1 and Figures 2 and 3), mass spectrometry analysis, and a detailed analysis of its putative biosynthetic gene cluster (BGC). The partial structures A–E were determined through COSY, HSQC, and HMBC (Figure 2). Overlapped signals were distinguished using H2BC and band selective HSQC experiments.

**Table 1:** ^1^H and ^13^C NMR Spectroscopic Data of Fatuamide A (**1**) in Methanol-*d*_4_ (^1^H 500 MHz, ^13^C 125 MHz)

Position	δ_C_, type	δ_H_ (*J* in Hz)	Selected HMBC (H → C)
1	160.2, C	–	
2	117.8, CH	6.93, m	1, 2, 4, 6
3	134.0, CH	7.35, ddd (8.3, 7.4, 1.6)	1, 5
4	120.0, CH	6.89, ddd (7.8, 7.4, 1.0)	2, 6
5	131.6, CH	7.42, dd (7.9, 1.5)	1, 3, 7
6	117.6, C	–	
7	171.9, C	–	
8a	37.58, CH_2_	3.02, dd (11.0, 8.3)	9, 10
8b		3.52, dd (11.0, 8.2)	10
9	75.5, CH	4.76, dddd (8.3, 8.2, 8.0, 6.7)	7
10a	44.3, CH_2_	1.59, ddd (13.2, 6.7, 6.7)	9, 10, 11, 12, 13
10b		1.69, ddd (13.2, 6.7, 6.7)	8, 9, 10, 11, 12, 13
11	29.3, CH	1.78, ddddq (9.1, 7.0, 6.5, 4.5, 6.7)	
12	20.3, CH_3_	0.94, d (6.6)	10, 11, 13
13a	45.1, CH_2_	1.11, ddd (13.5, 9.3, 4.5)	14, 17, 18
13b		1.25, m	
14	31.1, CH	1.47, m	
15	19.4, CH_3_	0.84, d (6.3)	13, 14, 16, 17
16	35.0, CH_2_	2.07, m	
17a	35.0, CH_2_	1.20, m	14
17b		1.41, m	
18	176.2, C	–	
19	56.9, CH	4.01, ddt (15.9, 6.3, 3.5)	
20a	36.91, CH_2_	2.61, m	21, 22, 19
20b		3.05, m	
21	139.9, C	–	
22	130.4, CH	7.22^a^, m	
23	129.3, CH	7.22^a^, m	
24	127.3, CH	7.15^a^, m	22
25	129.3, CH	7.22^a^, m	
26	130.4, CH	7.22^a^, m	
27	71.3, CH	3.75, m	
28a	41.8, CH_2_	1.44, m	
28b		1.56, m	
29	66.7, CH	4.13, m	
30a	45.3, CH_2_	2.18, dd (14.2,4.2)	31
30b		2.26, dd (14.2,8.5)	29, 31
31	173.9, C	–	
32	56.5, CH	4.11, m	
33a	37.28, CH_2_	3.13, dd (13.9, 3.6)	
33b		2.65, m	35, 32
34	140.3, C	–	
35	130.4, CH	7.22^a^, m	
36	129.2, CH	7.22^a^, m	
37	127.2, CH	7.15^a^, m	36
38	129.2, CH	7.22^a^, m	36
39	130.4, CH	7.22^a^, m	
40	71.9, CH	3.95, m	
41a	41.4, CH_2_	2.40, dd (14.7, 9.2)	42
41b		2.58, m	42
42	174.5, C	–	
43a	42.2, CH_2_	3.90, s	
43b		3.94, d (4.8)	44
44	173.5, C	–	

a Signals may be interchanged due to overlap.

From the aromatic region of the ^1^H NMR, it was evident that there were three different phenyl rings, two of which were subsequently shown to be derived from phenylalanine and one from salicylic acid. Four aromatic protons (δ_H_ 6.89, 6.93, 7.35, and 7.42) were sequentially connected by COSY correlations, and by HMBC these were connected to a phenolic carbon C-1 at δ_C_ 160.2 and a carbon substituted C-6 at δ_C_ 117.6. Further, the C-5 aromatic proton was connected by HMBC to a deshielded carbon at δ_C_ 171.9, defining substructure A as deriving from a salicylic acid moiety (Figure 2). This same deshielded carbon at δ_C_ 171.9 was also a component of the thiazoline ring as defined above, thus joining these two moieties to complete fragment A. A salicylate-derived subunit connected to a thiazoline or oxazoline ring is a moiety seen in several other siderophores and ionophores, such as in leptochelins A-C, pyochelin, amychelin, and yersiniabactin;^18,22−24^ the thiazoline-salicylate unit in the latter compound has similar chemical shifts to those observed for fatuamide A (Figure S8).^22^

**Figure 4:**
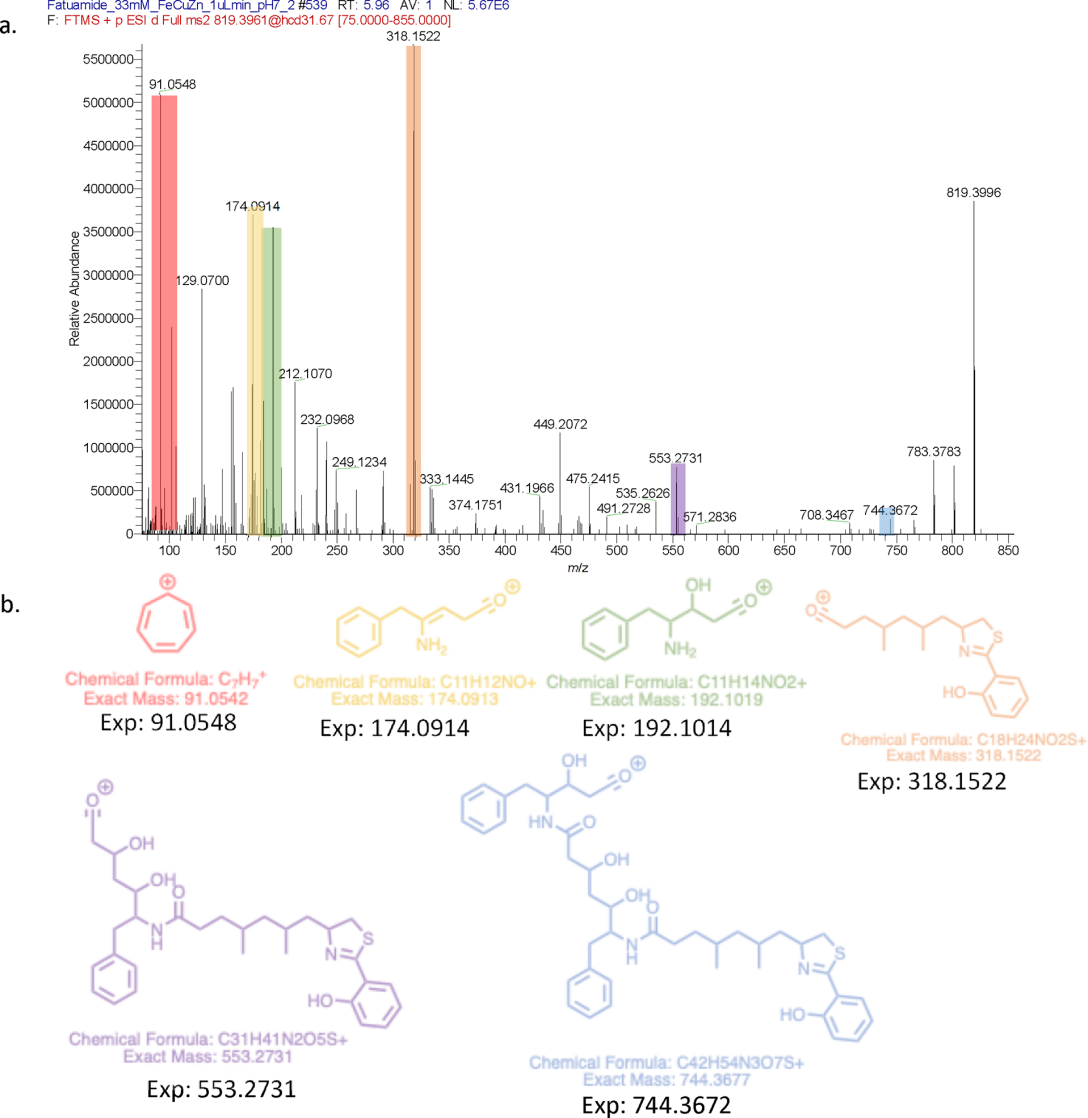
Fatuamide A (**1**) fragments observed by ESI MS. Fragments are highlighted (b) and color coded in the MS/MS spectrum (a), and the corresponding fragments are illustrated.

Fragment B had two three-proton methyl doublets (C-12 and C-15) at δ_H_ 0.94 and 0.84 that were connected to carbon chain C-8-C-17. These methyl groups were attached at C-11 and C-14 through COSY correlations, resulting in a 1,3-dimethyl group arrangement. By COSY, the C-10 methylene group was adjacent to the deshielded proton at C-9, assigned above as a component of the thiazoline ring in fragment A. The H-17 methylene protons showed HMBC correlation to an amide-type carbonyl group at C-18 (δ_C_ 176.2), completing partial structure B. Fragment C consisted of a carbon chain connected by sequential COSY correlations between protons 20 → 19 → 27 → 28 → 29 → 30. The chemical shift at C-19 (δ_C_ 56.9) was indicative of its attachment to a nitrogen atom while those of C-27 (δ_C_ 71.3, δ_H_ 3.7) and C-29 (δ_C_ 66.7, δ_H_ 4.13) were consistent with single bond attachments to oxygen. The H-20 methylene was correlated by HMBC to aromatic carbons at C-21 and C-22/26, therefore placing an aromatic ring at this terminus of fragment C. The other terminus was correlated by HMBC to another amide-type carbonyl at δ_C_ 173.9, completing partial structure C. Fragment D was comprised of a carbon chain involving sequential COSY correlations between C-33 → C-32 → C-40 → C-41. The methine proton at C-40 had a chemical shift of δ_H_ 3.95 (δ_C_ 71.9), indicating the attachment of an oxygen atom at that position, whereas C-32 had a shift of δ_c_ 56.5, indicating an attachment to a nitrogen atom. The H-33 protons had HMBC correlations to aromatic carbon atom C-35/39, again placing an aromatic ring at one terminus of this partial structure. The H-41 methylene protons showed an HMBC correlation to an amide-type carbonyl at C-42 (δ_C_ 174.5), thus completing fragment D. Finally, fragment E was comprised of a methylene group at C-43, the carbon shift of which (δ_c_ 42.2) indicated that it was attached to a nitrogen atom. The H-43 protons were correlated by HMBC to a carbonyl at δ_c_ 173.5 (C-44), completing partial structure E. Partial structures A and B were able to be combined due to overlapping assignments of C-8 and C-9 in both fragments. However, NMR correlations were lacking to connect the remaining fragments, and therefore mass spectrometry was used to establish these connections (Figure 4).

The MS/MS spectrum (Figure 4) showed a fragment at *m*/*z* 318.1522, corresponding to cleavage of the C-18 amide bond to release combined fragment AB. Similarly, the *m*/*z* 553.27 fragment corresponds to the cleavage of the C-31 amide bond, thus establishing the connection of fragment C with combined fragment AB. MS/MS analysis of fragment *m*/*z* 553.27 yielded fragment *m*/*z* 434.24, a cleavage also seen in yersiniabactin (Figure S9).^18^ This MS/MS analysis also yielded fragment *m*/*z* 318.15, providing further support for the connection of partial structure C to structure AB. The fragment at *m*/*z* 744.37 corresponds to cleavage of the C-42 amide bond, connecting partial structure D to combined fragment ABC. The mass difference between the molecular weight and combined fragments ABCD, or 818 – 744 = 74 Da, accords to the remaining unassigned atoms, C_2_H_4_NO_2_, matching that of partial structure E, a terminal glycine residue, and thus completing the structure of fatuamide A as a linear arrangement of partial structures ABCDE.

### Putative Biosynthetic Gene Cluster

**Figure 5:**
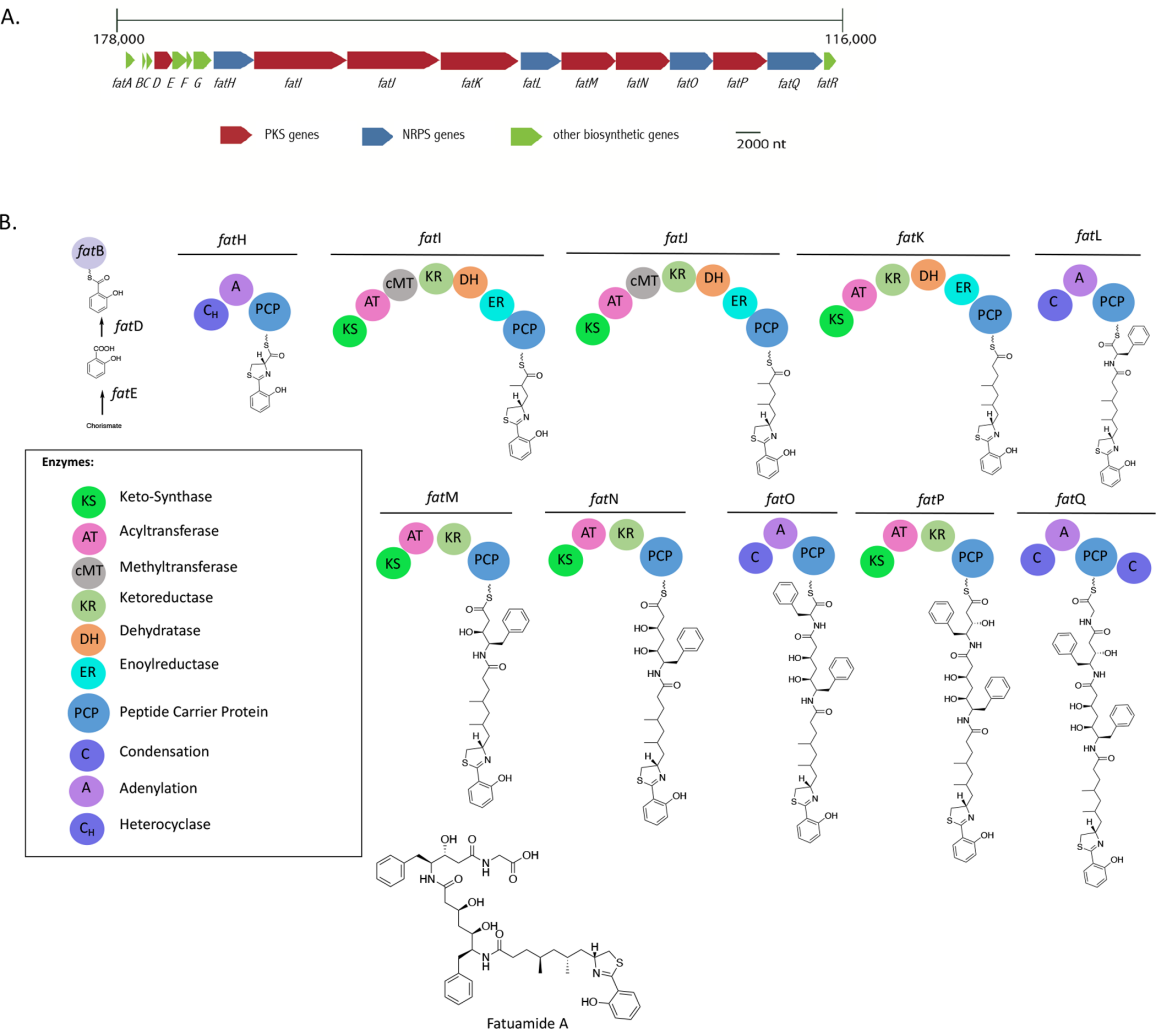
(A) Putative fatuamide A (**1**) biosynthetic gene cluster containing PKS/NRPS genes detected using AntiSMASH.^25^ (B) Proposed biosynthesis of fatuamide A (**1**). Configurations of stereocenters deduced by bioinformatics are depicted in the assembly process.

The genomic DNA of the *Leptolyngbya sp*. culture that produced **1** had previously been sequenced and assembled.^4^ The antiSMASH^25^ output of the genome showed two BGCs that possessed mixed PKS/NRPS biosynthetic features. The putative fatuamide A BGC, comprised of 60,556 nucleotides, was identified through different genetic features that were indicative of its unique structure as deduced from the NMR data (Figure 5). The BGC has a salicylate synthase, two adenylation domains that are specific for phenylalanine incorporation, several elements of PKS extension, and cMT domains. Intriguingly, the *fat* BGC is extended by several additional NRPS and PKS modules that conceptually lengthen fatuamide A by four additional amino acids and one PKS unit to form partially characterized fatuamide B (Figures S10, S11, Table S1). We hypothesize that these latter structural features are either not incorporated as a result of condensation domain-based termination after the final amino acid incorporation into fatuamide A (e.g., glycine, see discussion below), or are subsequently removed at some stage of processing or cellular export to produce fatuamide A (**1**) (Figure S10 and Table S1).

The biosynthetic pathway of fatuamide A is predicted to begin with FatE, a salicylate synthase homologue that likely catalyzes the conversion of isochorismate into salicylate, as seen in other natural products such as attinimicin and amychelin.^26,22^ Salicylate is next attached to FatB, an acyl carrier protein, by FatD, a member of the benzoate-CoA ligase family. FatH through FatQ are made up of a mixture of NRPS and PKS enzymes (Table 2) that show complete coherence between predicted function and the NMR- and MS-based structure of fatuamide A. FatR is a TauD/TfdA family dioxygenase; it is predicted to β-hydroxylate aspartic acid, a residue that is found in fatuamide B, the predicted longer homologue of fatuamide A that was observed in trace quantities (Figures S10, S11 and Table S1). BLAST analysis of FatA at the beginning of the pathway indicates that it is a thioesterase with similarity to the gramicidin dehydrogenase LgrE (67% identity), gramicidin S biosynthesis protein GrsT (63% identity) and microcystin thioesterase MycT (63% identity). We speculate that FatA is an editing-type thioesterase involved in maintaining the fidelity of the fatuamide A pathway, as discussed further below. Additionally, the *fat* BGC contains a Major Facilitator Superfamily (MFS) transporter (ctg11_67), ABC transporter binding protein (ctg11_66), and a Ton-B dependent receptor (ctg11_64) (Table S3). *Anabaena* sp. also contains an MFS protein that is used for the secretion of the siderophore schizokinen.^13^ Siderophores with bound iron are imported by Ton-B dependent transporters and ABC transporters^13^ and the presence of these various accessory genes in the *fat* BGC supports the prediction that fatuamide A (**1**) is a metal-binding and possibly transporting siderophore, as discussed further below.

**Table 2:** Predicted Functions of Encoded Proteins in the Fatuamide A Biosynthetic Gene Cluster

Protein	Size (nt)	Proposed Function (AntiSMASH)	Similar Sequence	Identity	Coverage	E-value	Accession number
FatA	768	thioesterase	thioesterase, Pleurocapsales cyanobacterium LEGE 06147	73.52%	99.00%	5.00 × 10^–134^	MBE9170818.1
FatB	249		acyl carrier protein, *Leptolyngbya sp*. SIO1D8	78%	92%	5.00 × 10^–132^	NER81856.1
FatC	459	4′-phosphopantetheinyl transferase superfamily	4′-phosphopantetheinyl transferase superfamily protein, *Leptolyngbya sp*. SIO1D8	70%	97%		NER81857.1
FatD	1539	AMP-dependent synthetase and ligase	benzoate-CoA ligase family protein, *Leptolyngbya sp*. SIO1D8	87%	100%	0	NER81858.1
							NER81859.1
FatE	1323	isochorismate synthase	salicylate synthase, *Leptolyngbya sp*. SIO1D8	88%	96%	0	HAZ44672.1
FatF	486		TPA: holo-[acyl-carrier-protein] synthase, Cyanobacteria bacterium UBA 11371	50%	95%	2 × 10^–49^	
			3-oxoacyl-[acyl-carrier-protein] synthase, KAS III, uncultured				
FatG	1524	3-oxoacyl-(acyl carrier protein) synthase	*Coleofasciculus sp*.	69.35%	99.00%	0	CAA9297985.1
FatH	3474	C (heterocyclization), A-Cys, P	BarG, *Anabaena cylindrica* PCC 7122	65.00%	100.00%	0	AP018166.1
FatI	7944	KS, AT, cMT, KR, DH, ER, PCP	cis-AT_polyketide_synthase, *Nostoc* sp. *Peltigera membranacea* cyanoboint	42%	99.30%	0	GQ979609.2
FatJ	7944	KS, AT, cMT, KR, DH, ER, PCP	cis-AT_polyketide_synthase, *Nostoc* sp. Peltigera membranacea cyanoboint	43%	98.00%	0	GQ979609.2
FatK	6654	KS, AT, KR, DH, ER, PCP	cis-AT_polyketide_synthase, *Nostoc sp. Peltigera membranacea* cyanoboint	52%	99.00%	0	GQ979609.2
FatL	3456	C, A-Phe, P	nonribosomal protein synthetase, Anabaena cylindrica PCC7122	56%	96.40%	0	AP018166.1
FatM	4650	KS, AT, KR, PCP	type I polyketide synthase, *Fischerella* sp. PCC 9431	55.00%	99.40%	0	NZ_KE650771.1
FatN	4638	KS, AT, KR, PCP	type I polyketide synthase, *Fischerella* sp. PCC 9431	56%	98.40%	0	NZ_KE650771.1
FatO	3678	C, A-Phe, P	nonribosomal protein synthetase, Anabaena cylindrica PCC7122	53%	96%	0	AP018166.1
FatP	4644	KS, AT, KR, PCP	type I polyketide synthase, *Fischerella* sp. PCC 9431	59%	98.80%	0	NZ_KE650771.1
FatQ	4755	C, A-gly, P, C	NcpA, Nostoc sp. ATCC 53789	55%	96.80%	0	AY167420.1
FatR	1005	Dioxygenase TauD/TfdA	TauD/TfdA family dioxygenase, *Leptolyngbya* SIOiD8	81.68%	99.00%	0	NER81770.1

The biosynthesis of the siderophore amychelin by the rare actinomycete *Amycolatopsis* sp. AA4 involves the conversion of isochorismate into salicylate by a salicylate synthase. Salicylate is then incorporated into the biosynthetic pathway by a hydroxybenzoyl AMP ligase.^23^ FatE is a salicylate synthetase and FatD shows 44.9% identity to a 4-hydroxybenzoate ligase obtained from a *Candidatus Rokubacteria* bacterium. The thiazole-salicylate unit in yersinabactin is produced by a NRPS module that combines an activated salicylate residue on an acyl carrier protein with a cysteine unit; the latter is subsequently cyclized and dehydrated to a thiazoline ring.^27−29^ Similarly, FatH is an NRPS module that is predicted to activate and then incorporate cysteine which is subsequently cyclized and dehydrated to form the condensed thiazoline-salicylate unit. This is followed by two PKS extensions (FatI and FatJ) in which the sequentially produced β-keto functionalities are first α-methylated by cMTs and then fully reduced. Next, FatK catalyzes a third PKS extension that is fully reduced. Subsequently, the FatL NRPS catalyzes the incorporation of a phenylalanine residue, and this is followed by two PKS extensions (catalyzed by FatM and FatN) with reduction of the keto groups. Ensuing, a second phenylalanine residue is added by the FatO NRPS, and this also undergoes a PKS extension; the resulting carbonyl is reduced by FatP to a secondary alcohol. Lastly, a glycine residue is added by a final NRPS module, FatQ.

Due to its location in the BGC, FatA is conceivably a type II thioesterase (TE).^30^ Type II thioesterases have several different functions in secondary metabolite pathways.^30^ Most biosynthetic pathways have an in-line Type I thioesterase at the terminus which is responsible for product release, and an additional TEII that is used to edit or reprime units.^30,31^ However, there are several ionophores such as nanchangmycin, monensin, and nigericin/abierixin that use a TEII to catalyze final product release.^32^ Amino acid alignment of the thioesterase from fatuamide A with those of nanchagmycin, monesin, nigericin and yersiniabactin showed 18%, 41%, 18% and 29% pairwise identity, respectively. Alternatively, the release of fatuamide A could be catalyzed by the putative terminating condensation unit (the second C domain in FatQ) that is present after the glycine-encoding NRPS in the BGC. Condensation domain catalyzed release has been observed or predicted for FK520, apratoxin A, and aeruginoside.^33^ Alignment of the amino acids from the additional condensation domain in the fatuamide A BGC with those listed above gave 29%, 42% and 41% pairwise identity, respectively. A phylogeny of these condensation domains was constructed and the second condensation unit of FatQ falls within the same clade as the terminating condensation unit of aeruginoside (Figure S12).

### Configuration of Fatuamide A (**1**)

Resulting from the small amount of isolated fatuamide A (**1**), it was not possible to establish the configuration of its eight stereocenters by reaction-based analytical techniques; therefore, a combination of bioinformatic and computational approaches was used. The first stereocenter produced during the biosynthetic process is C-9, a thiazoline ring derived from cysteine and salicylic acid; due to the lack of an epimerase domain in the NRPS module responsible for its incorporation, the configuration is proposed as deriving from the natural l-stereoisomer (*R* configuration). There are two stereocenters derived from the incorporation of two phenylalanine residues (C-19 and C-32); based on analysis of the BGC, these are also predicted to incorporate l-phenylalanine without epimerization, and thus to both be of *S* configuration.

The configurations of the three secondary alcohol centers at C-27, C-29, and C-40 were annotated by antiSMASH to have l-configuration (resulting in 27*R*, 29*S*, 40*R*).^24^ This result is deduced from the KR domains responsible for their formation.^34^ The three KR domains were aligned with other cyanobacterial domains to gain further insight (Figure S13). In all three cases, the KR domains lack the characteristic LDD motif, a defining feature of B-type KR domains. It has been shown that the second of the two D residues of this sequence is highly conserved in B-type KRs, but this residue was absent from all three fatuamide A KR domains, suggesting that they were A-type KRs. However, all three of these hydroxy-producing KR domains also lack the highly conserved W residue that is consistently present in A-type KR domains. Therefore, it appears that fatuamide A has a mix of A-type and B-type KR domains because they lack both the pivotal W residue and the LDD motif. In a study that analyzed KR domains from different organisms, all those that contained a second D in the LDD loop produced a d-product.^35^ Therefore, because fatuamide A lacks the diagnostic D of the LDD loop for the three hydroxy-producing KRs, we conclude that these KR domains form l-products in each case, consistent with the AntiSMASH results.^24^

The configuration at C-11 and C-14 were challenging to characterize using bioinformatics. The KS domains in FatI and FatJ were analyzed using NaPDOS2 to evaluate if there were any correlations between their sequences and the configuration of the resulting stereogenic centers; however, this analysis was inconclusive. The cMT in *cis*-AT pathways is not well studied in terms of the absolute configuration of its products.^36^ It was found that only 1.7% of *cis*-AT modules contain cMT domains.^37^ Nevertheless, homology was found in the sequences of cMTs that produce similar products, such as in yersiniabactin and pyochelin.^38^ There have also been some recent studies on the configurations of products of cMT enzymes present in *trans*-AT polyketide synthases.^39^ To explore whether particular sequences of cMTs are associated with the configuration of the resulting stereocenters bearing methyl groups, a dendrogram was created using the neighbor-joining tree building method with 1,000 bootstraps on Geneious. The input data were sequences of cMTs from cyanobacterial compounds with known configurations at methyl branch points. Unfortunately, there was no distinguishable stereochemical outcome deduced from the clusters established in the dendrogram (Figure S14). Amino acid sequence alignments were evaluated for the available cyanobacterial ER domains that produce compounds with known configurations for methines bearing methyl groups with fully reduced adjacent methylene groups at the biosynthetically upstream position (i.e., the methyl branch configuration is set by the reductive action of the ER domain; Figure S15). Lack of a Y residue at the position for stereocontrol^40^ tentatively suggested that both C-11 and C-14 of fatuamide A might be of *R* configuration.

The relative configuration of the 1,3-dimethyl arrangement was also explored considering the chemical shift difference of the geminal methylene protons.^41^ The chemical shift difference of the geminal methylene protons (H-13a and H-13b) of fatuamide A (**1**) is 0.14 ppm. Values deviating less than 0.1 ppm have been shown to correlate with *anti*-relationship of the methyl groups, whereas those more than 0.4 ppm correlate with a *syn*-arrangement. Values intermediate between 0.1 and 0.4 ppm could be either *syn*- or *anti*-, and require further analysis and comparison to literature data.^41^ Unfortunately, this analysis as shown in Figure S16 was inconclusive.^42,43^

**Figure 6:**
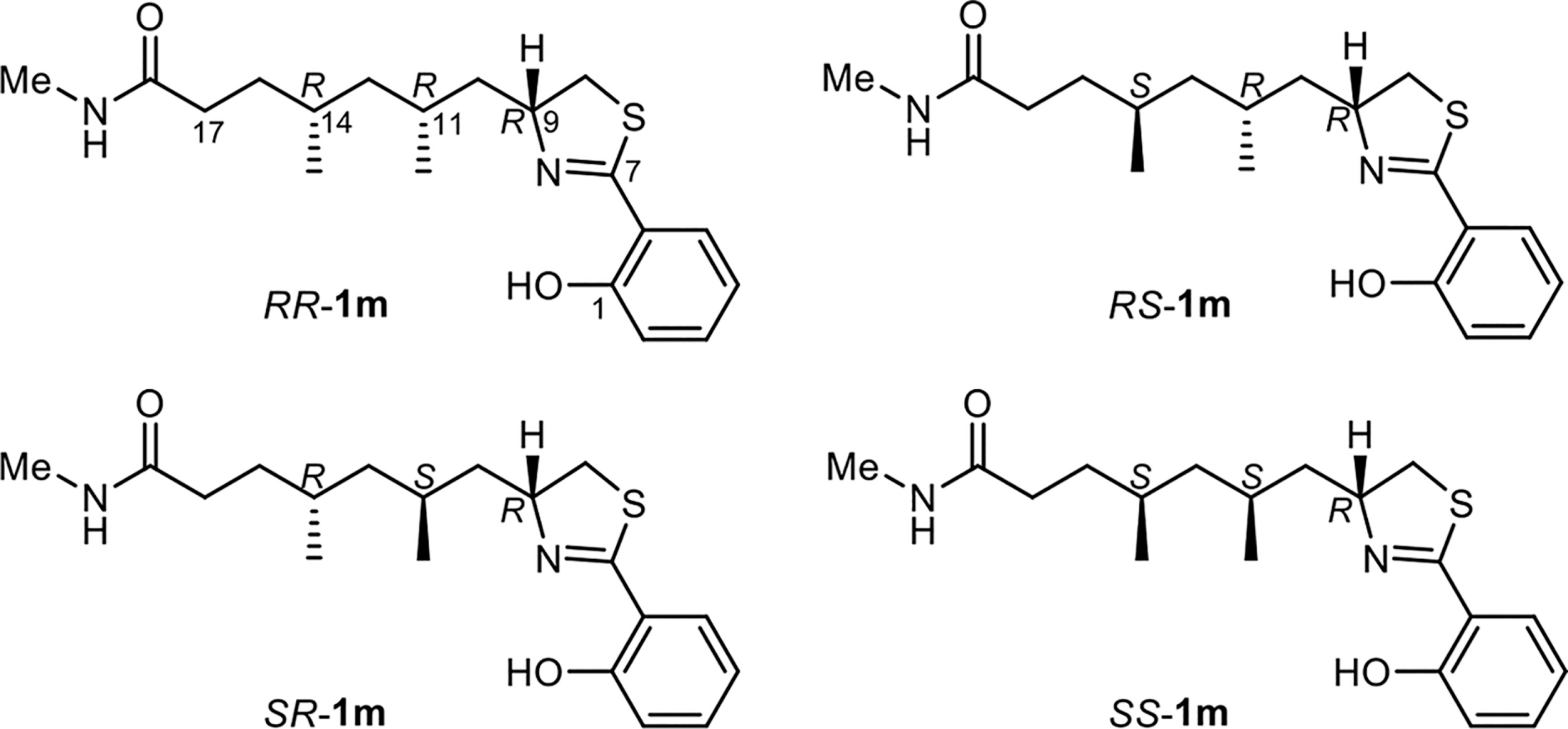
Four diastereomeric model compounds used for computational studies.

In the absence of unambiguous genetic or spectroscopic evidence, the configurations at C-11 and C-14 were studied computationally using quantum mechanical prediction of ^1^H and ^13^C NMR chemical shifts of the possible four stereoisomers.^44^ The simplified model compounds of fatuamide A, 11*R*,14*R*-**1m**, 11*R*,14*S*-**1m**, 11*S*,14*R*-**1m**, and 11*S*,14*S*-**1m** (hereafter *RR*-**1m**, *RS*-**1m**, *SR*-**1m**, and *SS*-**1m**, Figure 6) were chosen for calculations, because using the full fatuamide A molecule would have made computational time per conformer much longer and, what is worse, the number of conformers untreatably high.

Preliminary conformational studies on the phenylthiazoline moiety revealed that the conformation with the phenyl ring coplanar with the C=N bond and H-bond between OH at C-1 and the thiazoline N atom is largely predominant, while the thiazoline ring can exist in two twist conformations of comparable energy (_8_T^9^ and ^8^T_9_, Figure S17). Conformational grid searches were performed for the diastereomeric model compounds *RR*-**1m**, *RS*-**1m**, *SR*-**1m**, and *SS*-**1m** in the MMFF94 force field.^45^ For each stereoisomer, conformers within 4.5 kcal/mol from the lowest-energy conformer were optimized quantum mechanically at the B3LYP/6-31G(d) level of theory. Relative energies of optimized conformers were then evaluated using the B3LYP/6-311G+(d,p) level of theory and the PCM continuous solvent model, and relative populations of conformers were calculated from these using Boltzmann statistics. The conformational ensembles of *RR*-**1m**, *RS*-**1m**, *SR*-**1m**, and *SS*-**1m** used for NMR calculation were created selecting the lowest energy conformers accounting for at least 95% population (Table S2 for details of the conformational search).

Two different approaches were used to predict ^1^H and ^13^C NMR chemical shifts. The first approach, using the protocols and scaling factors recently proposed by Cohen et al.,^46^ was chosen because it is accurate and efficient, provides an indication of the general quality of the computational work, and indirectly of the conformational analysis work on which it is based. Isotropic shieldings were calculated using the GIAO method, separately for ^13^C and ^1^H, and converted into chemical shifts using the suggested precalculated scaling factors (“Method 1” in ref (46).). Comparison of chemical shifts (Table S3) included all protons and carbons between positions 1 and 17, except for C-7 and C-8, because prediction of ^13^C NMR chemical shifts of carbons linked to elements in rows three and beyond of the periodic table, such as S, is inaccurate unless relativistic effects are included. Root mean square deviations (RMSD) and mean absolute errors (MAE) were calculated and used to evaluate the match between experimental and calculated chemical shifts. Computational results clearly ruled out the 11*R*,14*R* and 11*S*,14*S* isomers (both with *syn* methyl groups), because they showed significantly higher RMSD and MAE than the 11*R*,14*S* and 11*S*,14*R* isomers (Table S3). The 11*R*,14*S* and 11*S*,14*R* stereoisomers showed both a very good agreement between predicted and experimental chemical shift, equal to or better than that expected^46^ for the protocol used; however, *RS*-**1m** showed a remarkably lower ^1^H RMSD (0.090 ppm vs 0.104 ppm for *SR*-**1m**) but *SR*-**1m** showed a slightly lower ^13^C RMSD (1.32 ppm vs 1.36 ppm for *RS*-**1m**), which prevented a confident configurational assignment.

Due to this ambiguity in the results, an additional approach was applied. Isotropic shielding values were recalculated at the mPW1PW91/6-311+G(d,p)/PCM(MeOH) level of theory for use with the DP4+ statistical method,^47^ which was specifically created to discriminate in an unbiased statistical way between isomers giving similar predicted chemical shifts. The DP4+ analysis (Figure S18) provided 87.8% probability for *RS*-**1m** to be the correct stereoisomer, 12.2% probability for *SR*-**1m**, and negligible probability for the remaining stereoisomers. Again, DP4+ results demonstrated the relative configuration of the methyl groups at C-11 and C-14 of fatuamide A (**1**) to be *anti*. However, even DP4+ only suggested but did not definitively prove that the 11*R*,14*S* configuration was correct, partly because the DP4+ method is known to overestimate the probability rate of the best-matching stereoisomer.^48^

A higher-level calculation was attempted to overcome this problem. Fatuamide A (**1**) is a flexible molecule, and even the truncated model compounds **1m** were each predicted to exist in over 50 significantly populated conformers. In this situation, an imperfect prediction of the conformational ensemble (geometry and relative energy of conformers) is a likely reason for errors in a quantum mechanical study.^49^ Therefore, the geometries of the conformers of *RS*-**1m** and *SR*-**1m** were reoptimized at the B3LYP/6-311G+(d,p) level of theory using the SMD solvation model, which has been shown to provide an improved estimation of the conformational distribution of flexible polar molecules compared to PCM,^48^ and energies of conformers were re-evaluated at the same level of theory for the new geometries. The isotropic shielding values of *RS*-**1m** and *SR*-**1m** were recalculated using the new conformational ensemble, and DP4+ analysis (Figure S19) showed a satisfactory 97.6% probability for *RS*-**1m** being the correct stereoisomer, finally proposing the 9*R*,11*R*,14*S*,19*S*,27*R*,29*S*,32*S*,40*R* absolute configuration for fatuamide A (**1**).

### Siderophore and Metal Binding Assay

The chrome azurol S (CAS) siderophore binding assay was used to evaluate the iron binding capabilities of the fatuamide A (**1**) producing culture.^50^ Changes in absorption and color indicate release of iron from the CAS reagent, and therefore the presence of a siderophore.^51^ The fatuamide producing culture, ASX22JUL14-2, dramatically decreased the absorbance at 655 nm of the CAS solution, indicating the presence of a siderophore (Figure S20, Table S4).

To further explore the potential metal binding capabilities of **1**, it was analyzed by native electrospray MS with post LC metal infusion (Figure S21), a method established for metabolomics studies of metal binding compounds.^52^ Prominent peaks were observed for those corresponding to the protonated form of **1** with an [M + H]^+^*m*/*z* of 819.4000 as well as the Cu^2+^ bound form [M – H^+^ + ^63^Cu^2+^] having an observed Δ *m*/*z* = 60.91 compared to the protonated form. Other metal adducts were observed in smaller quantities, indicating that fatuamide A can promiscuously bind other metals such as Zn^2+^ {[M – H^+^ + ^64^Zn^2+^], observed Δ *m*/*z* = 61.91}, and Fe^3+^ {[M – 2H^+^ + ^56^Fe^3+^], observed Δ *m*/*z* = 52.91}.

The relatively selective binding of copper suggested that fatuamide A might also be part of a copper detoxification system. Cyanobacteria are known to be sensitive to this metal and have evolved various strategies to survive in ecological niches that possess elevated copper levels.^53^ Average global concentrations of copper in coastal seawater are about 2 μg/L; however, in anthropogenically affected sites, copper concentrations can increase to as high as 25 μg/L.^54,55^ Therefore, we systematically evaluated the ability of this *Leptolyngbya* sp. strain to survive and grow under elevated levels of copper. Remarkably, levels exceeding 1000-fold natural seawater concentrations of copper were tolerated and growth of this strain at these high copper concentrations was nearly indistinguishable from native seawater controls (Table S5 and Figure S22). It was only at copper levels of 2500-fold over natural seawater concentrations that detrimental effects on the health of this *Leptolyngbya* sp. strain were observed, demonstrating that this strain is highly resistant to the toxic effects of copper, and we speculate that the production of **1** likely assists in this capacity. This contrasts with our previous observation that another strain of *Leptolyngbya* sp. is quite sensitive to copper, showing significant stress at 0.75-fold average coastal seawater concentrations after just 2 days.^18^

### Cytotoxicity and Anti-Inflammatory Assays

All nine fractions of the ASX22JUL14-2 and the parent crude extract were tested in two biological assays; cytotoxicity toward NCI-H460 human non-small lung carcinoma cells and anti-inflammatory effects using the RAW264.7 murine macrophage cell line. Screening results are shown in Figures S1 and S2. None of the samples demonstrated anti-inflammatory activity while fraction I possessed cytotoxic activity toward NCI-H460 cells. Despite being isolated from the cytotoxic fraction I, fatuamide A (**1**) as the apo form did not show any NCI-H460 cytotoxicity in the range of 2 nM to 60 μM using ten half-logarithmic concentrations.

## Conclusions

The novel natural product fatuamide A (**1**) was isolated from cultures made from a field collected *Leptolyngbya sp.*, a marine cyanobacterium obtained from American Samoa. This is only the third phenolate type siderophore to be reported from a cyanobacterium which includes the cyanochelins^56^ and leptochelin,^17^ and one of a very few known from a marine species. The structure was determined through an integrated use of NMR data, mass spectrometry, bioinformatic analysis of the biosynthetic gene cluster, and in-depth computational analyses. Fatuamide A (**1**) was initially prioritized for isolation because fractions containing this compound possessed strong cytotoxicity against NCI-H460 lung cancer cells. However, at a maximum tested concentration of 49.5 μg/mL (60 μM), it was found to be inactive, and therefore, we speculate that another minor compound in the fraction, or a synergistic pair of metabolites, was responsible for the observed cytotoxicity. Through analysis of the biosynthetic gene cluster, LC-MS/MS studies and specific metal binding assays, it was shown that **1** is a metallophore of highly unique structure and with a distinctive selectivity for the binding of copper. Metallophores have potential biomedical and agricultural utility as well as environmental relevance by virtue of their capacity to sequester metals. In this case, the remarkable resistance of this *Leptolyngbya* sp. strain to the growth inhibitory effects of elevated copper levels may allow it to occupy and thrive in habitats that are toxic to other organisms.

Curiously, it appears that two natural products are encoded in a single biosynthetic gene cluster locus in this organism, one of which, fatuamide A (**1**), is the shorter version of the other, proposed metabolite “fatuamide B” (Figures S10, S11 and Table S1). Because there is a proposed terminating condensation unit in the biosynthetic gene cluster after installation of the final glycine in fatuamide A, we propose this is one of two potential terminations of the fatuamide gene cluster. Correspondingly, there is a second proposed terminating condensation unit after the installation of the predicted final proline residue in fatuamide B, thus providing a second and alternative termination of the pathway. These two proposed terminating condensation units allow for this cyanobacterium to produce two natural products from a single biosynthetic gene cluster, similar to what was observed in the production of the vatiamides,^57^ again demonstrating the remarkable ingenuity of nature to optimize the efficient production of structurally diverse natural products.

## Experimental Section

### General Experimental Procedures

UV data were obtained during LCMS analyses using a Finnigan Surveyor PDA Plus Detector. ECD data were obtained on an Aviv model 215 CD spectrometer in MeOH at a concentration of 1 mg/mL using a path length of 2 mm. Three scans were recorded with data collected every 0.5 nm, and the graph was smoothed using Spectragryph software.^58^ IR data was collected on a Thermo Scientific Nicolet 6700 FT-IR instrument. ROESY, TOCSY, HSQC-TOCSY, H2BC, and band selective HMBC NMR spectra were recorded using a 1.7 mm triple resonance TCI cryoprobe on a Bruker AVANCE III 600 MHz with standard Bruker pulse sequences at 298 K. ^1^H NMR, HSQC, HMBC, and COSY spectra were recorded on a JEOL 500 MHz NMR spectrometer at 298 K. ^13^C NMR data were recorded at 298 K with standard pulse sequences on a Varian VX 500 NMR with a cold probe and z-gradients. NMR data were recorded in MeOH-*d*_4_ and calibrated using the solvent peaks (δ_H_ 3.31, δ_C_ 49.00). LC-MS/MS analysis was performed using a Thermo Finnigan Surveyor HPLC System with a Thermo-Finnigan LCQ Advantage Max Mass Spectrometer equipped with a Phenomenex Kinetex 5 μm C18 100 × 4.6 mm column. A linear gradient was used with a flow rate of 0.6 mL/min and solvents (A) H_2_O + 0.1% formic acid (FA) and (B) CH_3_CN + 0.1% formic acid. A 5 min isocratic step of 30% B in A was followed by an increase to 99% B over 17 min. It was held at 99% B for 5 min and then decreased to 30% B in 1 min and then held for 4 min at 30% B. Mass spectra were obtained with an ESI source (*m*/*z* 200–2000). HR ESI MS data were collected at the UCSD Chemistry and Biochemistry Mass Spectrometry Facility on an Agilent 6230 Accurate-Mass TOFMS in positive ion mode. A CombiFlash EZ Prep Lumen flash chromatography TELEDYNE ISCO system was used for chromatography. The metal infusion method was performed using literature methods with a Vanquish UPLC system coupled to a Thermo Fisher Scientific Q-Exactive orbitrap mass spectrometer.^52^ Photomicrographs were taken with a Life Technologies EVOS XL Digital Inverted Microscope equipped with an Olympus 100X Plan S-APO Oil (AMEP-4733) objective.

### Biological Material Collection and Identification

The sample ASX22JUL14-2 was collected in Faga’itua Bay, American Samoa on 22 July 2014. A bulk sample collection for chemical analysis, an RNA*later* sample, and a living culture sample was collected from 1 to 2 m water depth using snorkel gear. The living sample was cultured for chemical analysis for approximately 120 days in a 16-h light/8-h dark protocol in SWBG11 media at 27.2–27.3 °C.

### Culture Techniques

The culture of ASX22JUL14-2 was scaled up in 13 and 9 L glass carboys, each outfitted with a rubber stopper. Aeration was provided using an air pump with a HEPA filter connected to an autoclavable tube and run through the stopper and to the bottom of the carboy. A shorter second tube between the head space of the carboy and the exterior, also connected to a HEPA filter, balanced the air flow. After autoclaving the entire carboy and tube system, 10 L of sterile media were added and ASX22JUL14-2 filaments from a 2 L starter culture were added in a biosafety cabinet. The cultures were grown in a 16-h light/8-h dark protocol at 27.2–27.3 °C and harvested after 3–5 months of growth.

### Extraction and Isolation

The cultured biomass was collected by filtration and extracted using CH_2_Cl_2_:MeOH (2:1) for 30 min with sonication at <30 °C. The CH_2_Cl_2_ layers from partitioning with H_2_O were combined and dried *in vacuo* to yield an extract of 1.16 g. The extract was further fractionated by vacuum liquid chromatography (VLC), progressively using mixtures of hexanes, EtOAc and MeOH. The most polar eluted fraction (fraction I, eluted with 100% MeOH) afforded 1.011 g of material, contained fatuamide A (**1**), salts, and silica gel. This fraction was loaded onto a Combiflash EZ Prep system (Teledyne Isco) in the solid phase using Celite Filter Aid and a C_18_ 5.5 g column (RediSep Gold C18 Reversed-Phase) that was eluted with (A) H_2_O and (B) MeOH and (C) CH_3_CN at a flow rate of 18 mL/min and monitored at wavelengths of 214 and 254 nm. The elution was initiated with isocratic conditions of 30% (B) and 70% (A) for 6 min followed by a gradient to 100% (B) until minute 21, held for 1 additional min, and then at minute 22 eluted with 50% (B) and 50% (C) for 3 min, held for one additional min and then decreased to 20% (B) and 80% (C) and held for one min, followed by 100% (C) until minute 33.8. Under these conditions, fatuamide A eluted at 16 min, and a total of 1 mg was obtained.

#### Fatuamide A

White amorphous solid; UV λ_max_ 219, 236, 271 nm; ECD (1.22 mM, MeOH) λ_max_ 213 nm (+0.53), 220 nm (+0.53); IR (neat) *v*_max_ 3414, 2922, 2853, 1632, 1602, 1497, 1457, 1384, 1308, 1255, 1220, 1155, 1081, 950, 800, 753, 701 cm^–1^; ^1^H NMR and ^13^C NMR data (500 MHz, MeOH-*d*_4_), Table 1; HR-ESI-TOFMS *m*/*z* 819.4000 [M + H]^+^ (calcd C_44_H_59_N_4_O_9_S, 819.4003); publicly available NMR data are at https://np-mrd.org/natural_products/NP0333788.

### Cytotoxicity and Anti-Inflammatory Assays

NCI-H460 human lung carcinoma cells were grown in a flask in monolayers to near confluence and seeded at 6.66 × 10^3^ cells/mL in 96-well microtiter plates (180 μL each) containing RPMI medium with FBS (Fetal Bovine Serum). They were incubated for 24 h at 37 °C and 5% CO_2_. The test samples were prepared by dissolving in DMSO and diluted in RPMI medium such that the final DMSO concentration was less than 1%. A 20 μL aliquot of these solutions was added to each well and the final concentrations of the samples were 10 and 1 μg/mL; these were tested in duplicate. Plates were incubated for 48 h and then stained for 25 min with MTT [3-(4,5-dimethylthiazol-2-yl)-2.5-diphenyltetrazolium bromide]. The plates were analyzed by optical density measurement at 570 and 630 nm. Cell survival rates were *calculated* by comparison with negative controls that were comprised of RPMI medium by itself.

Anti- and pro-inflammatory activities were determined using RAW 264.7 ATCC murine macrophage cells (TIB-71) in Dulbecco’s Modified Eagle Medium (DMEM) with 10% endotoxin-low fetal bovine serum at 37 °C with 5% CO_2_. The cells were seeded in 96-well microtiter plates (5 × 10^4^ cells/well) in triplicate and subjected for 24 h to treatment with 3 μg/mL of *E. coli* lipopolysaccharide (LPS) and the tested fractions at concentrations of 10 and 30 μg/mL. The accumulation of nitric oxide (NO) in the supernatant of the cell cultures was analyzed by the quantification of nitrite produced by the Griess reaction. A 50 μL aliquot of each supernatant was added to 96-well microtiter plates with 50 μL of 0.1% sulfanilamide and 50 μL 0.1% *N*-(1-naphthyl)-ethylenediamine (NED). Absorbance of the mixtures was quantitatively measured at 570 nm to calculate the release of NO based on a standard curve from a nitrate standard in DMEM (0–100 μM). The negative control (0% anti-inflammation) was formed by the addition of only LPS to the cell culture whereas the addition of 1% DMSO to the complete experimental condition was used as the positive anti-inflammation control.

### Bioinformatics

Cyanobacterial DNA was extracted as reported and sequence information can be found on NCBI with the accession JAAHFU000000000.^4^ Alignments of the KR domains were performed with *Geneious version 2019.2* created by Biomatters with a Global Alignment (Needleman-Wunsch protocol).

### Computational Methods

Pcmodel (v. 10.075000) was used for conformational searches and molecular mechanics optimizations, Gaussian 16 (Revision C.01) was used for all the for quantum mechanical calculations, OpenBabel (v. 3.0.0), GaussView (v. 6.0.16), and VMD (v. 1.9.3) for format conversion, data analysis, and visualization.

Random, fully staggered conformers of the four diastereomeric model compounds *RR*-**1m**, *RS*-**1m**, *SR*-**1m**, and *SS*-**1m** were used as starting structures for a systematic conformational search (Grid Search in Pcmodel) involving the seven dihedral angles between C-9 and C-18 in steps of 120° and the two twist conformations of the thiazoline ring. Each conformer was then minimized in the MMFF94 force field^45^ with the Generalized Born/Surface Area (GBSA) implicit solvation model as implemented in PCModel 10, and duplicate conformers (RMSD < 0.1 Å) were removed.

Conformers within 4.5 kcal/mol (MMFF94 energy) from the lowest energy conformer of each stereoisomer were selected for quantum mechanical optimization, which was performed using Gaussian 16 at the B3LYP/6-31G(d) level of theory in vacuo. After removal of duplicated conformers (RMSD < 0.1 Å), relative energies of optimized conformers were evaluated using the B3LYP/6-311G+(d,p) level of theory and the PCM solvation model, and were used to calculate the population of each optimized conformer with the Boltzmann distribution law at 298 K. Finally, the conformers of each stereoisomer were ranked in order of increasing energy/decreasing population and selected for the subsequent steps of calculations until 95% aggregate population was reached. The results of the conformational searches are summarized in Table S2, and the Cartesian coordinates of all the conformers used for NMR calculations, as well as their energies, are reported as Supporting Information in the text file np4c01051_si_002.txt.

An enhanced conformational ensemble was calculated for model compounds *RS*-**1m** and *SR*-**1m**, by reoptimizing the conformers above at the B3LYP/6-311G+(d,p) level of theory and the SMD solvation model, and using the energies of the reoptimized geometries. The Cartesian coordinates and energies of these conformers are reported as Supporting Information in the text file np4c01051_si_003.txt, and the lowest energy conformer of model compound *RS*-**1m** is depicted in Figure S23.

Chemical shift prediction was performed according to Cohen et al.;^46^ isotropic shieldings of individual conformers were calculated using the GIAO method, separately for ^13^C [ωB97X-D/def2-SVP/PCM(MeOH) level of theory] and ^1^H [WP04/jul-cc-pVDZ/PCM(MeOH) level of theory]. Average isotropic shieldings were calculated according to the population of each conformer, and were converted into chemical shifts using the suggested precalculated scaling factors, δ = (195.6683 – σ)/1.0081 for ^13^C and δ = (31.8883 – σ)/1.0309 for ^1^H (Table S3).

Isotropic shieldings for DP4+ analysis were calculated at the highest level of theory parametrized,^47^ namely mPW1PW91/6-311+G(d,p) with PCM solvation model, and average isotropic shieldings were directly used in the Excel spreadsheet provided (Figures S18 and S19).^47^

While evaluating chemical shift deviations or applying the DP4+ method, ^1^H NMR chemical shift of methyl protons was calculated as the average of chemical shifts of the three protons; the assignment of the proR and proS protons of diastereotopic pairs was chosen to give better agreement with the predicted chemical shifts.

### Chrome Azurol S Assay

A chrome azurol S (CAS) assay was modified for use with seawater samples.^50,51,59^ The CAS assay solution consisted of CAS (chrome azurol S, 2 × 10^–4^ M), FeCl_3_ (2 × 10^–5^ M), HDTMA (hexadecyltrimethylammonium bromide, 1.6 × 10^–3^ M), and perazindiethanesulfonic acid (PIPES) at pH 5.8 (1.1 × 10^–1^ M). After 50 h of incubation with cultures of *Leptolyngbya* sp. at 27.2 °C, the absorbance was measured at 655 nm. The concentrations of Fe(III) complexes were calculated from the absorbance. A first negative control was comprised of the solution containing the ASX22JUL14-2 culture in its media but without added CAS reagent. A second negative control was the CAS assay solution by itself (e.g., no added cyanobacterial culture). A third negative control was comprised of CAS assay solution in pure H_2_0 (1:1). The final assay condition was comprised of ASX22JUL14-2 in full strength metal mix media and directly assayed with the CAS reagent, a condition which gave a positive reaction (Figure S20).

### Copper Toxicity Experiments

Standard SWBG11 medium^18^ was prepared, however, without one of the metal mix components that contains copper (BG#8) (termed “SWBG11-8”). A concentrated stock solution of CuSO_4_·5H_2_O was prepared at 100 mg/5 mL of SWBG11-8 medium (termed Cu-stock). This stock solution was sonicated for 10 min to ensure that the CuSO_4_·5H_2_O was fully dissolved. Next, 500 mL of the metal mix stock solution BG#8 was prepared with all required components except CuSO_4_·5H_2_O (termed BG#8-Cu). This BG#8-Cu solution was sonicated for 40 min at room temperature to ensure complete dissolution. A defined volume of BG#8-Cu was aliquoted into prelabeled Falcon tubes and the appropriate volume of the Cu-stock solution was added to create solutions of modified BG#8 at the desired concentrations of copper. These solutions with varying concentrations of copper were filter sterilized before adding to SWBG11-8 media, for a total volume of 35 mL within glass culture tubes (25 × 150 mm). Therefore, the components of the SWBG11 media were unchanged from the original recipe except for the concentrations of copper (Table S3). For the highest copper concentrations where the amount of Cu-stock solution would exceed the amount of BG#8 needed for the standard media preparation (e.g., in media with greater than 1000-fold the average coastal seawater concentrations of 2 μg/L), the copper solution was prepared directly in SWBG11-8 and added directly into the media along with the appropriate amount of BG#8-Cu. This ensured that the concentrations of the other nutritional components were unaltered when compared to standard SWBG11 medium.

After inoculation, the cultures were maintained at 26 °C with a 16 h light/8 h dark regimen. The cultures were assessed for health and viability using a predesigned five-point grading system (Table S5). On Day 14, cultures were harvested and microscopy images were obtained using an EVOS XL Core Microscope (Life Technologies). Data were processed using Microsoft Excel v. 16.72 (2023).

### Native Metabolomics

Native metabolomics experiments were performed using a Vanquish Horizon UHPLC system coupled to a Q-Exactive HF quadrupole Orbitrap mass spectrometer (Thermo Fisher Scientific) with an Vanquish quaternary pump as a makeup pump, as previously described in detail.^60^ For reversed-phase separation, a C18 core–shell microflow column (Kinetex C18, 100 × 1 mm, particle size of 1.8 μm, pore size of 100 Å, Phenomenex) was used. The mobile phase consisted of solvent A (H_2_O + 0.1% FA) and solvent B (MeCN + 0.1% FA). The flow rate was set to 100 μL min^–1^. A linear gradient of 5–50% B between 0 and 8 min and 50–99% B between 8 and 10 min was followed by a 2 min washout phase at 99% B and a 6 min re-equilibration phase at 5% B. DDA of MS/MS spectra was performed in the positive ion mode. The makeup flow of ammonium acetate buffer (10 mM) was set to 100 μL min^–1^ and infused postcolumn through a T-splitter.

Iron, zinc and copper solutions were infused postcolumn and postmake-up through a second T-splitter at a flow rate of 5 μL min^–1^. ESI sheath gas was set to 40 arbitrary units (a.u.), the auxiliary gas flow was set to 10 au and the sweep gas flow to 0 au. The auxiliary gas temperature was set to 200 °C. The spray voltage was set to 3.5 kV and the inlet capillary was heated to 320 °C. The S-lens level was set to 70 V applied. The MS scan range was set to 200–2,000 *m*/*z* with a resolution at *m*/*z* 200 (R *m*/*z* 200) of 70,000. The maximum ion injection time was set to 100 ms with an AGC target of 5 × 10^5^. Up to two MS/MS spectra per duty cycle were acquired at R *m*/*z* 200 17,000 with one microscan. The maximum ion injection time for MS/MS scans was set to 100 ms with an AGC target of 5.0 × 10^5^ ions and a minimum 5% AGC. The MS/MS precursor isolation window was set to *m*/*z* 1.

## Supporting Information Available

Figures S1–S36 and Tables S1-S5 referenced in the text, NMR, IR and other spectroscopic data of fatuamide A, tables used in DFT calculations, and observational data on the effects of copper on cyanobacterial health (PDF)
Cartesian coordinates and energies of all the conformers of model compounds *RR*-**1m**, *RS*-**1m**, *SR*-**1m**, and *SS*-**1m** (TXT)
Cartesian coordinates and energies of all the reoptimized conformers of model compounds *RS*-**1m** and *SR*-**1m** (TXT)

## Dedication

Dedicated to Dr. Sheo B. Singh, retired from Merck, now with Drew University and Stevens Institute of Technology, for his pioneering work on bioactive natural products.
