# Supplementary material for: Fatuamide A, a Hybrid PKS/NRPS Metallophore from a Leptolyngbya sp. Marine Cyanobacterium Collected in American Samoa
Source: J Nat Prod. 2025 Jan 29;88(2):322–35. doi: 10.1021/acs.jnatprod.4c01051 (PMC11877528; doi:10.1021/acs.jnatprod.4c01051)
Supplement: Supplementary file 1 — np4c01051_si_001.pdf [file np4c01051_si_001.pdf]

Supporting Information

Fatuamide A, a Hybrid PKS/NRPS Metallophore from a *Leptolyngbya* sp. Marine Cyanobacterium Collected in American Samoa

*Kelsey L. Alexander[1,2], C. Benjamin Naman[1,3], Arihiro Iwasaki[1,4], Alfonso Mangoni[5], Tiago Leao[6], Raphael Reher[7,8], Daniel Petras[9,10], Hyunwoo Kim[1,11], Eva Ternon[1,12], Eduardo J. E. Caro-Diaz[1,32], Evgenia Glukhov[1], Jana A. Mitrevska[1], Nicole E. Avalon[1], Brendan M. Duggan[7], Lena Gerwick[1] and William H. Gerwick[1,7]**

[1]Center for Marine Biotechnology and Biomedicine, Scripps Institution of Oceanography, University of California, San Diego, La Jolla, California 92093, United States

[2]Department of Chemistry, University of California, San Diego, La Jolla, California 92093, United States

[3]Department of Science and Conservation, San Diego Botanic Garden, Encinitas, California 92024, United States

[4] Department of Chemistry, Faculty of Science and Technology, Keio University, 3-14-1 Hiyoshi, Kohoku-ku, Yokohama, Kanagawa 223-8522, Japan

[5] Dipartimento di Farmacia, Università degli Studi di Napoli Federico II, via Domenico Montesano 49, Napoli 80131, Italy

[6]Institute of Chemistry, São Paulo State University (UNESP), Araraquara, Brazil.

[7]Skaggs School of Pharmacy and Pharmaceutical Sciences, University of California, San Diego, La Jolla, California 92093, United States

[8]Institute for Pharmaceutical Biology and Biotechnology, Department of Pharmacy, Philipps-University Marburg, Robert-Koch-Straße 4, 35037 Marburg, Germany

[9]Interfaculty Institute of Microbiology and Infection Medicine, University of Tuebingen, Tuebingen, Germany

[10]Department of Biochemistry, University of California, Riverside, CA, USA

[11]College of Pharmacy, Dongguk University, Goyang, South Korea

[12] Sorbonne Université, CNRS, Laboratoire d'Océanographie de Villefranche (UMR 7093), 06230 Villefranche-sur-Mer, France

[13] Department of Pharmaceutical Sciences, School of Pharmacy University of Puerto Rico - Medical Sciences Campus, San Juan, Puerto Rico, 00935

Table of Contents

Figures

Figure S1. Chromatography fractions from the *Leptolyngbya* sp. extract tested against NCI H460 human lung carcinoma cells. 6

Figure S2. Fractions of *Leptolyngbya* sp. extract tested for anti-inflammation activity at A. 10 μg/mL and B. 30 μg/mL. 7

Figure S3. MS/MS-Based Molecular Network of ASX22JUL14-2 (using GNPS[1]). 8

Figure S4. HR-ESI-TOFMS of fatuamide A $[M+H]^+$ 819.4000. 9

Figure S5. Analysis of the MS/MS spectrum of fatuamide A by SIRIUS 4.0 software[2]. 10

Figure S6. FT-IR spectrum of fatuamide A. 11

Figure S7. A. Fatuamide A top 10 SMART 2.1 results based on cosine score. The colored boxes highlight the substructures that have similar motifs to those found in fatuamide A. **B.** Depiction of the HSQC spectrum of fatuamide A (**1**) with analysis from DeepSAT as to most likely compound class. 12

Figure S8. Comparison ^{13}C NMR shifts for yersiniabactin Isomer I and II and fatuamide thiazoline ring carbon atoms. 13

Figure S9. MS/MS of *m/z* 553 fragment of fatuamide A. 14

Figure S10. MS/MS analysis and structure of fatuamide B. 15

Figure S11. HR-ESI-TOFMS of fatuamide B $[M+H]^+$ 1321.5512. 16

Figure S12. Phylogenetic tree of cyanobacterial condensation units. 17

Figure S13. Alignment of different KR domains from cyanobacterial natural products made with Geneious version 2019.2 created by Biomatters. 18

Figure S14. Dendrogram of cyanobacterial C-methyl transferases (cMT). 19

Figure S15. Alignment of cyanobacterial ER domains using Geneious. 20

Figure S16. Analysis of ^{1}H NMR chemical shifts of different stereoisomers of 1,3-dimethyl substituted compounds compared to those in fatuamide A. 21

Figure S17. Conformations of the phenylthiazoline in fatuamide A (**1**). 22

Figure S18. The results of DP4+ statistical analysis of ^{1}H and ^{13}C NMR chemical shifts predicted for *RR*-**1m**, *RS*-**1m**, *SR*-**1m**, and *SS*-**1m**. 23

Figure S19. The results of DP4+ statistical analysis of ^{1}H and ^{13}C NMR chemical shifts predicted for *RS*-**1m** and *SR*-**1m**. 24

Figure S20. Chrome Azurol S (CAS) assay with fatuamide A producer ASX22JUL14-2. 25

Figure S21. LC-MS/MS trace of native electrospray mass spectrometry with post column metal infusion. 26

Figure S22. Heatmap with the visual grading of the health of cyanobacterial cultures. 27

Figure S23. The lowest energy conformer of model compound *RS*-**1m** at the B3LYP/6-311G+(d,p)/SMD level of theory. 28

Figure S24. HR-ESI-TOFMS MS/MS of fatuamide A. 29

Figure S25. ^{1}H NMR spectrum of fatuamide A in MeOH- d_4 on 500 MHz JEOL. 30

Figure S26. ^{13}C NMR spectrum of fatuamide A in MeOH-d_4 on Varian VX 500 MHz NMR. 31

Figure S27. ^{1}H-^{1}H COSY NMR spectrum of fatuamide A in MeOH-d_4 (500 MHz). 32

Figure S28. ^{1}H- ^{13}C HMBC spectrum of fatuamide A in MeOH-d_4 (500 MHz). 33

Figure S29. ^{1}H-^{13}C HSQC NMR spectrum of fatuamide A in MeOH-d_4 (500 MHz). 34

Figure S30. ^{1}H-^{13}C HSQC TOCSY spectrum of fatuamide A in MeOH-d_4 (600 MHz). 35

Figure S31. ^{1}H-^{1}H TOCSY spectrum of fatuamide A in MeOH-d_4 (600 MHz). 36

Figure S32. ^{1}H-^{13}C HMBC selective for δ_{13C} 115-143 ppm of fatuamide A in MeOH-d_4 (600 MHz). 37

Figure S33. ^{1}H-^{13}C HMBC selective for δ_{13C} 25-50 ppm of fatuamide A in MeOH-d_4 (600 MHz). 38

Figure S34. ^{1}H-^{13}C H2BC spectrum of fatuamide A in MeOH-d_4 (600 MHz). 39

Figure S35. ^{1}H-^{1}H ROESY spectrum of fatuamide A in MeOH-d_4 (600 MHz). 40

Figure S36. ECD spectrum of fatuamide A (**1**) in MeOH (1 mg/mL; 1.22 mM). 41

Tables

Table S1. A. Putative gene cluster for fatuamide B biosynthesis B**.** Proposed biosynthesis of fatuamide B. 42

Table S2. Results of conformational search for *RR*-**1m**, *RS*-**1m**, *SR*-**1m**, and *SS*-**1m**. 47

Table S3. Experimental chemical shifts, predicted chemical shifts, chemical shift errors, root mean square deviations (RMSD) of predicted chemical shifts, and mean absolute errors (MAE) of predicted chemical shift for *RR*-**1m**, *RS*-**1m**, *SR*-**1m**, and *SS*-**1m**. All values are expressed in ppm. 48

Table S4. Absorbance of the samples from the CAS assay at 655 nm. 49

Table S5. A. Five-point grading for assessing the visually observed health and viability of ASX22JUL4-2 cultures over 14 days in culture media with varying concentrations of dissolved copper performed at least in triplicate biological replicates. B. Example photos of cultures representing Grade 5, Grade 4, Grade 3, and Grade 2 of the scale used to visually assess health and viability of ASX22JUL14-2 50

References. 53

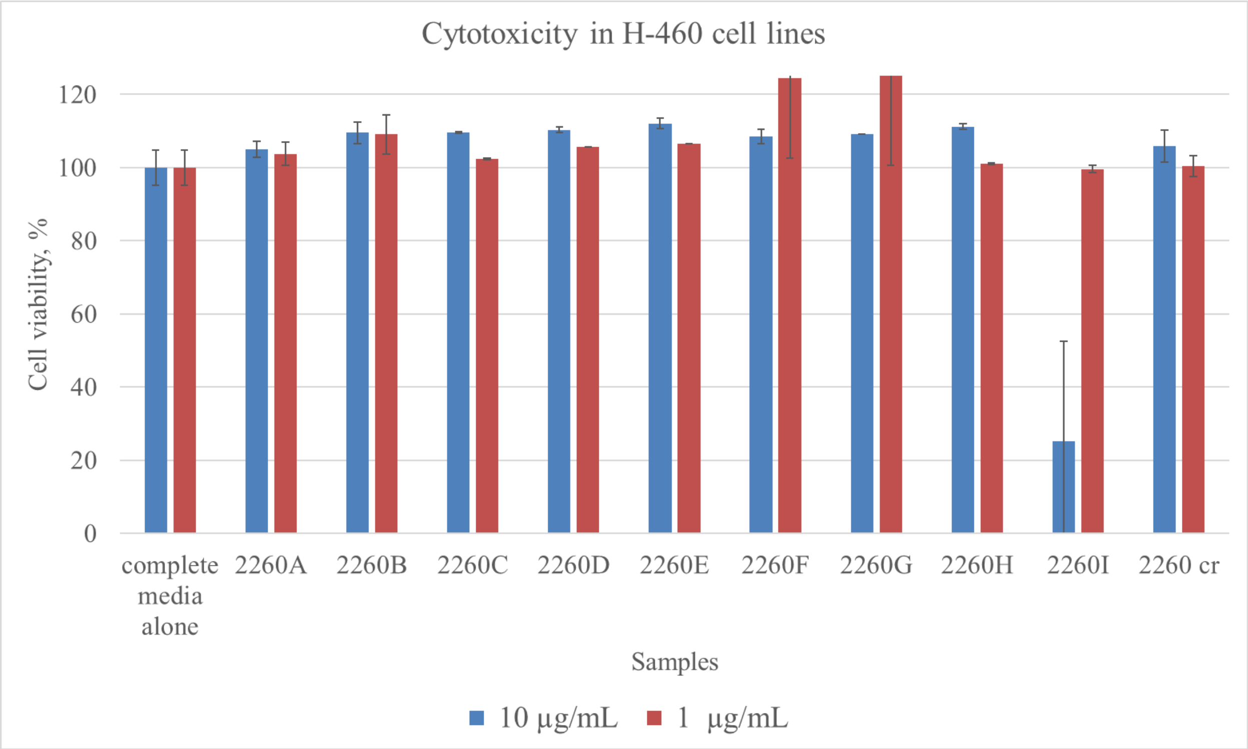

Figure S1. Chromatography fractions from the *Leptolyngbya* sp. extract tested against NCI H-460 human lung carcinoma cells.

A.

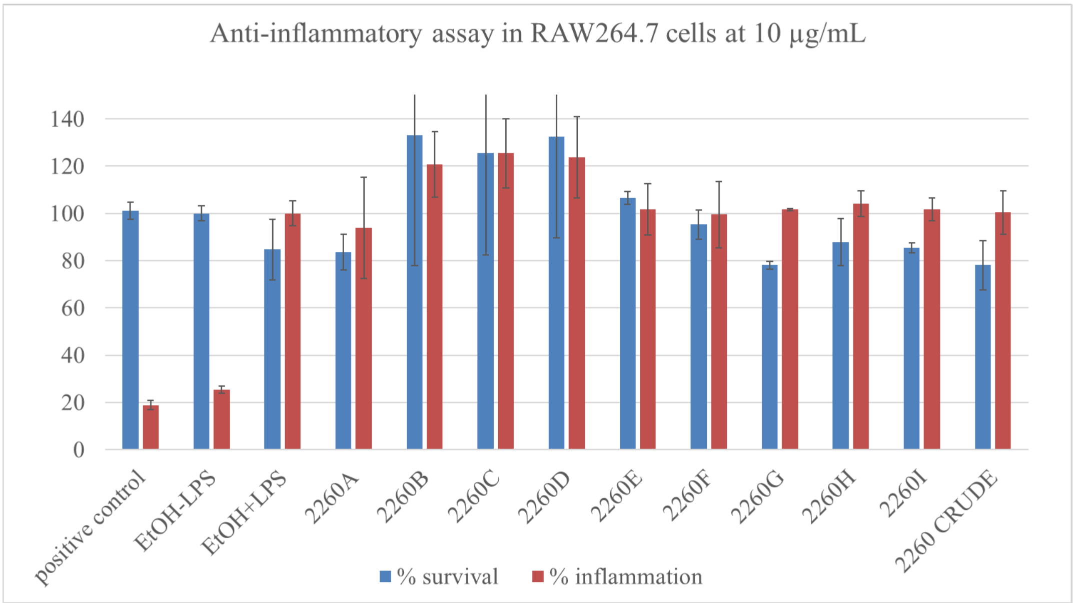

B.

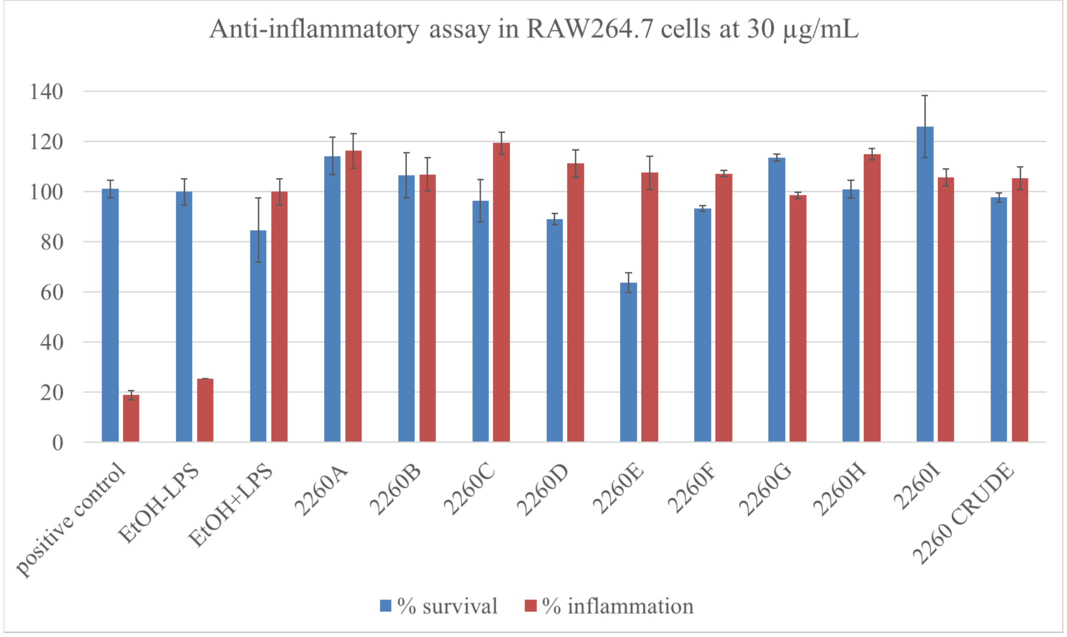

Figure S2.
Fractions of *Leptolyngbya* sp. extract tested for anti-inflammation activity at A. 10 µg/mL and B. 30 µg/mL. Note, all *Leptolyngbya* sp. 2260-derived samples were treated with LPS, as was the control 'EtOH+LPS'. Only the positive control and 'EtOH-LPS' were not treated with LPS.

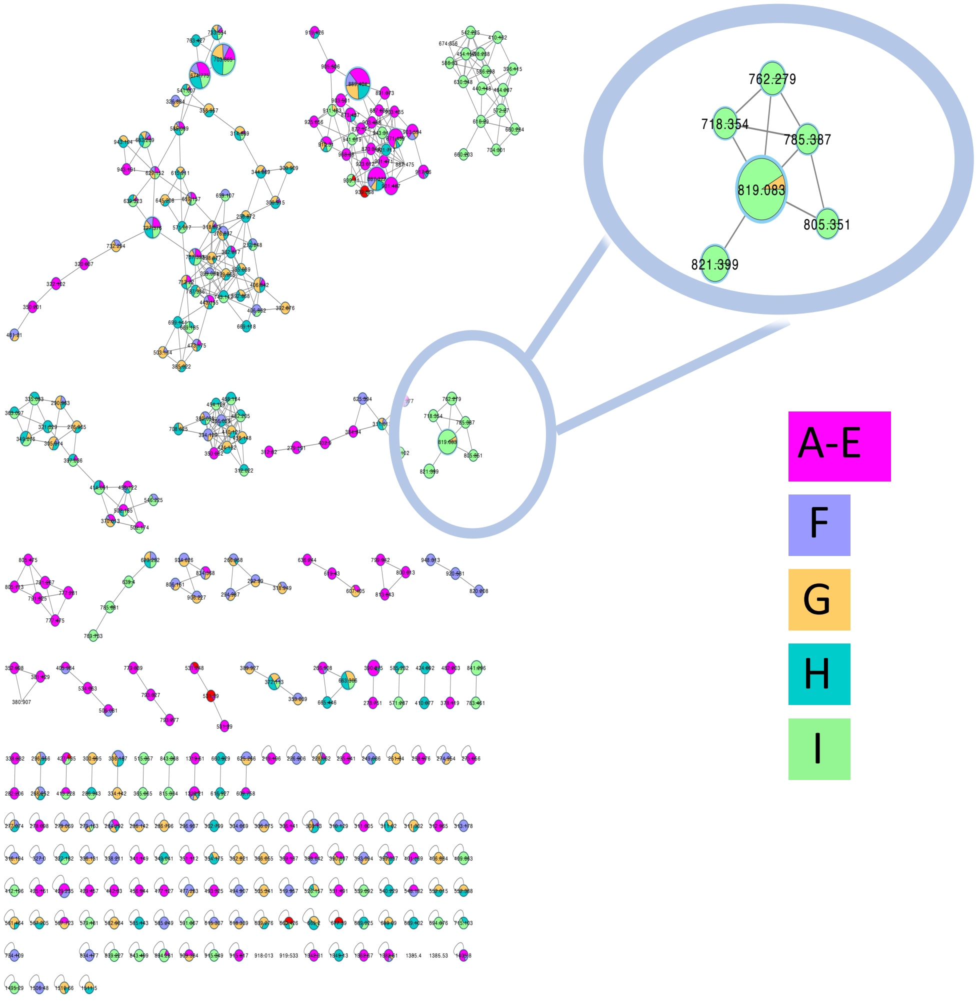

Figure S3. MS/MS-Based Molecular Network of ASX22JUL14-2 using GNPS[1]. Blue circle shows cluster from the most cytotoxic fraction I. Colored boxes with letters refer to the chromatographic fractions of the extract with 'A' being least polar and 'I' being the most polar.

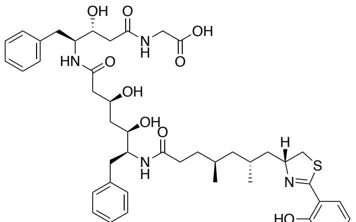

Fatuamide A (**1**)

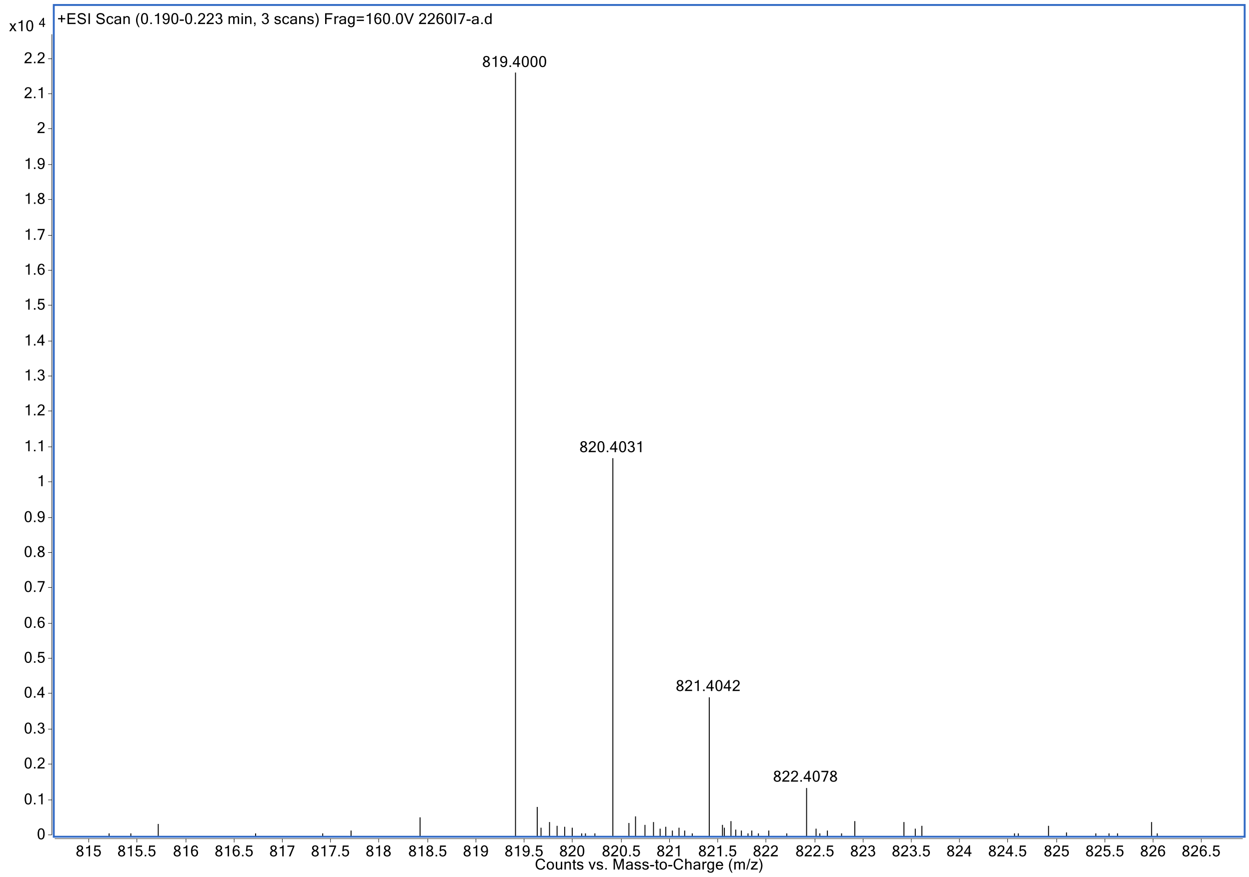

Figure S4. HR-ESI-TOFMS of fatuamide A $[M+H]^+$ 819.4000.

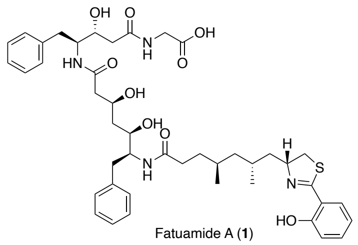

Fatuamide A (**1**)

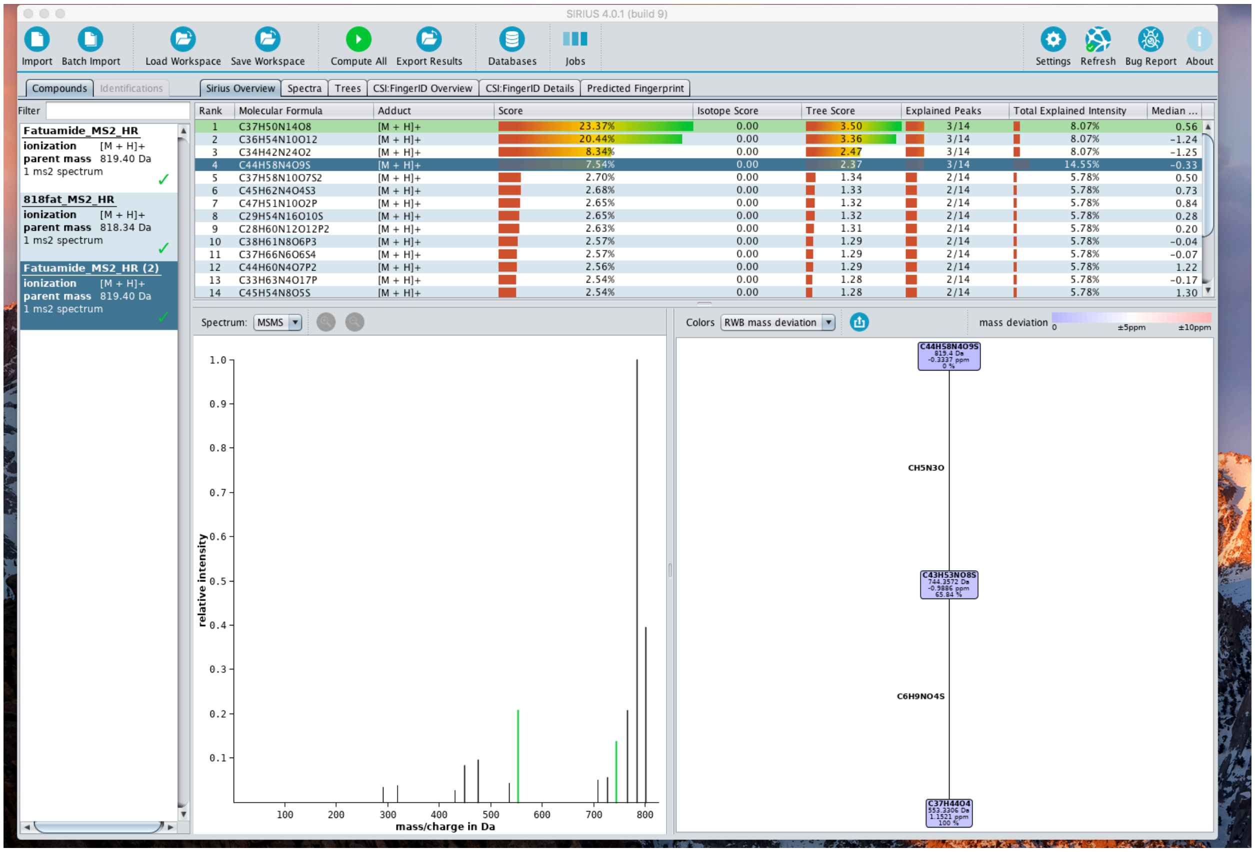

Figure S5. Analysis of the MS/MS spectrum of fatuamide A by SIRIUS 4.0 software[2]. The blue highlighted formula (rank 4) was determined to be the correct molecular formula.

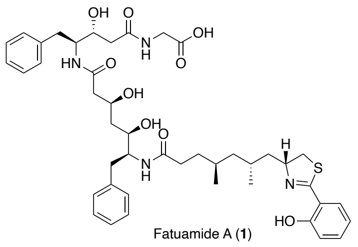

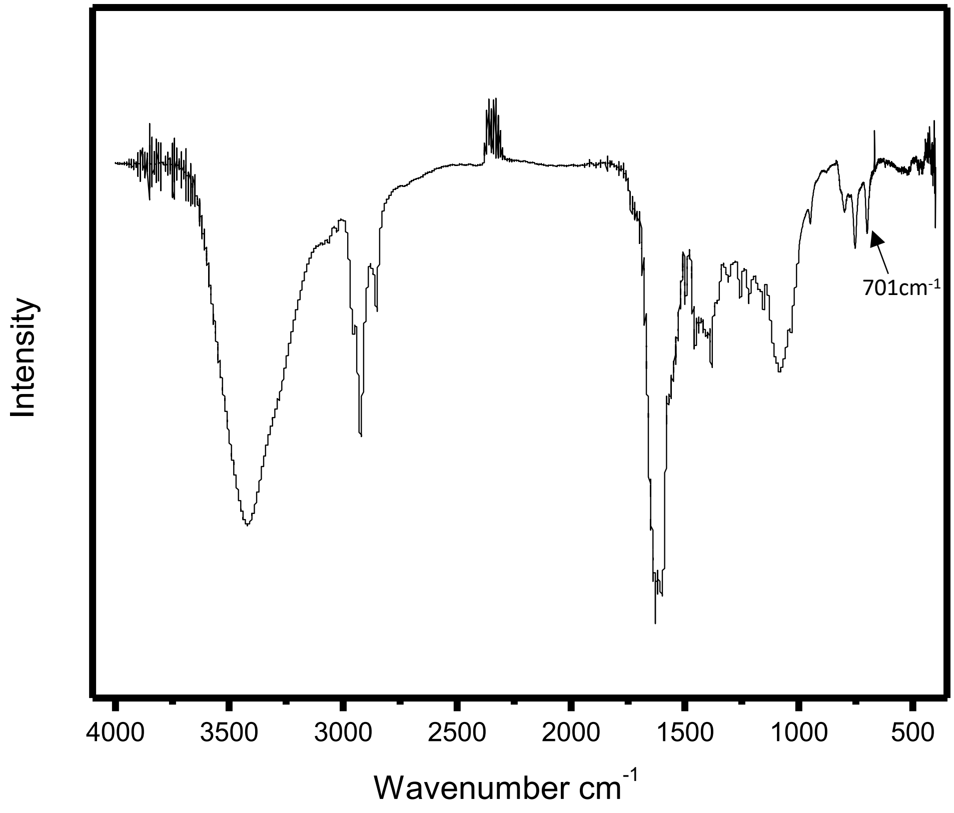

Figure S6. FT-IR spectrum of fatuamide A.

A.

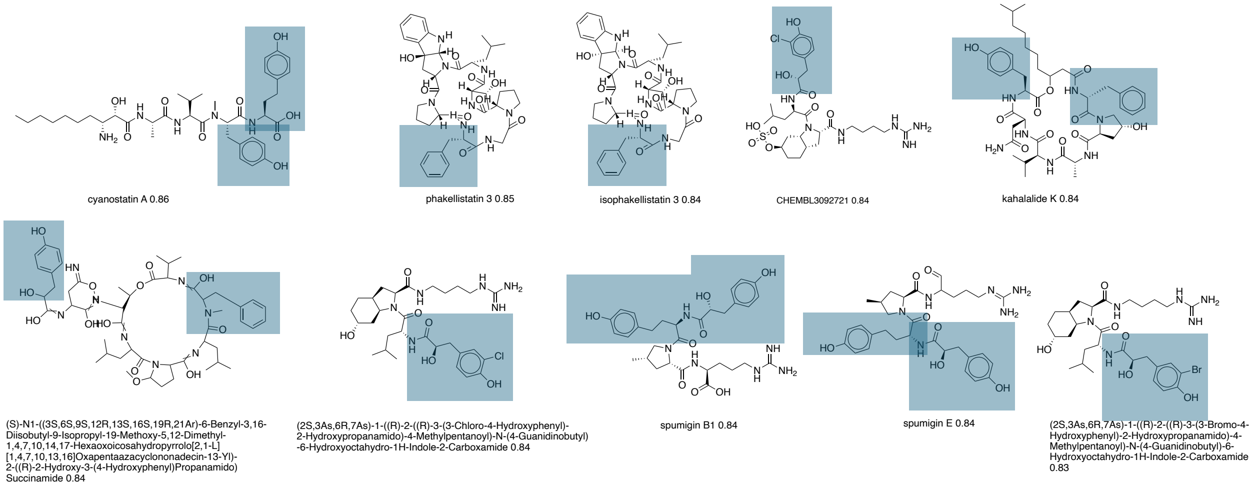

B.

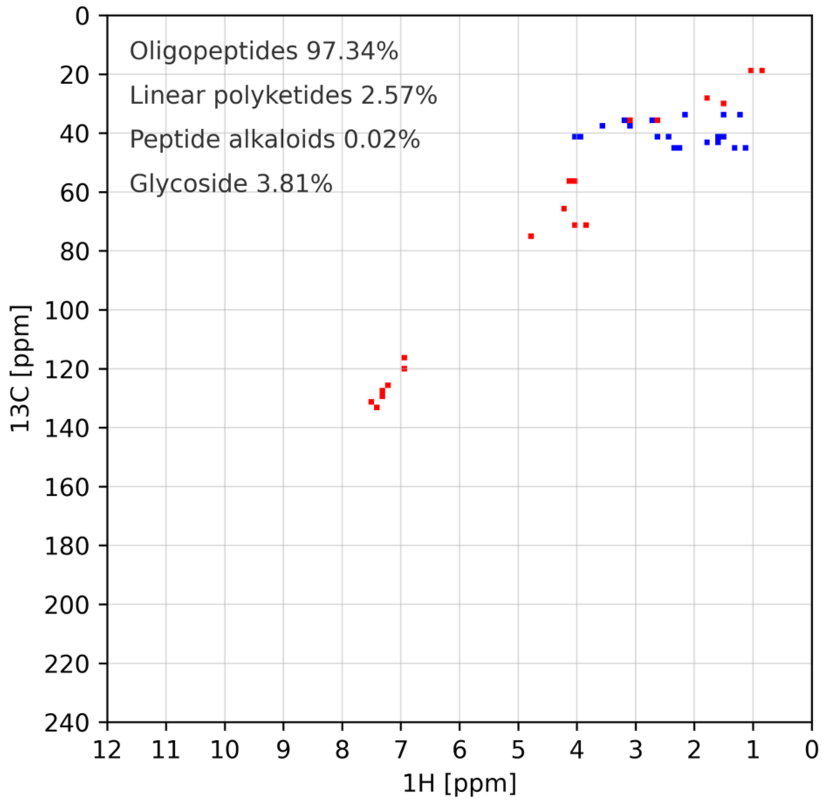

Figure S7: A. Fatuamide A top 10 SMART 2.1 results based on cosine score. The colored boxes highlight the substructures that have similar motifs to those found in fatuamide A. **B.** Depiction of the HSQC spectrum of fatuamide A (**1**) with analysis from DeepSAT as to most likely compound class.

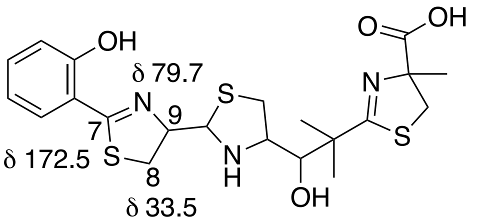

yersiniabactin Isomer I - DMF

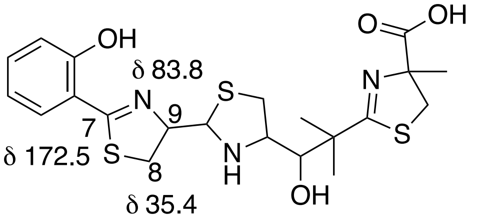

yersiniabactin Isomer II - DMF

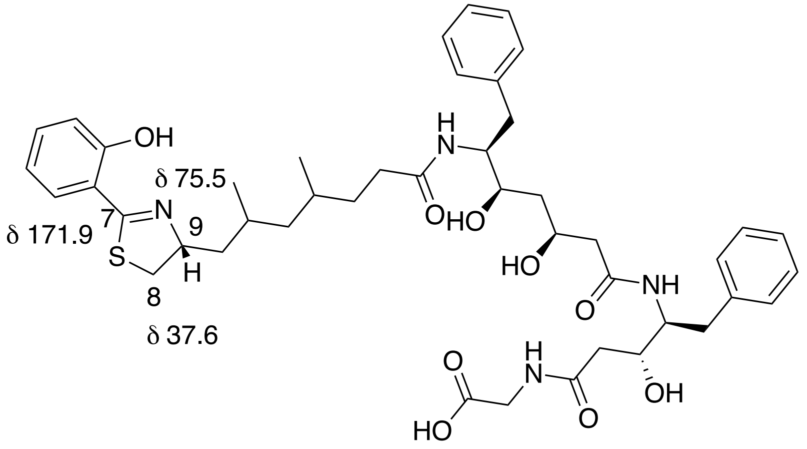

fatuamide A- MeOH-d4

Figure S8: Comparison ^{13}C NMR shifts for yersiniabactin Isomer I and Isomer II and fatuamide thiazoline ring carbon atoms**.** In the two isomers of yersiniabactin, the methylene group has shifts of δ_c 33.5 and 35.4 in DMF[3], while fatuamide A has a shift of δ_c 37.6 in MeOH-d_4. The C9 carbon of the thiazoline ring in the yersiniabactin isomers has shifts of δ_c 79.7 and 83.8 in DMF, while fatuamide A has a shift of δ_c 75.5 in MeOH-d_4. The C7 carbon of the thiazoline ring in both isomers of yersiniabactin has a shift of δ_c 172.5 in DMF, while fatuamide A has a shift of δ_c 171.9 in MeOH-d_4.

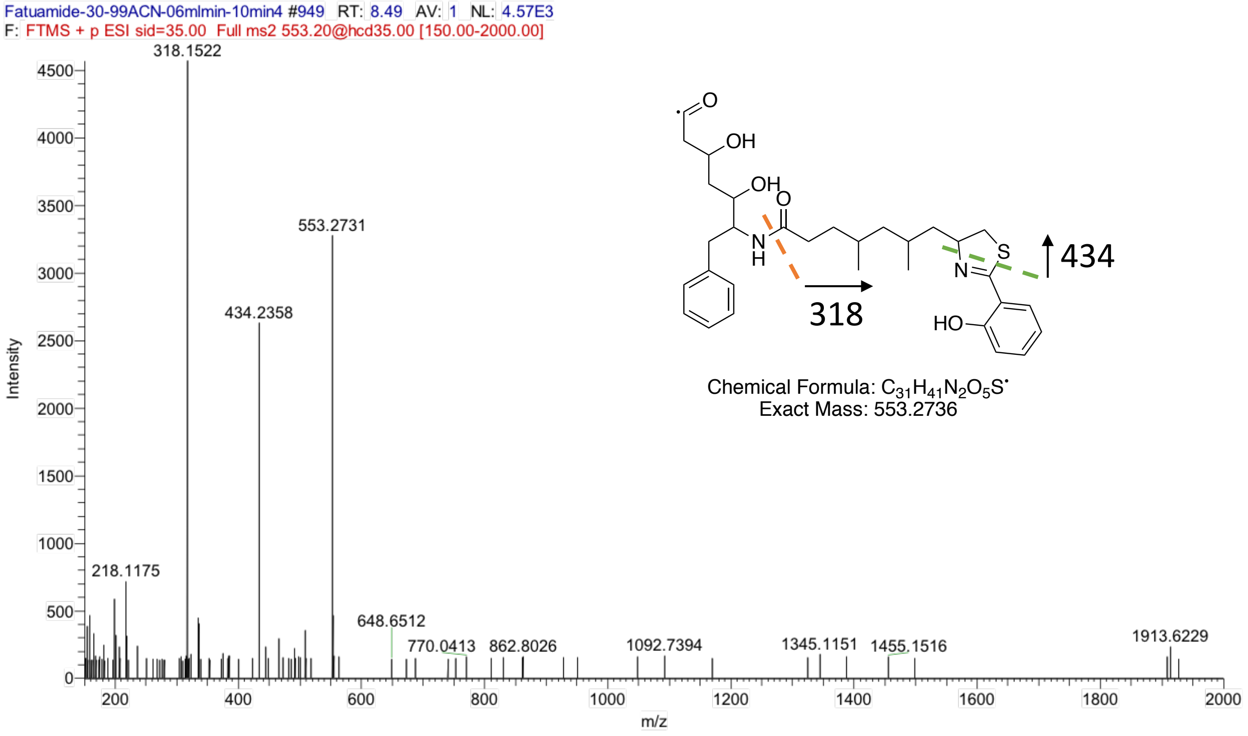

Figure S9. MS/MS of *m/z* 553 fragment of fatuamide A.

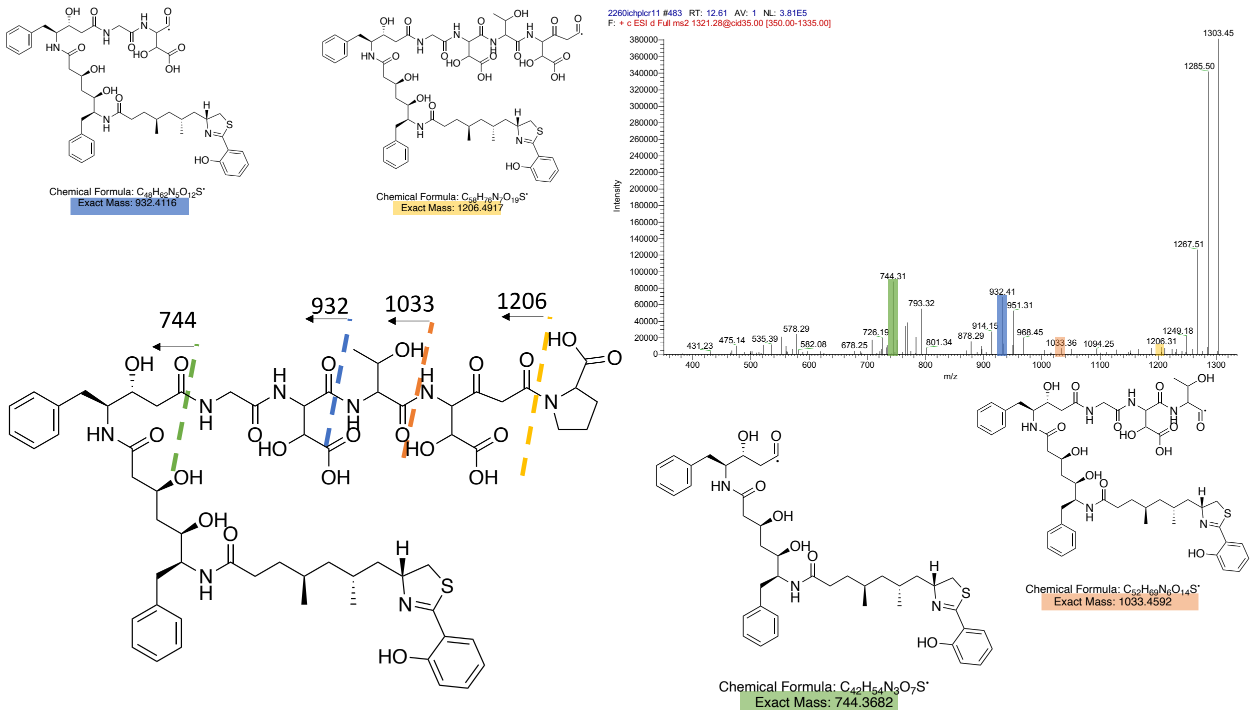

Figure S10. MS/MS analysis and proposed structure of fatuamide B. The MS/MS showed a fragment at *m/z* 744.31, corresponding to cleavage of the C42 amide bond, similar to fatuamide A. The MS/MS showed fragments at *m/z* 932.41 (corresponding to fatuamide A plus a proposed β-OH aspartic acid), *m/z* 1033.36 (corresponding to fatuamide A plus a proposed β-OH aspartic acid and threonine), and *m/z* 1206.31 (corresponding to fatuamide A plus a proposed β-OH aspartic acid, threonine, β-OH aspartic acid and a ketide extension). Finally, the molecular ion at *m/z* 1321 suggests the presence of a terminal proline that is final product released from the biosynthetic megaenzyme. NMR spectra for fatuamide B are available at https://np-mrd.org/natural_products/NP0333789.

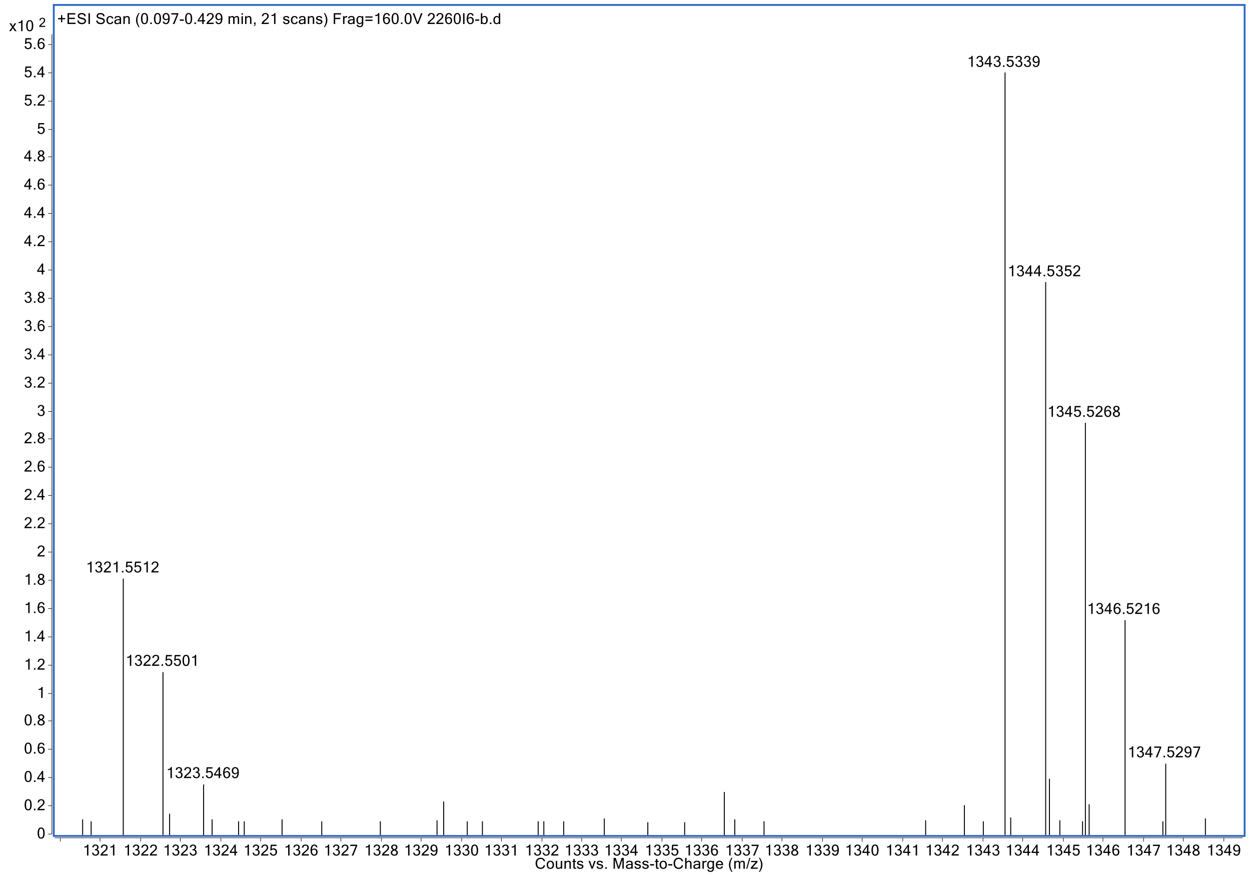

Figure S11. HR-ESI-TOFMS of fatuamide B $[M+H]^+$ 1321.5512.

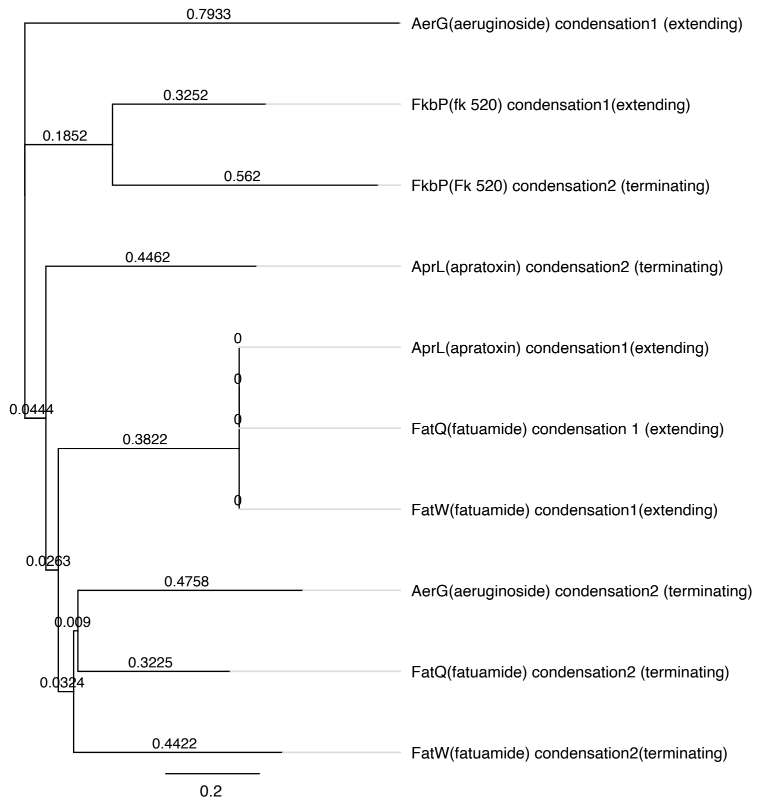

Figure S12. Phylogenetic tree of cyanobacterial condensation units. The tree was built with Geneious Tree Builder with a global alignment with free end gaps with a Blosum62 cost matrix using a Jukes-Cantor genetic distance model with a neighbor-joining tree building method.

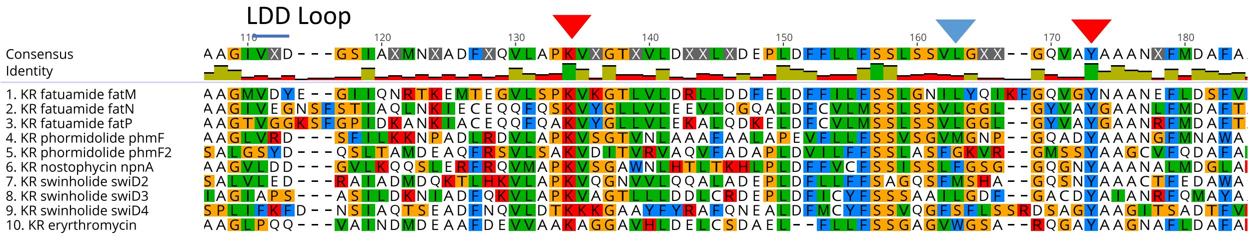

Figure S13. Alignment of different KR domains from cyanobacterial natural products made with Geneious version 2019.2 created by Biomatters. Position of the conserved tryptophan (W) for A-type KRs is denoted with a blue triangle. The LDD Loop is denoted by a blue line in the upper left corner. Red triangles denote catalytic sites.

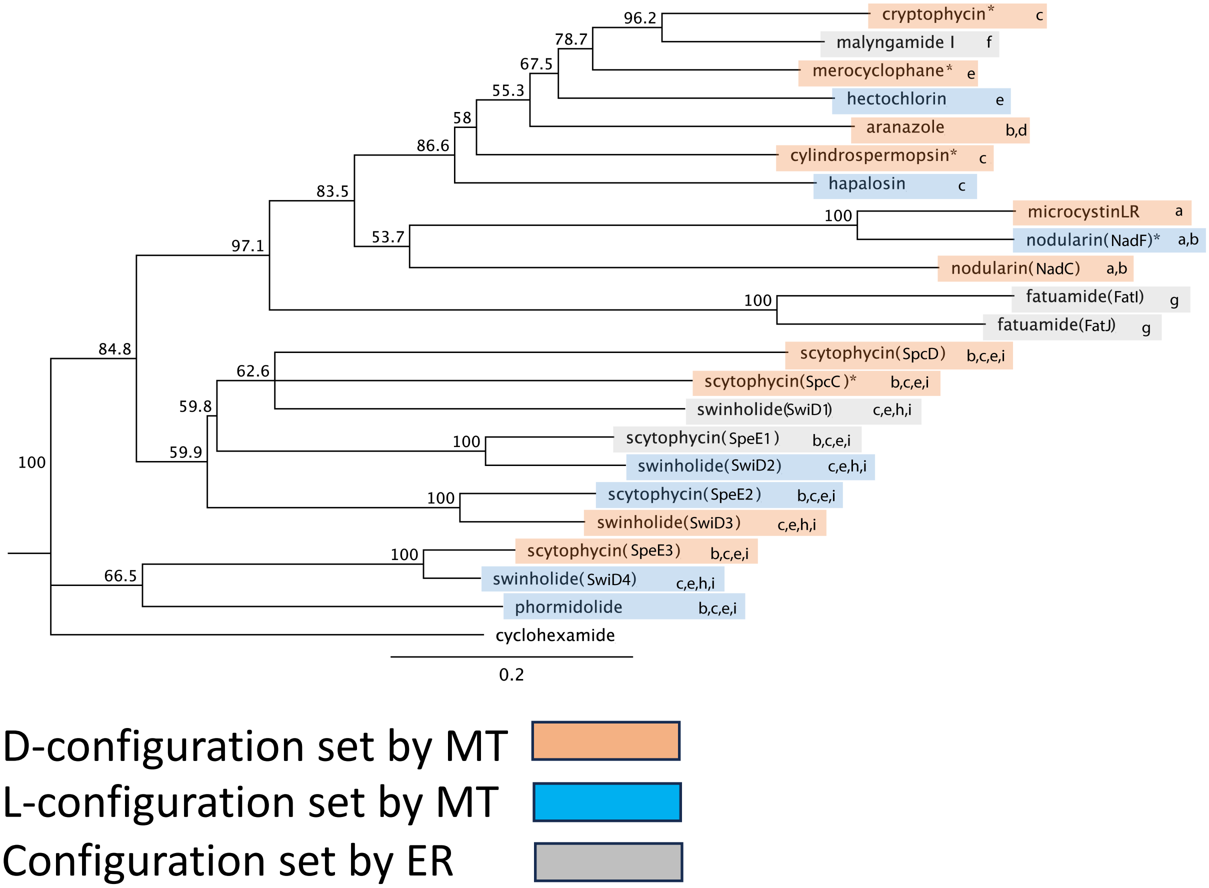

Figure S14. Phylogenetic tree of cyanobacterial C-methyl transferases (cMT). Using Geneious, a multiple sequence alignment was created using Clustal Omega and sequences were grouped by similarity. The Geneious Tree Builder with the Jukes-Cantor Genetic Distance Model and Neighbor-Joining tree building method with 1,000 bootstraps was used to create the consensus tree with a support threshold of 50%. Based on the oxidation state of the upstream carbon in the biosynthetic process, either a MT or an ER is expected to set the configuration of the methyl branch. Those associated with a D-configuration due to the MT present are color-coded in orange, those associated with an L-configuration due to the MT present are color-coded in blue, and those whose stereochemical configuration set by an ER are color-coded in grey (e.g. the ER of fatJ and malyngamide I sets the methyl branch as D whereas the ER of FatI, speE1 and swiD1 sets the methyl branch as L). An asterisk indicates uncertainty due to unknown stereospecificity of other reactions impacting the configuration of the methyl branch. The method by which the configuration of the methyl group was determined is indicated by the subscripted letters, as follows: a: Chemical degradation compared with standards. b: NMR. c: Total synthesis. d: Bioinformatics. e: X-ray crystallography. f: X-ray crystallography of a fragment. g: DFT calculations and NMR. h: Mosher's ester method. i: Circular dichroism. The outgroup used for the tree is the cMT from the cycloheximide pathway in the genome assembly of *Streptomyces* sp. YIM 56141 (BGC0000175.5 in the MIBiG repository).[4]

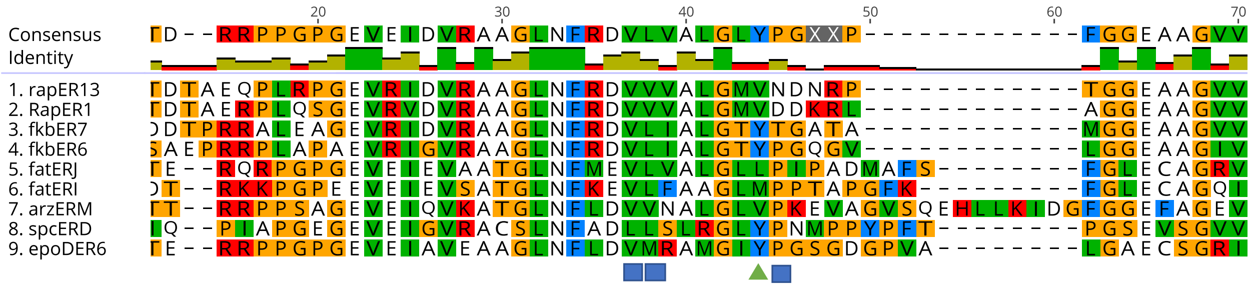

Figure S15. Alignment of cyanobacterial ER domains using Geneious. The green triangle at bottom represents the site for stereochemical control according to Kwan *et al.*[5] A tyrosine (Y) residue at this position gives a 2*S* configuration for the resulting secondary methyl group. A valine (V), alanine (A), or phenylalanine (F) residue at this position gives 2*R* configuration. The blue boxes denote additional sites that are involved in stereochemical control of the resulting methyl group.

(4*S*,6*S*,8*R*)-9-tert-butoxy-4,6,8-trimethyl-9-oxononanoic acid

anti

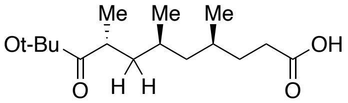

0.15

(2*S*,3*R*,4*S*,6*R*)-2,4,6-trimethyloct-7-ene-1,3 diol

syn

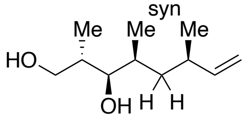

0.14

(2*S*,4*S*)-5-acetoxy-2,4-dimethylpentanoic acid

anti

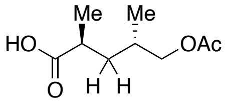

0.15

tert-butyl((4*R*,6*R*,*E*)-4.6-dimethylocta-2,7-dienyloxy)diphenylsilane

syn

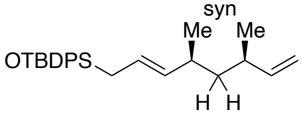

0.16

(2*R*,4*S*,6*S*)-tert-butyl-2,4,6-trimethyl-8-phenyloctanoate

anti

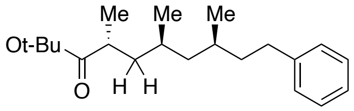

0.13

(4*S*.6*R*,*E*)-4,6-dimehtylocta-2.7-dien-1-ol

syn

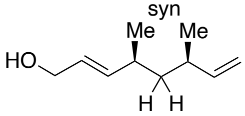

0.17

fatuamide A

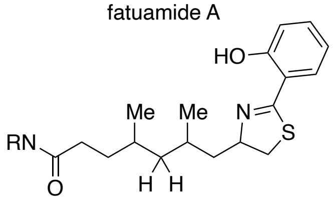

0.14

Figure S16. Analysis of ^{1}H NMR chemical shifts of different stereoisomers of 1,3-dimethyl substituted compounds compared to those in fatuamide A. [6,7,8,9]

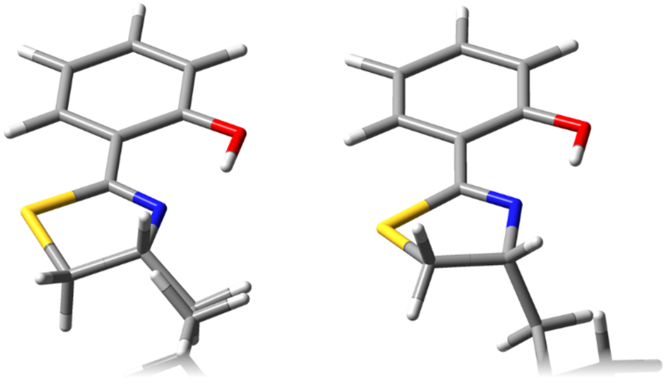

Figure S17. (A) The $_{8}T^{9}$ (left) and $^{8}T_{9}$ (right) conformations of the phenylthiazoline in fatuamide A (**1**).

1	Functional			Solvent?		Basis Set?		Type of Data	
2	mPW1PW91			PCM		6-311+G(d,p)		Shielding Tensors	
3									
12				DP4+	0.00%	87.77%	12.23%	0.00%	-
14		Nuclei	sp2?	Experimental	*RR* -1m	*RS* -1m	*SR* -1m	*SS* -1m	Isomer 5
15	C-1	C	x	160.2	21.19	20.67	21.3	21.2	
16	C-2	C	x	117.8	65.48	65.37	65.6	65.5	
17	C-3	C	x	134.0	47.63	47.80	47.6	47.7	
18	C-4	C	x	120.0	63.52	64.02	63.6	63.7	
19	C-5	C	x	131.6	50.26	50.20	50.1	50.1	
20	C-6	C	x	117.6	66.48	66.63	66.5	66.5	
21	C-9	C		75.5	109.85	109.31	108.8	109.3	
22	C-10	C		44.3	141.98	141.41	142.34	141.02	
23	C-11	C		29.3	155.39	155.74	154.91	154.87	
24	C-12	C		20.3	167.22	168.42	167.33	168.67	
25	C-13	C		45.1	144.21	142.05	141.11	141.16	
26	C-14	C		31.1	154.56	153.77	153.51	154.30	
27	C-15	C		19.4	166.85	168.89	167.34	166.64	
28	C-16	C		35.0	154.45	151.53	151.11	154.07	
29	C-17	C		35.0	151.60	149.79	148.35	151.10	
30	H-2	H		6.93	24.38	24.46	24.41	24.41	
31	H-3	H		7.35	24.00	24.03	24.01	24.01	
32	H-4	H		6.89	24.53	24.58	24.54	24.54	
33	H-5	H		7.42	23.97	23.99	23.96	23.98	
34	H-8a	H		3.02	28.75	28.71	28.74	28.75	
35	H-8b	H		3.52	28.24	28.26	28.26	28.23	
36	H-9	H		4.76	26.83	26.82	26.88	26.83	
37	H-10a	H		1.59	30.20	30.18	30.36	30.33	
38	H-10b	H		1.69	29.99	29.99	29.97	30.12	
39	H-11	H		1.78	29.82	29.85	29.75	29.74	
40	H-13a	H		1.11	30.79	30.60	30.65	30.64	
41	H-13b	H		1.25	30.26	30.52	30.64	30.58	
42	H-14	H		1.47	30.00	30.19	30.30	30.05	
43	H-16a	H		1.20	30.29	30.35	30.47	30.27	
44	H-16b	H		1.41	30.03	30.19	30.12	30.08	
45	H-17a	H		2.07	29.69	29.60	29.68	29.68	
46	H-17b	H		2.07	29.71	29.66	29.65	29.71	
47	H3-12	H		0.94	30.80	30.83	30.76	30.74	
48	H3-15	H		0.84	30.84	30.90	30.88	30.85	

1	Functional	Solvent?		Basis Set?		Type of Data	
2	mPW1PW91	PCM		6-311+G(d,p)		Shielding Tensors	
3							
4		*RR* -1m	*RS* -1m	*SR* -1m	*SS* -1m	Isomer 5	Isomer 6
5	sDP4+ (H data)	0.01%	98.78%	1.18%	0.02%	-	-
6	sDP4+ (C data)	14.54%	34.88%	30.52%	20.06%	-	-
7	sDP4+ (all data)	0.01%	98.95%	1.04%	0.01%	-	-
8	uDP4+ (H data)	0.54%	74.69%	21.84%	2.93%	-	-
9	uDP4+ (C data)	0.05%	2.11%	96.12%	1.72%	-	-
10	uDP4+ (all data)	0.00%	6.97%	92.81%	0.22%	-	-
11	DP4+ (H data)	0.00%	99.65%	0.35%	0.00%	-	-
12	DP4+ (C data)	0.02%	2.42%	96.43%	1.13%	-	-
13	DP4+ (all data)	0.00%	87.77%	12.23%	0.00%	-	-

Figure S18. The results of DP4+ statistical analysis of ^{1}H and ^{13}C NMR chemical shifts predicted for *RR*-**1m**, *RS*-**1m**, *SR*-**1m**, and *SS*-**1m** at the mPW1PW91/6-311+G(d,p)/PCM level of theory. Conformational ensembles were calculated at the B3LYP/6-311G+(d,p)/PCM//B3LYP/6-31G(d) level of theory (for energies and geometries, respectively).

1	**Functional**			**Solvent?**		**Basis Set?**		**Type of Data**	
2	**mPW1PW91**			**PCM**		**6-311+G(d,p)**		**Shielding Tensors**	
3									
12				**DP4+**	**97.63%**	**2.37%**			**-**
14		**Nuclei**	**sp2?**	**Experimental**	***RS*-1m**	***SR*-1m**	**Isomer 3**	**Isomer 4**	**Isomer 5**
15	C-1	C	x	160.2	20.87	21.05			
16	C-2	C	x	117.8	66.08	66.11			
17	C-3	C	x	134.0	48.00	47.97			
18	C-4	C	x	120.0	64.11	63.98			
19	C-5	C	x	131.6	50.65	50.62			
20	C-6	C	x	117.6	66.77	66.72			
21	C-9	C		75.5	109.55	109.74			
22	C-10	C		44.3	142.20	142.31			
23	C-11	C		29.3	156.53	156.01			
24	C-12	C		20.3	169.26	168.90			
25	C-13	C		45.1	141.91	142.02			
26	C-14	C		31.1	154.57	154.37			
27	C-15	C		19.4	169.35	168.94			
28	C-16	C		35.0	150.46	150.34			
29	C-17	C		35.0	150.39	150.08			
30	H-2	H		6.93	24.49	24.48			
31	H-3	H		7.35	24.07	24.06			
32	H-4	H		6.89	24.62	24.61			
33	H-5	H		7.42	24.05	24.04			
34	H-8a	H		3.02	28.80	28.80			
35	H-8b	H		3.52	28.32	28.34			
36	H-9	H		4.76	26.93	26.94			
37	H-10a	H		1.59	30.23	30.36			
38	H-10b	H		1.69	29.98	30.02			
39	H-11	H		1.78	30.01	29.85			
40	H-13a	H		1.11	30.67	30.68			
41	H-13b	H		1.25	30.63	30.67			
42	H-14	H		1.47	30.27	30.28			
43	H-16a	H		1.20	30.42	30.42			
44	H-16b	H		1.41	30.25	30.24			
45	H-17a	H		2.07	29.67	29.73			
46	H-17b	H		2.07	29.74	29.69			
47	H3-12	H		0.94	30.89	30.85			
48	H3-15	H		0.84	30.96	30.94			

1	**Functional**	**Solvent?**		**Basis Set?**		**Type of Data**	
2	**mPW1PW91**	**PCM**		**6-311+G(d,p)**		**Shielding Tensors**	
3							
4		***RS*-1m**	***SR*-1m**	**Isomer 3**	**Isomer 4**	**Isomer 5**	**Isomer 6**
5	**sDP4+ (H data)**	**91.30%**	**8.70%**	**-**	**-**	**-**	**-**
6	**sDP4+ (C data)**	**60.54%**	**39.46%**	**-**	**-**	**-**	**-**
7	**sDP4+ (all data)**	**94.15%**	**5.85%**	**-**	**-**	**-**	**-**
8	**uDP4+ (H data)**	**89.72%**	**10.28%**	**-**	**-**	**-**	**-**
9	**uDP4+ (C data)**	**22.70%**	**77.30%**	**-**	**-**	**-**	**-**
10	**uDP4+ (all data)**	**71.95%**	**28.05%**	**-**	**-**	**-**	**-**
11	**DP4+ (H data)**	**98.92%**	**1.08%**	**-**	**-**	**-**	**-**
12	**DP4+ (C data)**	**31.07%**	**68.93%**	**-**	**-**	**-**	**-**
13	**DP4+ (all data)**	**97.63%**	**2.37%**	**-**	**-**	**-**	**-**

Figure S19. The results of DP4+ statistical analysis of ^{1}H and ^{13}C NMR chemical shifts predicted for *RS*-**1m** and *SR*-**1m** at the mPW1PW91/6-311+G(d,p)/PCM level of theory after conformer geometries were re-optimized at the B3LYP/6-311G+(d,p)/SMD level and conformer energies were evaluated at the same level of theory.

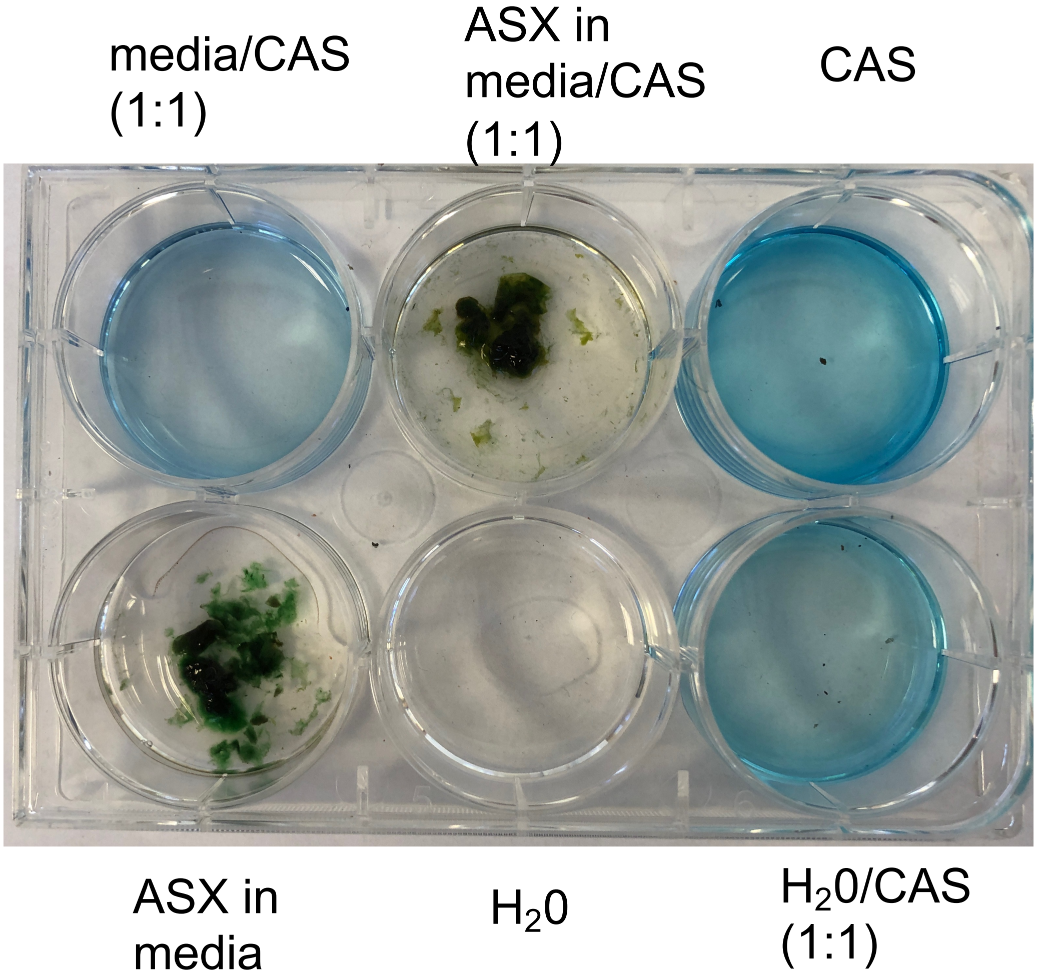

Figure S20. Chrome Azurol S (CAS) assay with fatuamide A producer ASX22JUL14-2. Loss of blue color in the ASX culture in media/CAS (1:1, top middle) indicates a positive test for siderophore activity.

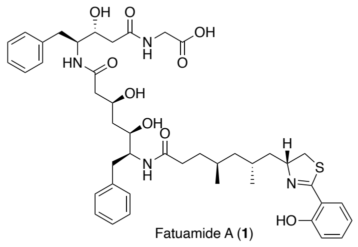

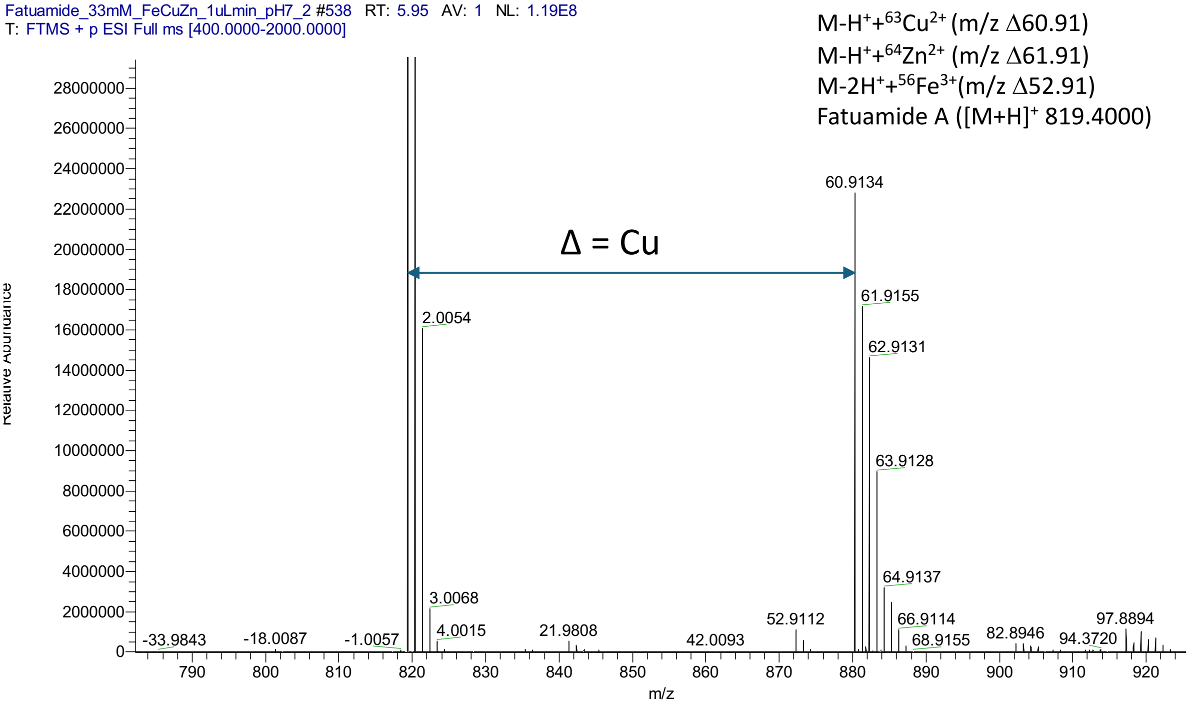

Figure S21. LC-MS/MS trace of native electrospray mass spectrometry with post column metal infusion. Mass shifts indicate proposed binding of Cu, Zn, and Fe to fatuamide A (**1**) (delta values are the differences between the [M+H] = 819.4000 minus the observed *m/z* value in each case).

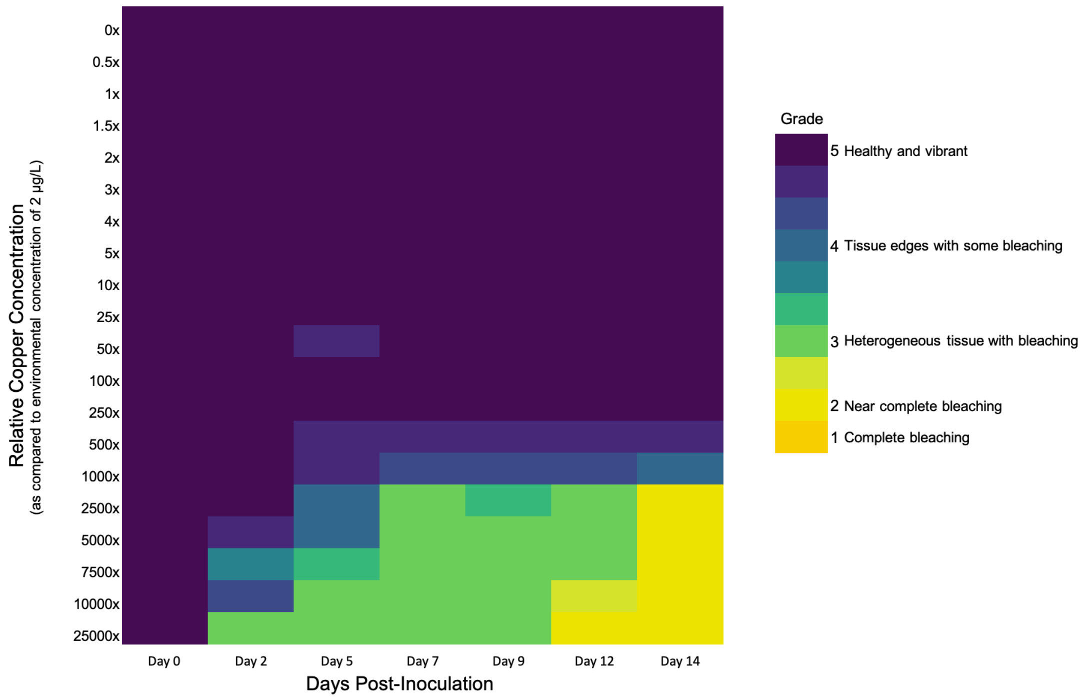

Figure S22. Heatmap with the visual grading of the health of cyanobacterial cultures over a 14-day period (x-axis) at increasing copper concentrations (y-axis).

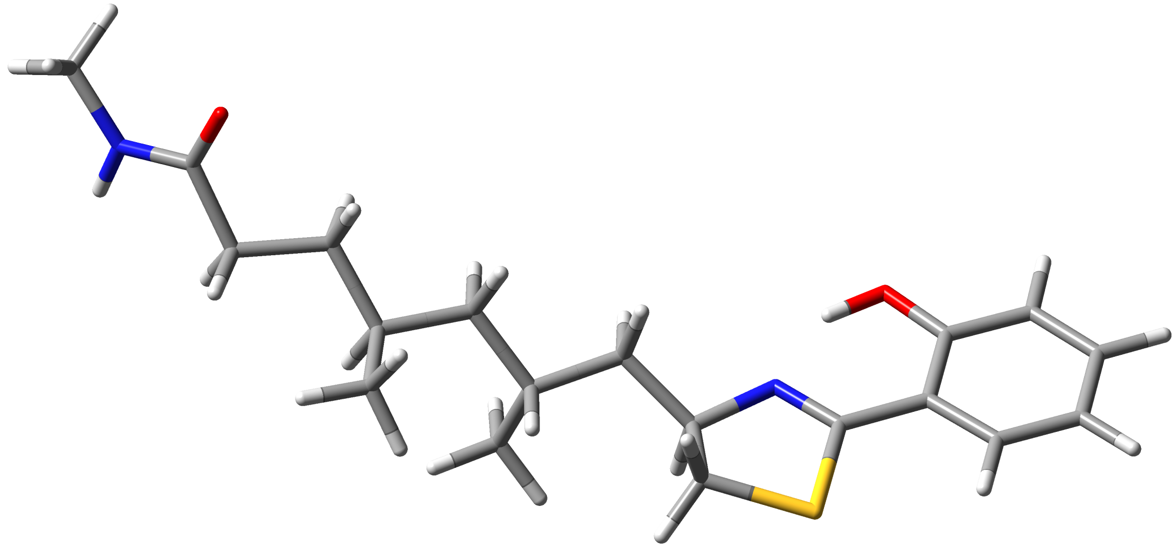

Figure S23. The lowest energy conformer of model compound *RS*-**1m** at the B3LYP/6-311G+(d,p)/SMD level of theory

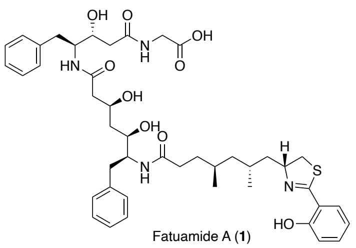

Fatuamide A (**1**)

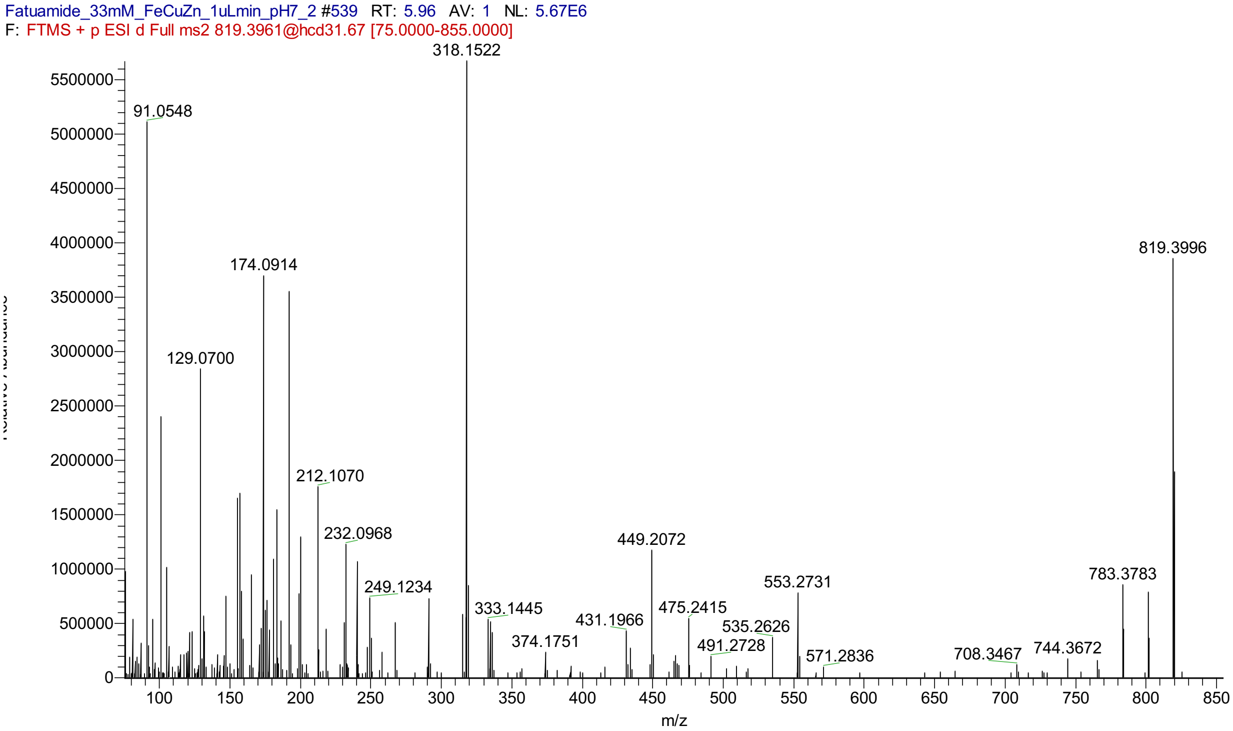

Figure S24. HR-ESI-TOFMS MS/MS of fatuamide A.

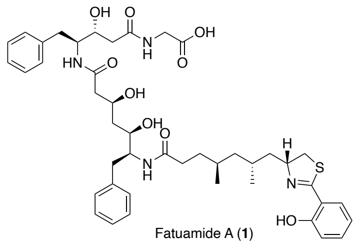

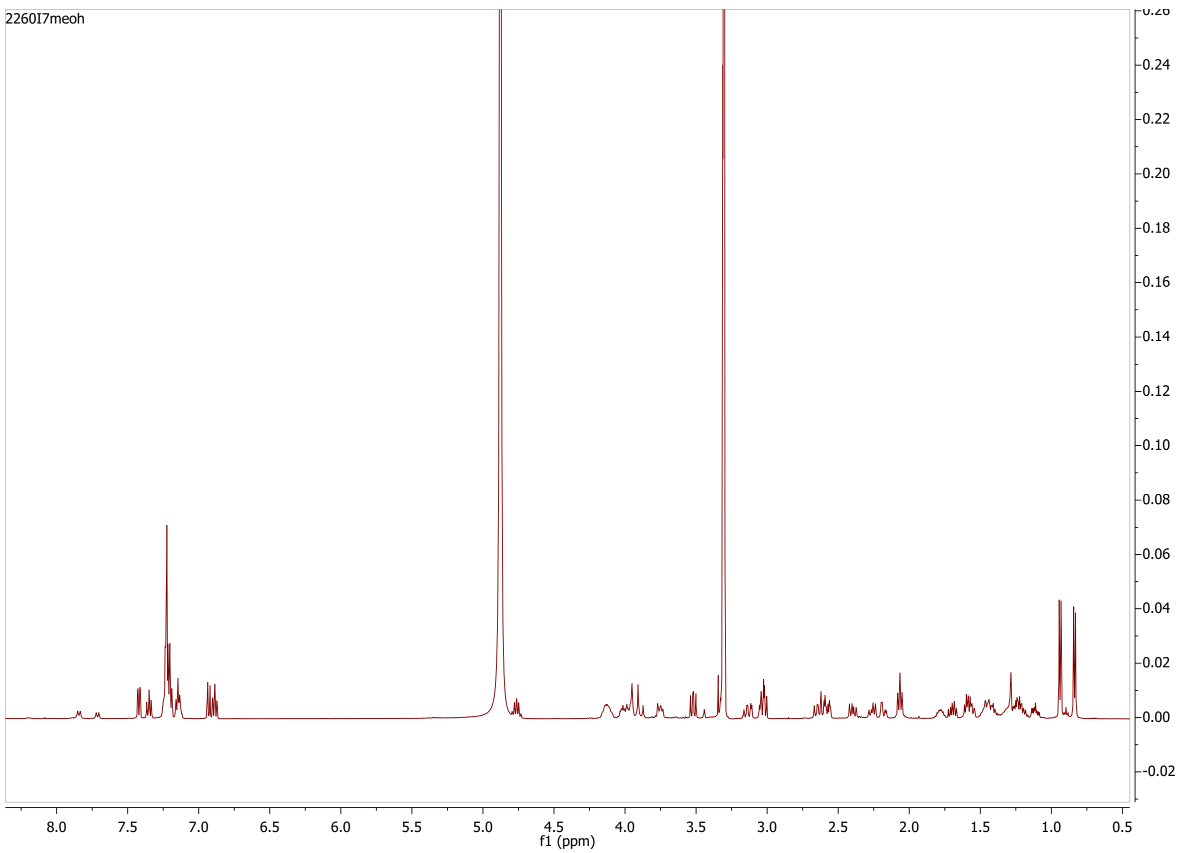

Figure S25. ^{1}H NMR spectrum of fatuamide A in MeOH-d_4 on 500 MHz JEOL instrument.

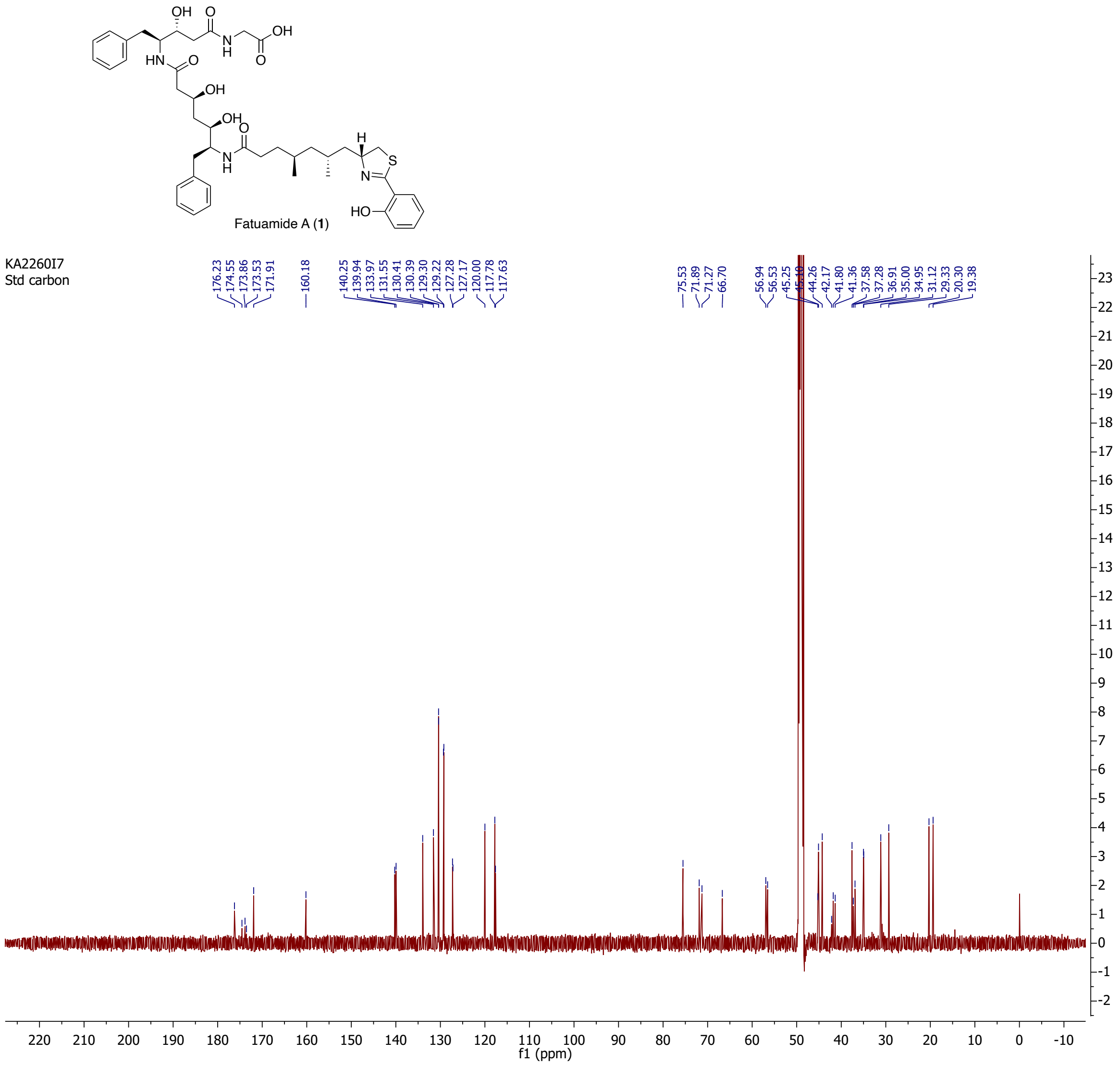

Figure S26. ^{13}C NMR spectrum of fatuamide A in MeOH-d_4 on a Varian VX 500 MHz NMR (^{13}C at 125 MHz).

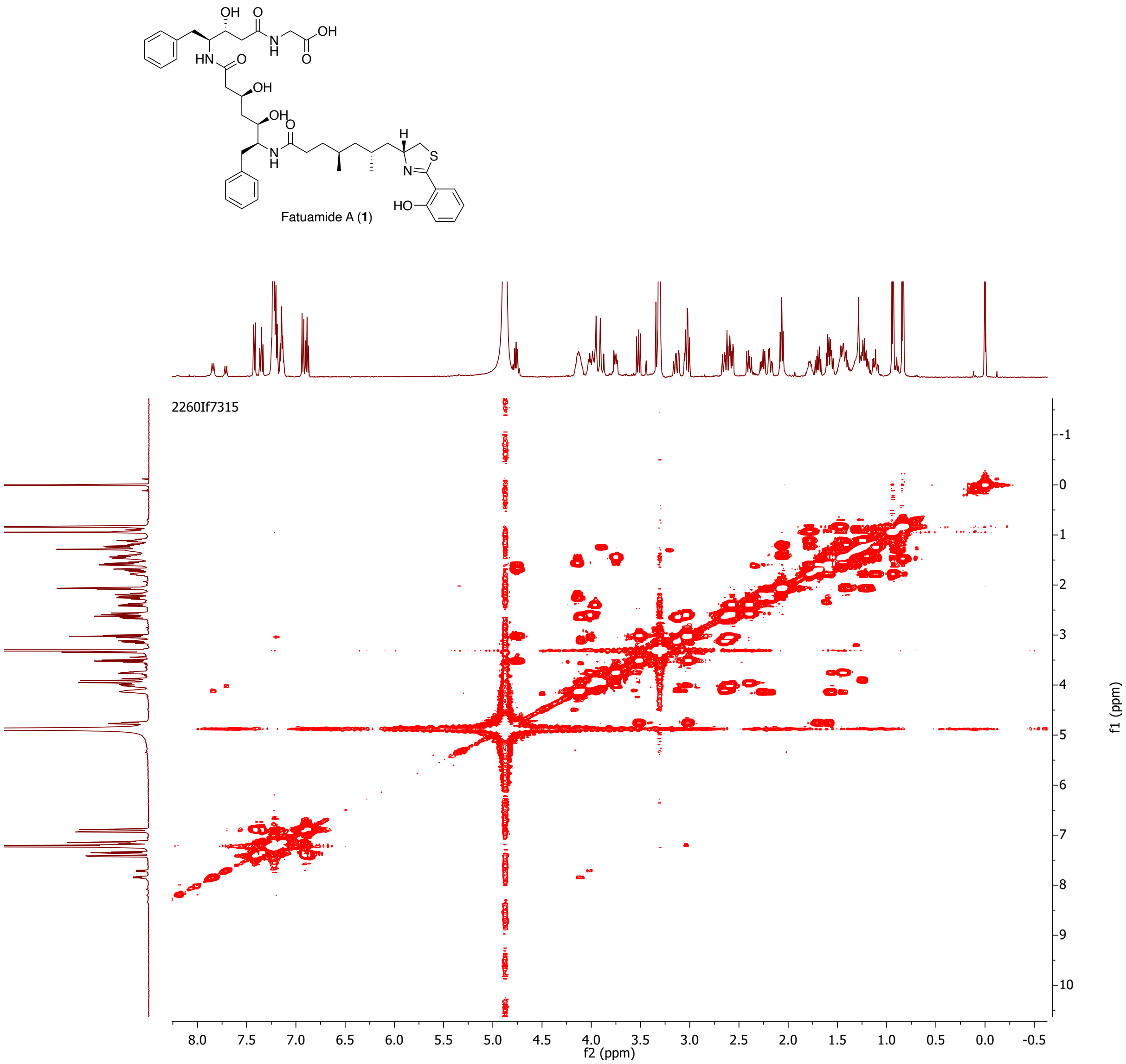

Figure S27. ^{1}H-^{1}H COSY NMR spectrum of fatuamide A in MeOH-d_4 (500 MHz).

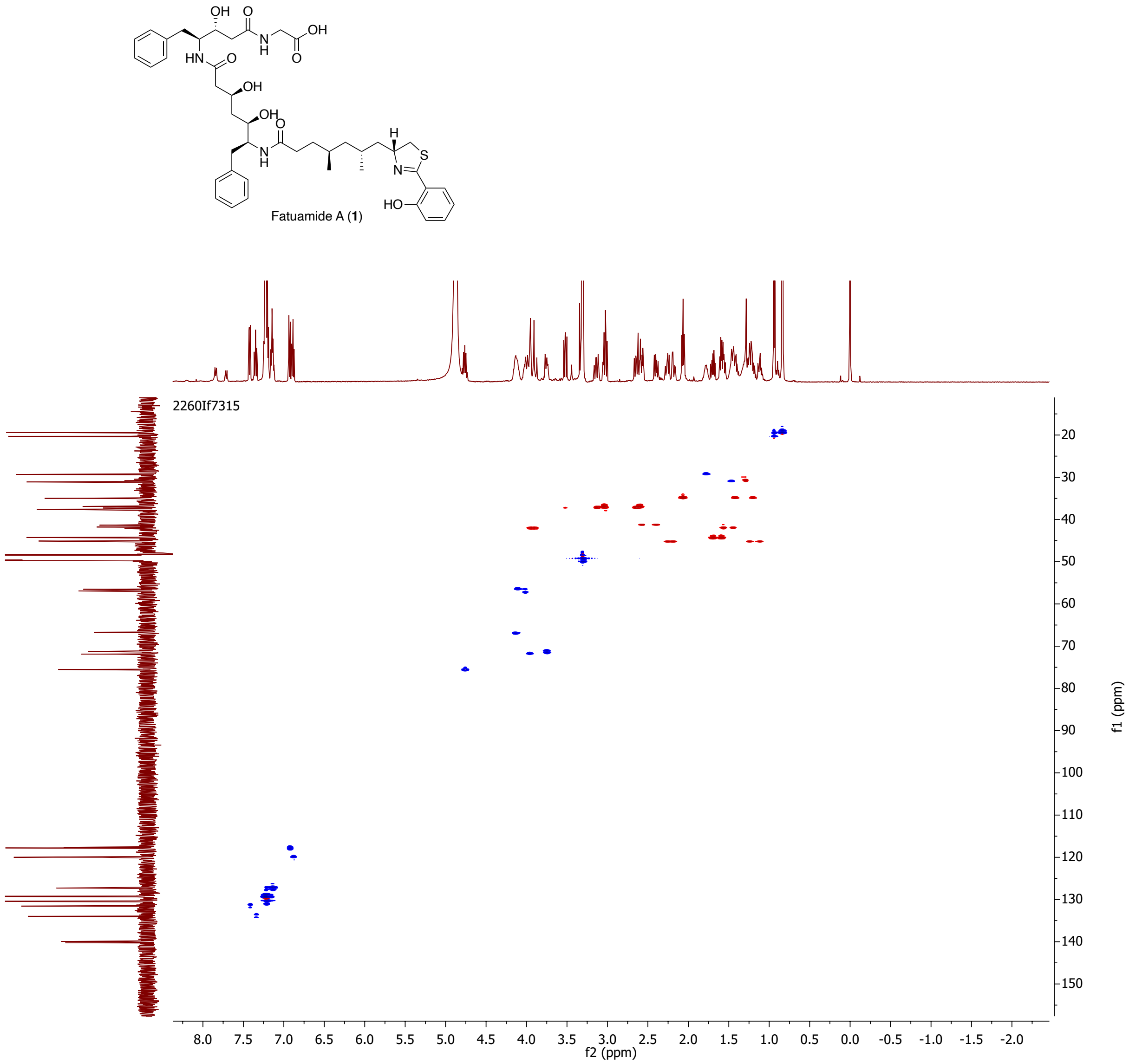

Figure S28. ^{1}H-^{13}C HSQC NMR spectrum of fatuamide A in MeOH-d_4 (500 MHz).

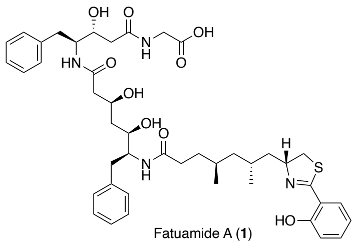

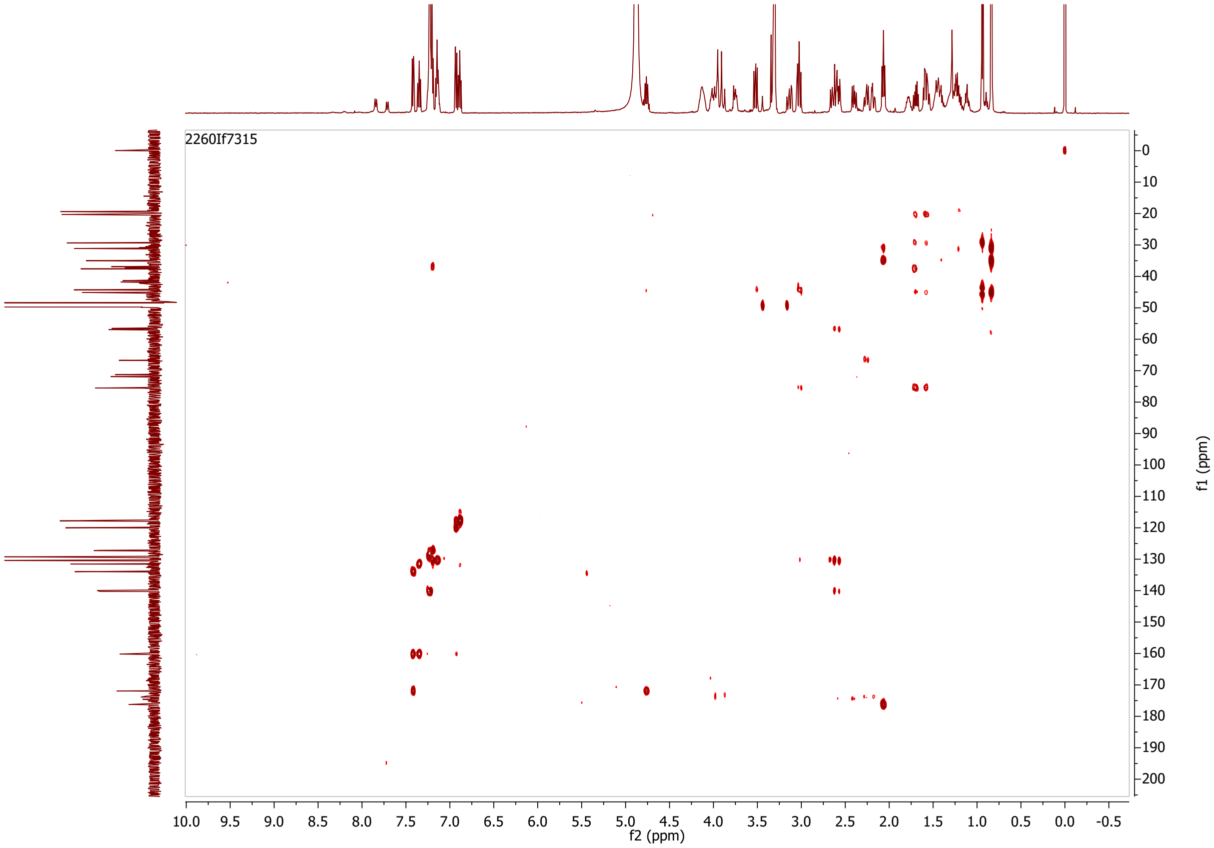

Figure S29. ^{1}H-^{13}C HMBC spectrum of fatuamide A in MeOH-d_4 (500 MHz).

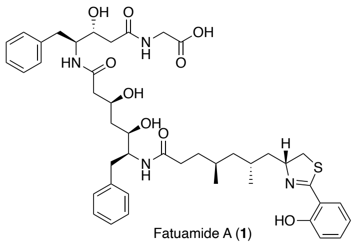

Fatuamide A (**1**)

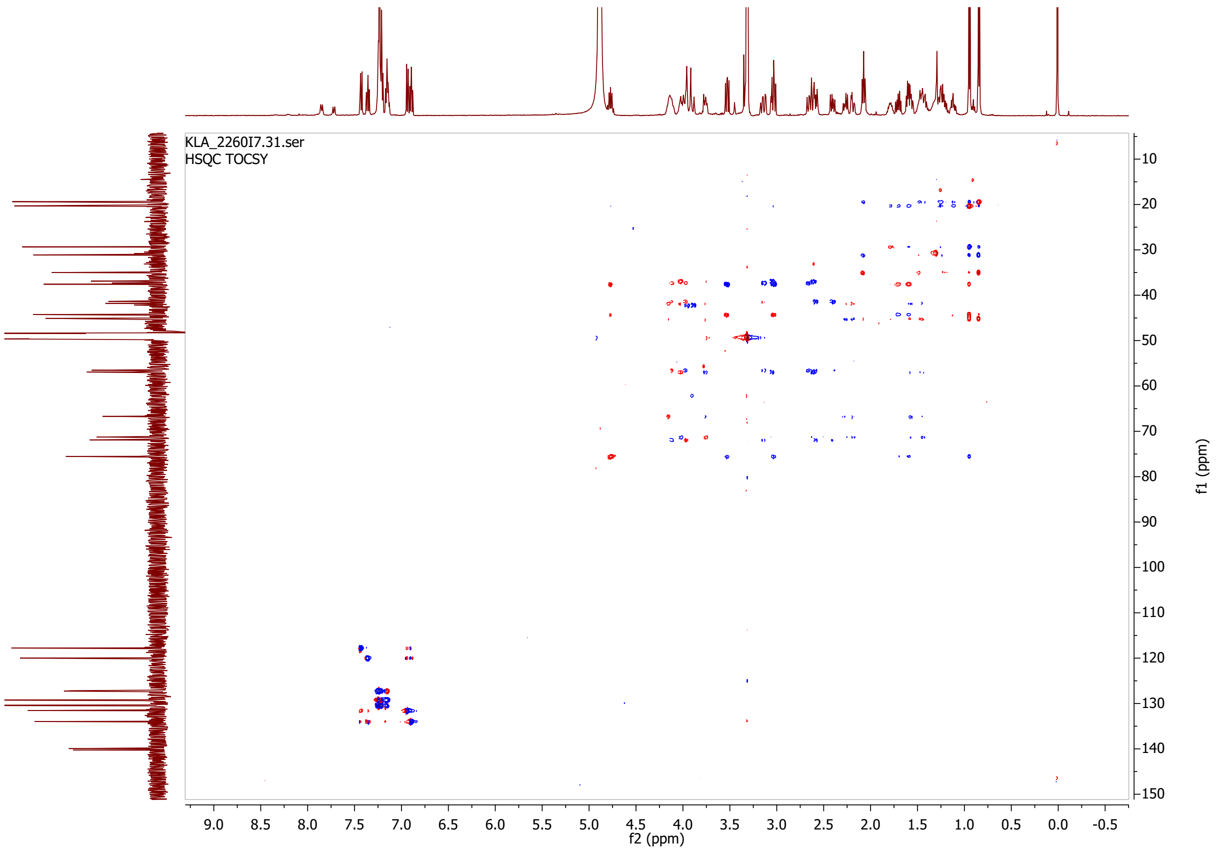

Figure S30. ^{1}H-^{13}C HSQC TOCSY spectrum of fatuamide A in MeOH-d_4 (600 MHz).

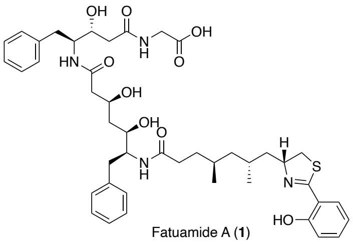

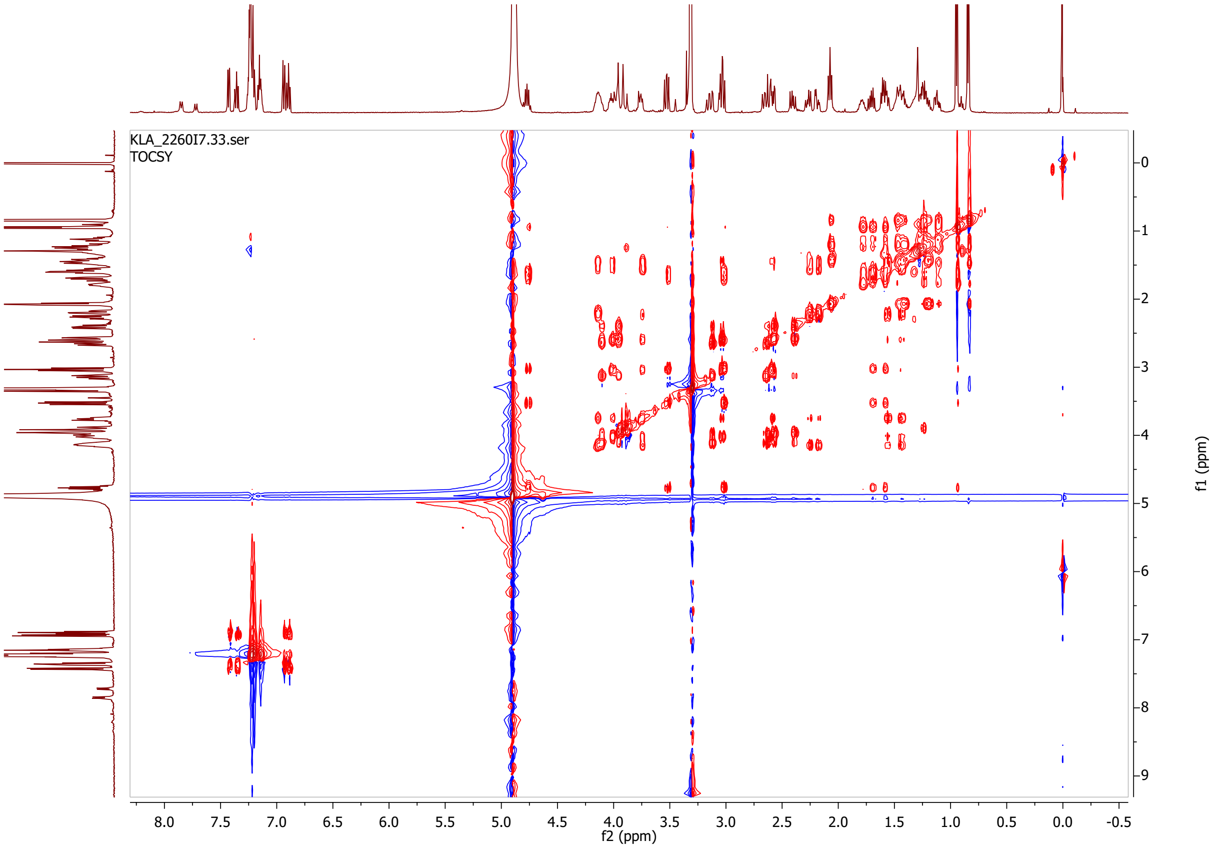

Figure S31. ^{1}H-^{1}H TOCSY spectrum of fatuamide A in MeOH-d_4 (600 MHz).

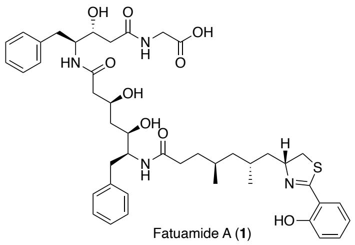

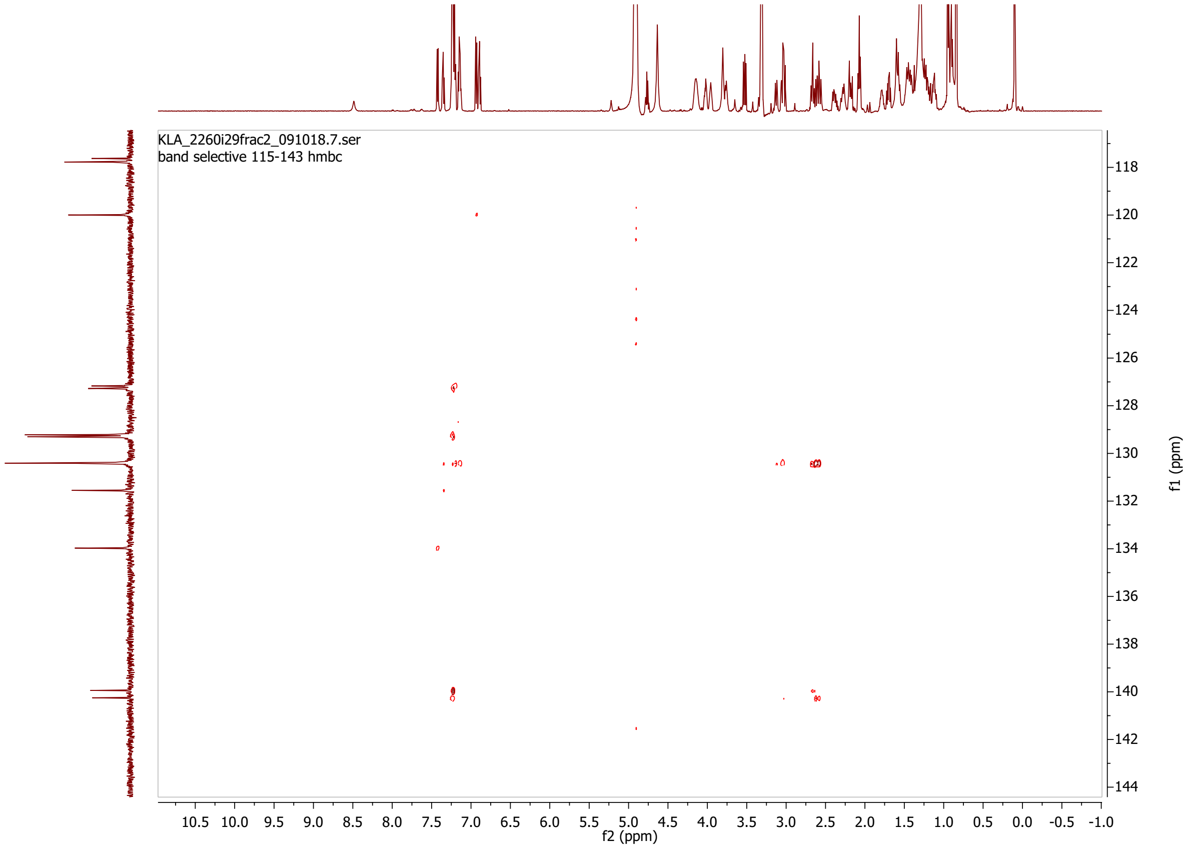

Figure S32. ^{1}H-^{13}C HMBC band selective for δ_{13C} 115-143 ppm region of fatuamide A in MeOH-d_4 (600 MHz).

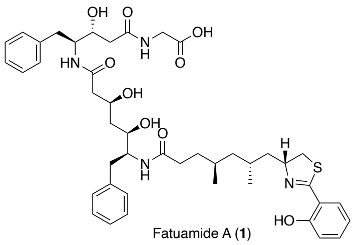

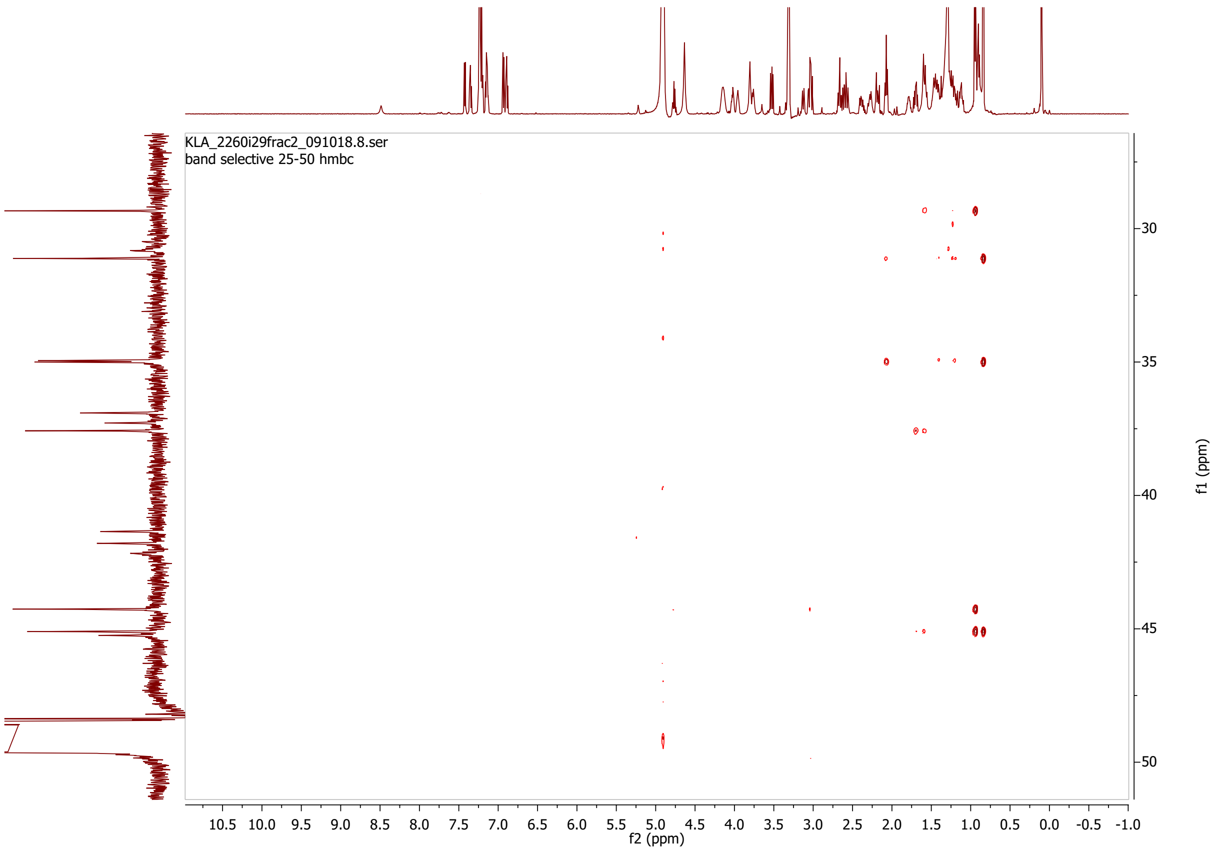

Figure S33. ^{1}H-^{13}C HMBC band selective for δ_{13C} 25-50 ppm of fatuamide A in MeOH-d_4 (600 MHz).

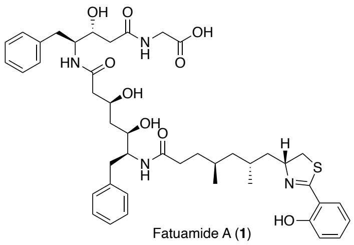

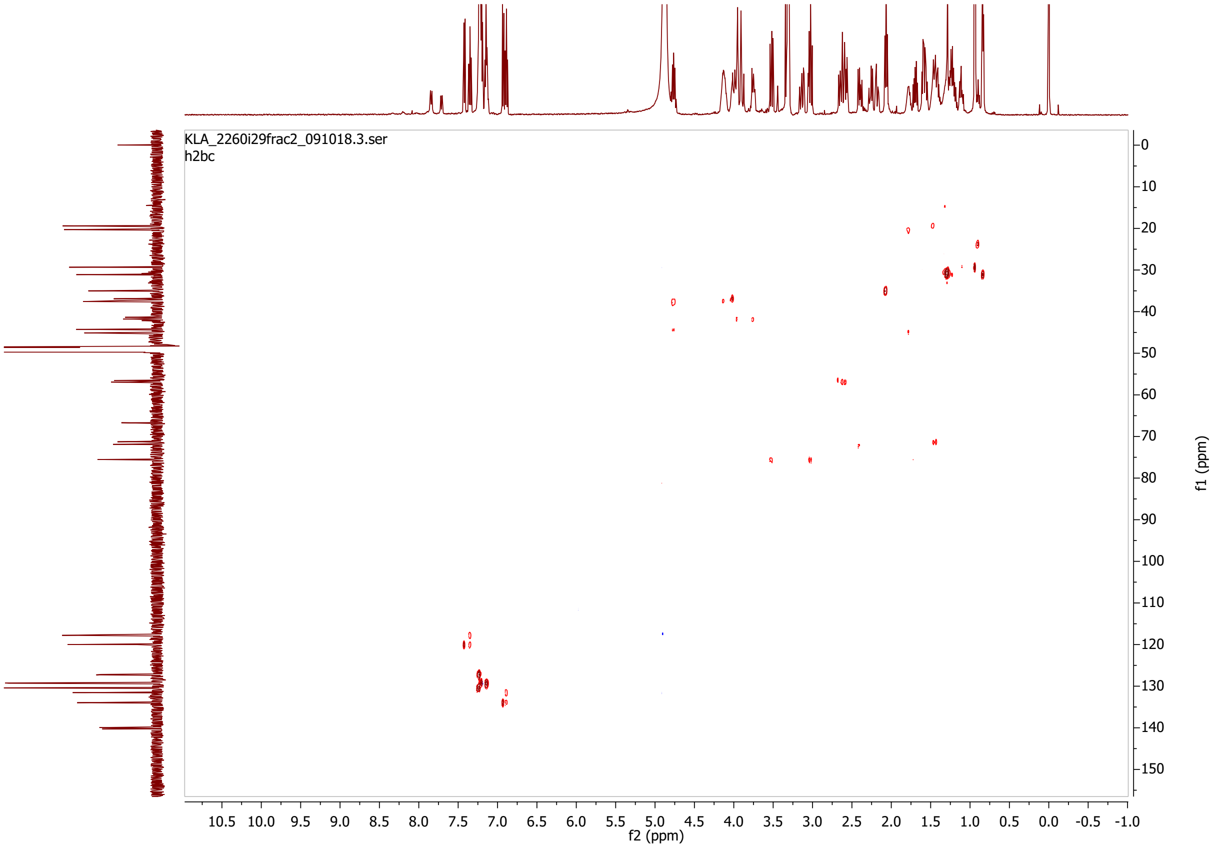

Figure S34. ^{1}H-^{13}C H2BC spectrum of fatuamide A in MeOH-d_4 (600 MHz).

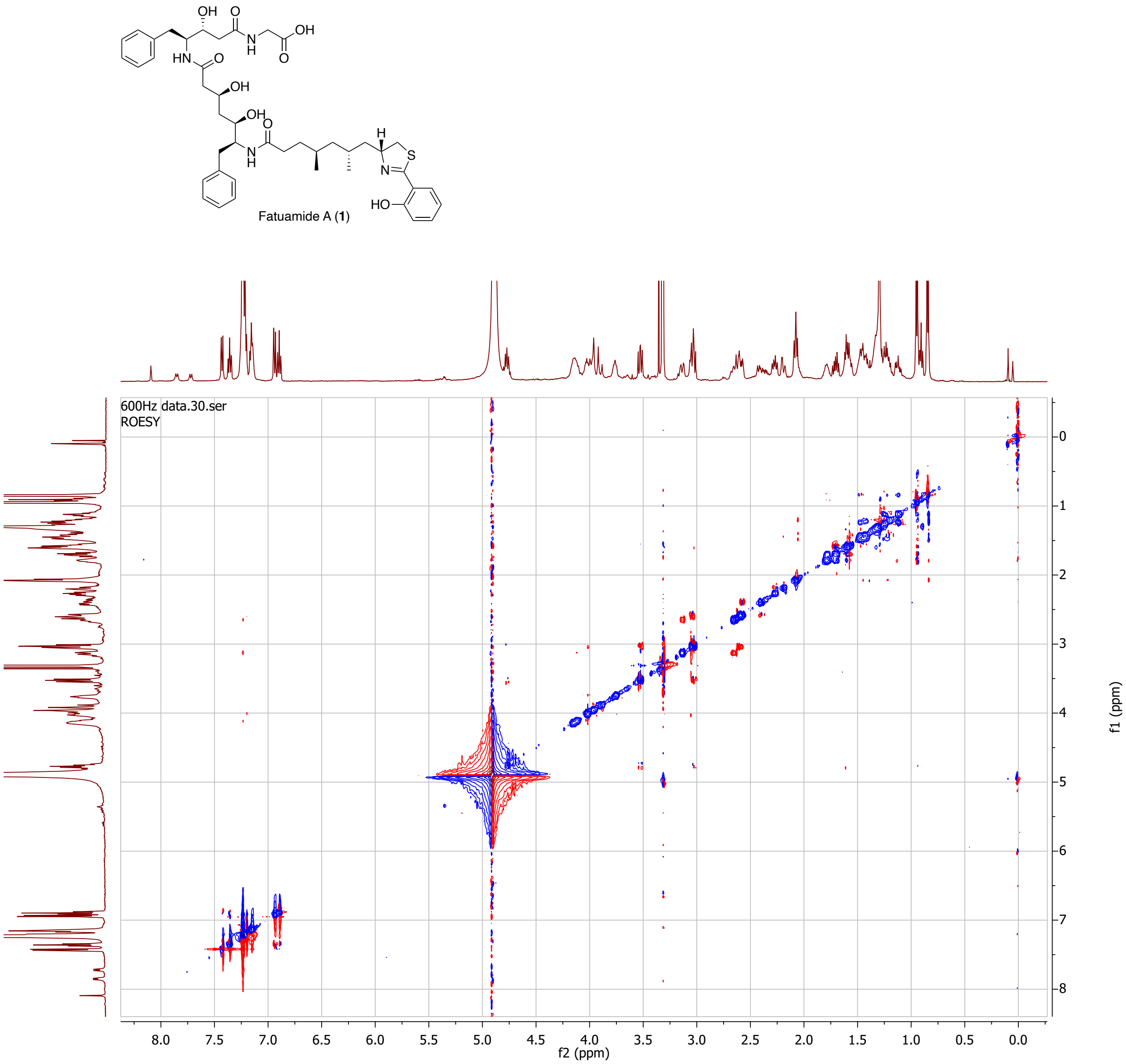

Figure S35. ^{1}H-^{1}H ROESY spectrum of fatuamide A in MeOH-d_4 (600 MHz).

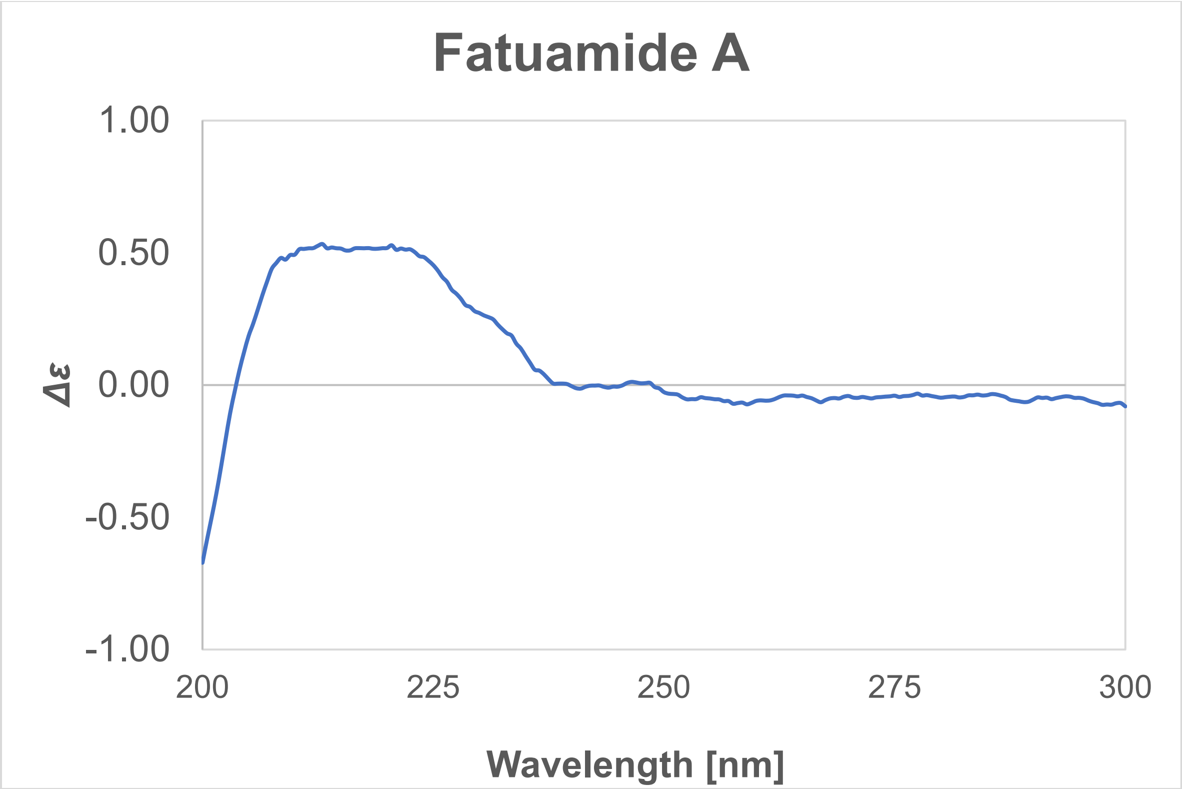

Figure S36. ECD spectrum of fatuamide A (**1**) in MeOH (1 mg/mL; 1.22 mM).

Table S1. A. Analysis of putative gene cluster for fatuamide B biosynthesis. The biosynthesis for fatuamide B starts with the biosynthesis of fatuamide A as described above (Figure S10) and in the main paper text. It then follows with fat S, an NRPS module that adds aspartic acid. Fat R is a TauD/TdfA family dioxygenase that is proposed to add a β-OH group to the aspartic acid. β-Hydroxylation by a TauD/TdfA family dioxygenase is common in siderophores.[10] Fat T is an NRPS module that is proposed to add a threonine residue. Fat T has an additional TauD/TdfA family dioxygenase and fat U is another NRPS module that codes for an aspartic acid; together, they are proposed to add a second β-OH aspartic acid to the chain. Fat V is a PKS module that encodes for a ketide extension and no reduction of the β-ketone functionality. Fat W is an NRPS module that is proposed to add a proline residue. Fat W has two condensation units. The second condensation unit is proposed to release fatuamide B much like the second condensation unit in Fat Q releases fatuamide A as discussed in the main text. The incorporated residues predicted from the putative biosynthetic gene cluster are in agreement with the mass spectrometry results depicted in Figure S9 for fatuamide B. The genes predicted to be involved in fatuamide A biosynthetic assembly are noted by the blue bar on the right side of the table and fatuamide B biosynthetic assembly are noted by the red bar on the right side of the table.

Label	nt length	Gene	Proposed Function (Antismash)	Similar Sequence	identity	Coverage	E-value	Accession number
ctg11_106	2265		Capase domain	caspase family protein, Coleofasciculus chthonoplastes	54%	99%	0	WP_006103464.1
ctg11_105	450			hypothetical protein, Oscillatoria sp. CS-180	59%	99%	1.00E-56	WP_272064066.1
ctg11_104	1041		Fructose-biphosphate aldolase class-I	class I fructose-bisphosphate aldolase, Oscillatoria sp. CS-180	88%	99%	0	WP_272064067.1
ctg11_103	336			2088 domain-containing protein [Chloroflexota bacterium]	31%	69%	0.036	MBE9472215.1
ctg11_102	969		Trypsin	trypsin-like peptidase domain-containing protein, Nodosilinea nodulosa	74%	94%	8.00E-161	WP_071527436.1
ctg11_101	2499		transcriptional regulator, SARP, family	tetratricopeptide repeat protein, Leptolyngbya sp. SIOISBB	67.96%	100%	0	NEQ47515.1
ctg11_100	228		Tetratricopeptide repeat	tetratricopeptide repeat protein, Nostoc sp. GBBB01	50%	86%	5.00E-06	MBL1202430.1
ctg11_99	612			no hits				
ctg11_98	135			no hits				
ctg11_97	813		4' phosphopantetheinyl transferase	4'-phosphopantetheinyl transferase superfamily protein, Leptolyngbya sp SIOISBB	66.41%	93.00%	3.00E-119	NEQ47703.1
ctg11_96	972		Protein of unknown function DUF3102	DUF3102 domain-containing protein, L:eptolyngbya sp. SIO1D8	79%	96%	1.00E-176	NER83354.1

ctg11_95	681		TPR repeat containing protein	tetratricopeptide repeat protein, Leptolyngbya sp. SIO1D8	81%	92%	4.00E -106	NER83355.1
ctg11_94	390			hypothetical protein, Leptolyngbya sp. SIO1D8	79%	89%	9.00E -63	NER84472.1
ctg11_93	354		Core binding factor beta subunit	MbtH domain protein, Pleurocapsales cyanobacterium LEGE 06147	82%	96%	5.00E -62	MBE9170816.1
ctg11_92	231		MbtH-like protein	MbtH family protein, Halomicronema hongdechloris	77%	93%	1.00E -35	WP_080813660. 1
ctg11_91	768	fatA	thioesterase	thioesterase, Pleurocapsales cyanobacterium LEGE 06147	73.52 %	99.00%	5.00E -134	MBE9170818.1
ctg11_90	249	fatB		acyl carrier protein, Leptolyngbya sp. SIO1D8	78%	92%	5.00E -32	NER81856.1
ctg11_89	459	fatC	4' phosphopantethein yl transferase superfamily	4'-phosphopantetheinyl transferase superfamily protein, Leptolyngbya sp SIO1D8	70%	97%	2.00E -74	NER81857.1
ctg11_88	1539	fatD	AMP-dependent synthetase and ligase	benzoate-CoA ligase family protein, Leptolyngbya sp SIO1D8	87%	100%	0	NER81858.1
ctg11_87	1323	fatE	isochorismate synthase	Salicylate synthase, Leptolyngbya sp SIO1D8	88%	96%	0	NER81859.1
ctg11_86	486	fatF		TPA: holo-[acyl-carrier-protein] synthase, Cyanobacteria bacterium UBA 11371	50%	95%	2E-49	HAZ44672.1
ctg11_85	1524	fatG	3-oxoacyl-(acyl carrier protein) synthase	3-oxoacyl-[acyl-carrier-protein] synthase, KAS III, uncultured Coleofasciculus sp.	69.35 %	99.00%	0	CAA9297985.1
ctg11_84	3474	fatH	C (heterocyclization), A- Cys, P	BarG, Anabaena cylindrica PCC 7122	65.00 %	100.00%	0	AP018166.1
ctg11_83	7944	fatI	KS, AT, cMT, KR, DH, ER, PCP	cis-AT_polyketide_synthase, Nostoc sp. Peltigera membranacea cyanboint	42%	99.30%	0	GQ979609.2
ctg11_82	7944	fatJ	KS, AT, cMT, KR, DH, ER, PCP	cis-AT_polyketide_synthase, Nostoc sp. Peltigera membranacea cyanboint	43%	98.00%	0	GQ979609.2
ctg11_81	6654	fatK	KS, AT, KR, DH, ER, PCP	cis-AT_polyketide_synthase, Nostoc sp. Peltigera membranacea cyanboint	52%	99.00%	0	GQ979609.2
ctg11_80	3456	fatL	C, A-Phe, P	nonribosomal protein synthetase, Anabaena cylindrica PCC7122	56%	96.40%	0	AP018166.1
ctg11_79	4650	fatM	KS, AT, KR, PCP	type I polyketide synthase, Fischerella sp. PCC 9431	55.00 %	99.40%	0	NZ_KE650771. 1.
ctg11_78	4638	fatN	KS, AT, KR, PCP	type I polyketide synthase, Fischerella sp. PCC 9431	56%	98.40%	0	NZ_KE650771. 1.
ctg11_77	3678	fatO	C, A-Phe, P	nonribosomal protein synthetase, Anabaena cylindrica PCC7122	53%	96%	0	AP018166.1

ctg11_76	4644	fatP	KS, AT, KR, PCP	type I polyketide synthase, Fischerella sp. PCC 9431	59%	98.80%	0	NZ_KE650771.1.
ctg11_75	4755	fatQ	C, A-gly, P, C	NcpA, Nostoc sp. ATCC 53789	55%	96.80%	0	AY167420.1
ctg11_74	1005	fatR	Dioxygenase TauD/TfdA	TauD/TfdA family dioxygenase, Leptolyngbya SIOiD8	81.68 %	99.00%	0	NER81770.1
ctg11_73	4716	fatS	C, A-Asp, PCP, E, X,	amino acid adenylation_enzyme/ thioester reductase family protein, thioester reductase like protein, Rivularia sp PCC 7116	51%	99.60%	0	CP003549.1
ctg11_72	7029	fatT	Nterm, C, A-thr (D), PCP, E, X, C, tauD	non-ribosomal_peptide_synthase/ polyketide synthase (Nostoc sp. UHCC 0702)	45%	86.50%	0	CP071065.1
ctg11_71	3318	fatU	C, A-Asp, PCP,	non-ribosomal_peptide_synthase/ amino acid adenylation enzyme, Rivularia sp PCC 7116	48%	96.70%	0	CP003549.1
ctg11_70	4542	fatV	KS, AT, PCP, E, X	cis-AT polyketide synthase, Nostoc cp. Peltigera membranacea cyanboint	63%	59.40%	0	GQ979609.2
ctg11_69	4596	fatW	Nterm, C(D), A-pro, PCP, C	non-ribosomal_peptide_synthase/ polyketide synthase (Nostoc sp. UHCC 0702)	51%	99.50%	0	CP071065.1
ctg11_68	1422	fatX	aminotransferase clase III	aspartate aminotransferase family protein, Aphanothece sp CMT-38BRIN-NPC111	60.85 %	99.00%	0	MBW4575232.1
ctg11_67	1242		Major Facilitator Transporter	MFS transporter, Leptolyngbya sp SIO1D8	77.17 %	97.00%	0	NER79552.1
ctg11_66	366			DUF4158 domain-containing protein, Stenomitos rutilans HA7619-LMS	40%	52%	0.0003	MBW4475173.1
ctg11_65	1038		iron compound ABC transporter, periplasmic	periplasmic binding protein, Leptolyngbya sp Heron Island J	58.68 %	91.00%	7.00E-129	ESA33711.1
ctg11_64	2613		TonB-dependent siderophore receptor	TonB-dependent receptor, Leptolyngbya SIOISBB	65.45 %	94.00%	0	NEQ42340.1
ctg11_63	852			dienelactone hydrolase , Nodosilinea sp. P-1105	72%	97%	5.00E-146	WP_169614874.1
ctg11_62	2787		LuxR family transcriptional regulator	LuxR C-terminal related transcriptional regulator, Leptolyngbyaceae cyanobacterium MO_188.B28	86.53 %	100.00%	0	MDJ0707777.1
ctg11_61	1733			pentapeptide repeat containing protein, Cyanobacteriota bacterium	41%	60%	7.00E-82	MBF2027584.1
ctg11_60	264			Txe/YoeB family addiction module toxin, Leptolyngbya sp. L.K.	87%	100%	6.00E-49	WP_039726605.1
ctg11_59	603			Uma2 endonuclease, Oscillatoria sp. CS-180	94.50 %	100.00%	6.00E-139	WP_272062562.1

ctg11_58	1206		transcriptional regulator, MerR family	MerR family transcriptional regulator, Oscillatoria sp. Cs-180	94.46 %	99.00%	0	WP_272062561.1
ctg11_57	441			hypothetical protein, Leptolyngbya sp SIO4C1	91.78 %	100.00%	2.00E -93	NEP20341.1
ctg11_56	498			cupin domain-containing protein, Oscillatoria sp CS-180	96%	100%	2.00E -112	WP_272064121.1
ctg11_55	1098			DUF3179 domain-containing protein, Chloroflexota bacterium	49%	100%	2.00E -116	HET59412.1
ctg11_54	645		methyltransferase	methyltransferase domain-containing protein, Cyanobacteria bacterium GSL.Bin1	86.32 %	99.00%	2.00E -134	NBD15079.1

B. Proposed biosynthesis of fatuamide B.

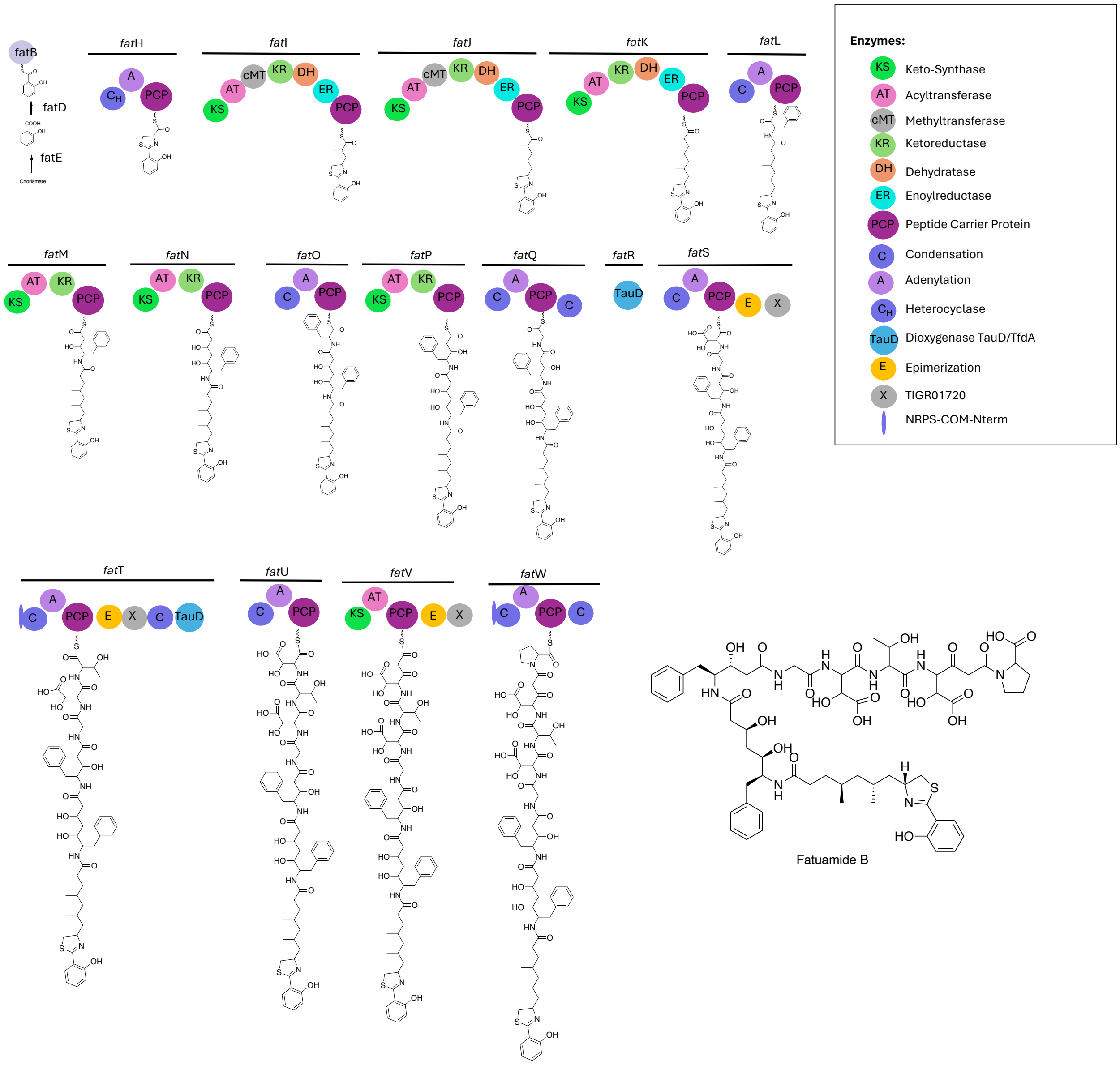

Table S2. Results of conformational search for *RR*-**1m**, *RS*-**1m**, *SR*-**1m**, and *SS*-**1m**.

	RR-**1m**	*RS*-**1m**	*SR*-**1m**	*SS*-**1m**
Non-duplicated conformers from grid search	4308	4663	2988	2965
Conformers within 4.5 kcal/mol from the lowest energy one	320	301	340	453
Non duplicated conformers optimized at B3LYP/6-31G(d)	224	211	183	227
Conformers used for NMR prediction	51	66	62	79

Table S3. Experimental chemical shifts, predicted chemical shifts, chemical shift errors, root mean square deviations (RMSD) of predicted chemical shifts, and mean absolute errors (MAE) of predicted chemical shift for *RR*-**1m**, *RS*-**1m**, *SR*-**1m**, and *SS*-**1m**. All values are expressed in ppm.

	Experimental	*RR*-**1m**		*RS*-**1m**		*SR*-**1m**		*SS*-**1m**	
		Predicted	Error	Predicted	Error	Predicted	Error	Predicted	Error
C-1	160.2	159.8	–0.4	160.4	0.2	159.7	–0.5	159.9	–0.3
C–2	117.8	116.0	–1.8	115.9	–1.9	115.9	–1.9	115.9	–1.9
C–3	134.0	134.3	0.3	134.1	0.1	134.3	0.3	134.3	0.3
C–4	120.0	117.4	–2.6	116.7	–3.3	117.4	–2.6	117.2	–2.8
C–5	131.6	132.2	0.6	132.3	0.7	132.3	0.7	132.2	0.6
C–6	117.6	115.5	–2.1	115.3	–2.3	115.6	–2.0	115.5	–2.1
C–9	75.5	72.9	–2.6	73.4	–2.1	73.9	–1.6	73.5	–2.0
C–10	44.3	43.6	–0.7	44.3	0.0	43.4	–0.9	44.7	0.4
C–11	29.3	29.1	–0.2	29.0	–0.3	30.0	0.7	29.8	0.5
C–12	20.3	20.8	0.5	19.6	–0.7	20.7	0.4	19.4	–0.9
C–13	45.1	41.5	–3.6	43.9	–1.2	44.7	–0.4	44.4	–0.7
C–14	31.1	30.0	–1.1	31.0	–0.1	31.5	0.4	30.2	–0.9
C–15	19.4	21.4	2.0	19.3	–0.1	20.7	1.3	21.5	2.1
C–16	35.0	31.5	–3.5	34.6	–0.4	34.8	–0.2	31.8	–3.2
C–17	35.0	33.8	–1.2	35.8	0.8	37.1	2.1	34.4	–0.6
	**^{13}C RMSD**		**1.56**		**1.36**		**1.32**		**1.48**
	**^{13}C MAE**		**1.23**		**0.95**		**1.07**		**1.11**
H–2	6.93	7.05	0.12	7.00	0.07	7.04	0.11	7.04	0.11
H–3	7.35	7.43	0.08	7.40	0.05	7.43	0.08	7.43	0.08
H–4	6.89	7.02	0.13	6.98	0.09	7.01	0.12	7.01	0.12
H–5	7.42	7.45	0.03	7.42	0.00	7.43	0.01	7.43	0.01
H–8a	3.02	3.00	–0.02	3.04	0.02	2.98	–0.04	2.99	–0.03
H–8b	3.52	3.41	–0.11	3.40	–0.12	3.39	–0.13	3.42	–0.10
H–9	4.76	4.67	–0.09	4.68	–0.08	4.65	–0.11	4.69	–0.07
H–10a	1.59	1.58	–0.01	1.57	–0.02	1.45	–0.14	1.49	–0.10
H–10b	1.69	1.75	0.06	1.74	0.05	1.72	0.03	1.61	–0.08
H–11	1.78	1.90	0.12	1.86	0.08	1.95	0.17	1.99	0.21
H–13a	1.11	1.06	–0.05	1.19	0.08	1.15	0.04	1.16	0.05
H–13b	1.25	1.46	0.21	1.25	0.00	1.17	–0.08	1.19	–0.06
H–14	1.47	1.73	0.26	1.55	0.08	1.48	0.01	1.68	0.21
H–16a	1.20	1.48	0.28	1.45	0.25	1.35	0.15	1.51	0.31
H–16b	1.41	1.74	0.33	1.57	0.16	1.63	0.22	1.69	0.28
H–17a	2.07	2.05	–0.02	2.13	0.06	2.07	0.00	2.09	0.02
H–17b	2.07	2.08	0.01	2.09	0.02	2.11	0.04	2.04	–0.03
H_3–12	0.94	0.99	0.05	0.97	0.03	1.02	0.08	1.04	0.10
H_3–15	0.84	0.94	0.10	0.91	0.07	0.91	0.07	0.94	0.10
	**^{1}H RMSD**		**0.116**		**0.090**		**0.104**		**0.136**
	**^{1}H MAE**		**0.088**		**0.070**		**0.087**		**0.093**

Table S4. Absorbance of the samples from the CAS assay at 655 nm. A decrease in the absorbance value indicates positive siderophore activity.

Sample ID	Sample	Abs @ 655.0
ASX22	Cyanobacteria	0.0642
ASX22 + CAS	Cyanobacteria + CAS	0.3448
CAS + H20	Control (CAS + H20)	3.0376
CAS + media	Control (CAS + media)	1.9166
CAS	Control (CAS only)	3.7858

Table S5. **A.** Five-point grading for assessing the visually observed health and viability of ASX22JUL4-2 cultures over 14 days in culture media with varying concentrations of dissolved copper performed at least in triplicate biological replicates. Natural seawater concentration set as 2 μg/L. Visual grading was done by two trained observers on Days 0, 2, 5, 7, 9, 12, and 14 and the average of the scores was used for the analysis.

GRADE	VISUAL DESCRIPTION
5	Healthy, vibrant cyanobacterial culture with no bleaching noted.
4	Some bleaching noted at the edges of the cyanobacterial culture.
3	Heterogeneous bleaching affecting multiple portions of the cyanobacterial culture.
2	Cyanobacterial biomass with near complete bleaching.
1	Complete, homogenous bleaching of the cyanobacterial culture.

Relative Conc. Of Copper Compared to Natural Seawater	Replicate #	Day 0	Day 0	Day 2	Day 2	Day 5	Day 5	Day 7	Day 7	Day 9	Day 9	Day 12	Day 12	Day 14	Day 14
0	1	5	5	N/A	5	5	5	5	5	5	5	5	5	5	5
0	2	5	5	N/A	5	5	5	5	5	5	5	5	5	5	5
0	3	5	5	N/A	5	5	5	5	5	5	5	5	5	5	5
0	4	5	5	5	5	5	5	5	5	5	5	5	5	5	5
0	5	5	5	5	5	5	5	5	5	5	5	5	5	5	5
0	6	5	5	5	5	5	5	5	5	5	5	5	5	5	5
0.5	1	5	5	N/A	5	5	5	5	5	5	5	5	5	5	5
0.5	2	5	5	N/A	5	5	5	5	5	5	5	5	5	5	5
0.5	3	5	5	N/A	5	5	5	5	5	5	5	5	5	5	5
1	1	5	5	N/A	5	5	5	5	5	5	5	5	5	5	5
1	2	5	5	N/A	5	5	5	5	5	5	5	5	5	5	5
1	3	5	5	N/A	5	5	5	5	5	5	5	5	5	5	5
1.5	1	5	5	N/A	5	5	5	5	5	5	5	5	5	5	5
1.5	2	5	5	N/A	5	5	5	5	5	5	5	5	5	5	5
1.5	3	5	5	N/A	5	5	5	5	5	5	5	5	5	5	5
2	1	5	5	N/A	5	5	5	5	5	5	5	5	5	5	5
2	2	5	5	N/A	5	5	5	5	5	5	5	5	5	5	5
2	3	5	5	N/A	5	5	5	5	5	5	5	5	5	5	5
3	1	5	5	N/A	5	5	5	5	5	5	5	5	5	5	5
3	2	5	5	N/A	5	5	5	5	5	5	5	5	5	5	5
3	3	5	5	N/A	5	5	5	5	5	5	5	5	5	5	5
4	1	5	5	N/A	5	5	5	5	5	5	5	5	5	5	5
4	2	5	5	N/A	5	5	5	5	5	5	5	5	5	5	5
4	3	5	5	N/A	5	5	5	5	5	5	5	5	5	5	5
5	1	5	5	N/A	5	5	5	5	5	5	5	5	5	5	5
5	2	5	5	N/A	5	5	5	5	5	5	5	5	5	5	5
5	3	5	5	N/A	5	5	5	5	5	5	5	5	5	5	5
10	1	5	5	5	5	5	5	5	5	5	5	5	5	5	5
10	2	5	5	5	5	5	5	5	5	5	5	5	5	5	5
10	3	5	5	5	5	5	5	5	5	5	5	5	5	5	5

25	1	5	5	N/A	5	5	5	5	5	5	5	5	5	5	5
25	2	5	5	N/A	5	5	5	5	5	5	5	5	5	5	5
25	3	5	5	N/A	5	5	5	5	5	5	5	5	5	5	5
50	1	5	5	N/A	5	4	5	5	5	5	5	5	5	5	5
50	2	5	5	N/A	5	5	5	5	5	5	5	5	5	5	5
50	3	5	5	N/A	5	5	5	5	5	5	5	5	5	5	5
100	1	5	5	N/A	5	5	5	5	5	5	5	5	5	5	5
100	2	5	5	N/A	5	5	5	5	5	5	5	5	5	5	5
100	3	5	5	N/A	5	5	5	5	5	5	5	5	5	5	5
250	1	5	5	N/A	5	5	5	5	5	5	5	5	5	5	5
250	2	5	5	N/A	5	5	5	5	5	5	5	5	5	5	5
250	3	5	5	N/A	5	5	5	5	5	5	5	5	5	5	5
250	4	5	5	5	5	5	5	5	5	5	5	5	5	5	5
250	5	5	5	5	5	5	5	5	5	5	5	5	5	5	5
250	6	5	5	5	5	5	5	5	5	5	5	5	5	5	5
500	1	5	5	N/A	5	5	5	5	5	5	5	5	5	5	5
500	2	5	5	N/A	5	5	5	5	5	5	5	5	5	5	5
500	3	5	5	N/A	5	5	5	5	5	5	5	5	5	5	5
500	4	5	5	5	5	5	5	4	5	5	4	4	4	4	4
500	5	5	5	5	5	5	5	4	5	5	4	5	5	4	4
500	6	5	5	5	5	5	4	5	5	5	5	5	5	4	4
1000	1	5	5	N/A	5	5	5	5	5	5	5	5	5	5	5
1000	2	5	5	N/A	5	5	5	5	5	5	5	5	5	5	5
1000	3	5	5	N/A	5	5	5	5	5	5	5	5	5	5	5
1000	4	5	5	5	5	5	4	3	4	4	2	2	3	3	3
1000	5	5	5	5	5	4	5	5	4	5	5	5	4	3	3
1000	6	5	5	5	5	4	4	3	4	4	3	3	3	3	3
2500	1	5	5	5	5	4	4	3	3	4	3	3	3	2	2
2500	2	5	5	5	5	4	4	3	3	3	4	3	3	2	2
2500	3	5	5	5	5	4	4	3	3	3	3	3	3	2	2
5000	1	5	5	5	5	4	4	3	3	3	3	3	3	2	2
5000	2	5	5	4	5	4	4	3	3	3	3	3	3	2	2
5000	3	5	5	4	5	4	4	3	3	3	3	3	3	2	2
7500	1	5	5	4	3	3	4	3	3	3	3	3	3	2	2
7500	2	5	5	4	4	4	3	3	3	3	3	3	3	2	2
7500	3	5	5	4	4	3	3	3	3	3	3	3	3	2	2
10000	1	5	5	5	5	3	3	3	3	3	3	3	3	2	2
10000	2	5	5	4	4	3	3	3	3	3	3	3	3	2	2
10000	3	5	5	4	4	3	3	3	3	3	3	3	2	2	2
25000	1	5	5	3	3	3	3	3	3	3	3	2	2	2	2
25000	2	5	5	3	3	3	3	3	3	3	3	2	2	2	2
25000	3	5	5	3	3	3	3	3	3	3	3	2	2	2	2

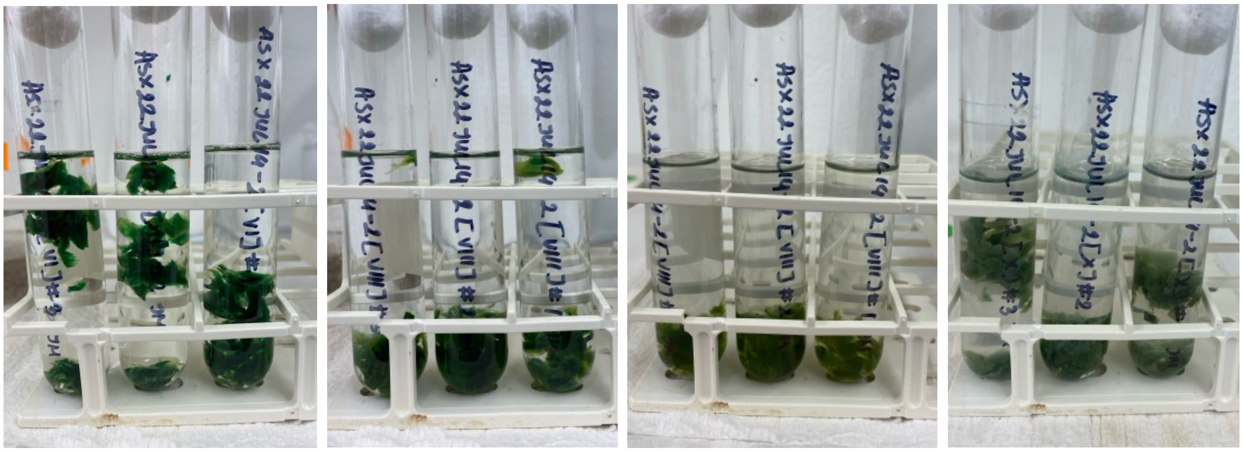

Grade 5 | Grade 4 | Grade 3 | Grade 2

Table S5 B. Example photos of cultures representing Grade 5, Grade 4, Grade 3, and Grade 2 of the scale used to visually assess health and viability of ASX22JUL14-2 (**Table S5 A**). Grade 5 represents healthy, vibrant cyanobacterial cultures with no bleaching noted. Grade 4 represents cultures with some bleaching noted at the edges of the biomass. Grade 3 represents cultures with heterogenous bleaching affecting multiple portions of the cyanobacterial culture. Grade 2 represents cyanobacterial biomass with near complete bleaching. Grade 1 represents cultures with complete, homogenous bleaching. There were no cultures identified as Grade 1 during the experiment, which would be nearly colorless. Increases in the opacity of the media is often seen as cultures become less healthy. This is notable in the Grade 3 and Grade 2 examples above.

References

(1) Wang, M.; Carver, J. J.; Phelan, V. V.; Sanchez, L. M.; Garg, N.; Peng, Y.; Nguyen, D. D.; Watrous, J.; Kapono, C. A.; Luzzatto-Knaan, T.; Porto, C.; Bouslimani, A.; Melnik, A. V.; Meehan, M. J.; Liu, W. T.; Crüsemann, M.; Boudreau, P. D.; Esquenazi, E.; Sandoval-Calderón, M.; Kersten, R. D.; Bandeira, N.; et al. Sharing and Community Curation of Mass Spectrometry Data with Global Natural Products Social Molecular Networking. *Nat. Biotechnol.* **2016**, *34* (8), 828–837. https://doi.org/10.1038/nbt.3597.

(2) Dührkop, K.; Fleischauer, M.; Ludwig, M.; Aksenov, A. A.; Melnik, A. V.; Meusel, M.; Dorrestein, P. C.; Rousu, J.; Böcker, S. SIRIUS 4: A Rapid Tool for Turning Tandem Mass Spectra into Metabolite Structure Information. *Nat. Methods* **2019**, *16* (4), 299–302. https://doi.org/10.1038/s41592-019-0344-8.

(3) Drechsel, H.; Stephan, H.; Lotz, R.; Haag, H.; Zähner, H.; Hantke, K.; Jung, G. Structure Elucidation of Yersiniabactin, a Siderophore from Highly Virulent Yersinia Strains. *Liebigs Ann.* **1995**, *1995* (10), 1727–1733. https://doi.org/10.1002/jlac.1995199510243.

(4) Zdouc, M. M.; Blin, K.; Louwen, N. L. L.; Navarro, J.; Loureiro, C.; Bader, C. D.; Bailey, C. B.; Barra, L. et al. "MIBiG 4.0: advancing biosynthetic gene cluster curation through global collaboration", *Nucleic Acids Research* **2025**, *53*, D678-D690, https://doi.org/10.1093/nar/gkae1115

(5) Kwan, D. H.; Leadlay, P. F. Mutagenesis of a Modular Polyketide Synthase Enoylreductase Domain Reveals Insights into Catalysis and Stereospecificity. *ACS Chem. Biol.* **2010**, *5* (9), 829–838. https://doi.org/10.1021/cb100175a.

(6) Schmidt, Y.; Breit, B. Direct Assignment of the Relative Configuration in 1,3, n-Methyl-Branched Carbon Chains by 1H NMR Spectroscopy. *Org. Lett.* **2010**, *12* (10), 2218–2221. https://doi.org/10.1021/ol1005399.

(7) Novak, T.; Tan, Z.; Liang, B.; Negishi, E. I. All-Catalytic, Efficient, and Asymmetric Synthesis of α,ω- Diheterofunctional Reduced Polypropionates via "One-Pot" Zr-Catalyzed Asymmetric Carboalumination - Pd-Catalyzed Cross-Coupling Tandem Process. *J. Am. Chem. Soc.* **2005**, *127* (9), 2838–2839. https://doi.org/10.1021/ja043534z.

(8) Brand, G. J.; Studte, C.; Breit, B. Iterative Synthesis of (Oligo) Deoxypropionates via Cross-Coupling. *Org. Lett.* **2009**, *3* (7), 9–11.

(9) Reiss, T.; Breit, B. A Unified Strategy for the Stereospecific Construction of Propionates and Acetate-Propionates Relying on a Directed Allylic Substitution. *Chem. - A Eur. J.* **2009**, *15* (26), 6345–6348. https://doi.org/10.1002/chem.200901064.

(10) Reitz, Z. L.; Hardy, C. D.; Suk, J.; Bouvet, J.; Butler, A. Genomic Analysis of Siderophore β-Hydroxylases Reveals Divergent Stereocontrol and Expands the Condensation Domain Family. *Proc. Natl. Acad. Sci. U. S. A.* **2019**, *116* (40), 19805–19814. https://doi.org/10.1073/pnas.1903161116.
